# T2DM may exert a protective effect against digestive system tumors in East Asian populations: a Mendelian randomization analysis

**DOI:** 10.3389/fonc.2024.1327154

**Published:** 2024-06-14

**Authors:** Ni An, Yu Zhang, Zhilin Sha, Zhen Xu, Xiuzhen Liu

**Affiliations:** ^1^ Department of Anesthesiology, The Eighth Medical Center of Chinese PLA General Hospital, Beijing, China; ^2^ No.91126 Military Hospital of Chinese PLA, Dalian, China; ^3^ Department I of Biliary Tract Surgery, Eastern Hepatobiliary Surgery Hospital, Naval Medical University, Shanghai, China

**Keywords:** type 2 diabetes mellitus, digestive system tumors, Mendelian randomization, causal relationship, East Asian population

## Abstract

**Introduction:**

Type 2 diabetes mellitus (T2DM) was associated with digestive system tumors. We analyzed publicly available data from GWAS studies using Mendelian randomization methods to clarify its causal relationship and mechanisms. Five common digestive system tumors and four diabetes-related phenotypes were included.

**Methods:**

Inverse variance weighted method was the main analytical method. Meta-analysis was used to summarize results of multiple data sources. Horizontal pleiotropy was tested using Egger-intercept method and validated by MRPRESSO method. Heterogeneity and sensitivity analysis were conducted by Cochran’s Q test and leave-one-out method, respectively.

**Results:**

T2DM is associated with a reduced risk of esophageal (OR: 0.77, 95% CI: 0.71 to 0.83, P< 0.001), gastric (OR: 0.87, 95% CI: 0.84 to 0.90, P< 0.001) and colorectal cancer (OR: 0.88, 95% CI: 0.85 to 0.91, P< 0.001) and hepatocellular carcinoma (OR: 0.92, 95% CI: 0.86 to 0.97, P = 0.005) and an increased risk of pancreatic cancer (OR: 1.92, 95% CI: 1.47 to 2.50, P< 0.001) in East Asian population. T2DM causes decreased fasting insulin levels (OR = 0.966, 95% CI: 0.95 to 0.98, P< 0.001) and increased glycated hemoglobin levels (OR=1.41, 95% CI: 1.39 to 1.44, P<0.001). Elevated fasting insulin levels increase the risk of esophageal cancer (OR = 10.35, 95% CI: 1.10 to 97.25, P = 0.041), while increased glycated hemoglobin levels increase pancreatic cancer risk (OR=2.33, 95% CI: 1.37 to 3.97, P=0.002) but decrease gastric cancer risk (OR=0.801, 95% CI: 0.65 to 0.99, P=0.044).

**Conclusion:**

T2DM is associated with a reduced risk of esophageal, gastric and colorectal cancer and hepatocellular carcinoma in East Asian populations. The causal relationships between T2DM with esophageal and gastric cancer are partially mediated by decreased fasting insulin and increased glycated hemoglobin levels, respectively. T2DM indirectly increases the risk of pancreatic cancer by increasing glycated hemoglobin levels.

## Highlights

- To clarify the causal relationship and mechanisms between T2DM and five digestive system tumors.
- Is T2DM a risk factor for digestive tract tumors in East Asian population?
- T2DM reduced the risks of esophageal, gastric and colorectal cancer and hepatocellular carcinoma in East Asian population.
- T2DM may have different impacts on different ethnicities.

## Introduction

The prevalence of diabetes is increasing worldwide yearly (1) with its age at onset decreasing as shown by several studies (2). The attention to type 2 diabetes mellitus (T2DM) and cancer has increased and led to a rapid proliferation of observation studies. Multiple large-scale epidemiological studies and meta-analyses have found an association between T2DM and various cancers including colorectal cancer, liver cancer and pancreatic cancer (3–5).

However, there are many methodological challenges that must be addressed in observational studies of cancer incidence in people with T2DM, including the usual suspects of potential biases or confounding factors that threaten the validity of studies and also the potential interactions between T2DM and cancer (6). For example, some studies found that the incidences of multiple cancers, including colorectal, lung, liver, cervical, endometrial, ovarian, pancreatic and prostate cancers, were greater within the first months to years following the diagnosis of diabetes, however, after the initial period, the risks of lung, cervical and endometrial cancers in participants with diabetes were the same as those observed in participants without (7). These results suggested that the likelihood of developing cancer was increased by the fact that diabetes was recently identified. A retrospective study from Australia has reported similar results in breast cancer (8). It remains unclear whether T2DM is causally related to cancer, or whether the observed association is confounded by other factors.

T2DM is an insidious condition, with its onset typically recognized in older adults. The potential biological mechanisms for the association between diabetes and cancer include hyperinsulinemia, hyperglycemia and insulin resistance (6, 9). Hyperglycemia is responsible for the induction of oxidative stress and DNA damage, which may trigger the first phase of tumorigenesis (9). Hyperglycemia may also contribute to the generation of advanced glycation end products (AGEs) that stimulates the production of reactive oxygen species and inflammation (10). Chronic activation of the AGEs pathway has been shown to promote the tumor transformation of epithelial cells and the resistance of tumor cells to oxidative stress (10, 11). However, evidence from the large RCTs of intensified glycemic control for T2DM does not support the causal hypothesis that lowering blood glucose will reduce the risk of cancer, and the accumulating experimental and epidemiological evidence is more consistent with the hyperinsulinemia hypothesis, and less so with the hyperglycemia hypothesis (6). Hyperinsulinemia promotes tumor cell growth via insulin receptor directly and IGF-1 receptor indirectly (12), all of which can stimulate the proliferation and survival of cancer cells and promote metastasis, thus favoring cancer progression (9, 13). Insulin stimulation from increased expression of insulin receptors may result in enhanced proliferation with loss of cell contact inhibition, and cancer cells frequently show augmented insulin receptor expression levels, mostly of the isoform A (lacking exon-11), whose activation is more responsible for mitogenic than metabolic effects and also shows high affinity for IGF-2 (9, 14). However, insulin resistance and hyperinsulinemia can predate the clinical diagnosis of T2DM by up to 10 years, thus the influence of this condition on cancer risk may begin well before diabetes diagnosis (6, 12).

A review in 2013 summarized the epidemiological, pathophysiological, genetical and socioeconomical factors in the differences of pathogenesis of T2DM between Asians and Caucasians, and found different characteristics of diabetes in Asians. Comparing with Caucasians, Asians with T2DM have lower mean BMI, greater adiposity or visceral fat and more insulin resistant, also show a tendency to develop young-onset diabetes and a predisposition to impaired insulin secretion, besides, they also have different lifestyles and environmental risk factors (1). These differences raised the need for re-examining the association between cancer and T2DM in Asian.

The genetic alleles associated with exposure are randomly distributed during conception, independent of self-selected lifestyle and environmental factors, and unaffected by disease interference. Mendelian randomization (MR) analysis leverages this characteristic by using genetic variants as instrumental variables to mitigate the influence of confounding factors such as environmental factors, lifestyle changes and reverse causality, thereby strengthening the inference of association between exposure and outcome. Therefore, MR can be an appropriate way to figure out the causal relationship between T2DM and cancer. In this study, we conducted two-sample MR (TSMR) analyses using publicly available genome-wide association studies (GWAS) databases to explore the causal relationship between T2DM and diabetes-related manifestations such as hemoglobin A1c (HbA1c) protein and fasting insulin levels, with five digestive system malignancies including esophageal cancer, gastric cancer, colorectal cancer, liver and bile duct cancer, and pancreatic cancer.

## Materials and methods

### Study design

The present study primarily utilized the TSMR method to explore the causal relationship between T2DM and its manifestations with five common digestive system tumors including esophageal cancer, gastric cancer, colorectal cancer, liver and bile duct cancer, and pancreatic cancer, resorting genetic variable instruments. Through GWAS published in the Japan Biobank (BBJ), Finnish database (Finn database) and other publicly available resources, single nucleotide polymorphisms (SNPs) that are statistically significant associated with T2DM and its manifestations were screened as instrumental variables, and the TSMR approach was employed to assess the causal relationship between T2DM and its manifestations with the five aforementioned digestive system tumors.

MR analysis relies on the satisfaction of three assumptions: (1) The instrumental variables (IVs) should be strongly associated with the exposure; (2) The IVs should not be associated with confounding factors related to both exposure and outcome; (3) The IVs should only affect the outcome through the exposure rather than through other pathways.

### Two-sample Mendelian randomization

#### Data source

The genetic data used in this study was obtained from the IEU OpenGWAS project (mrcieu.ac.uk). The GWAS data information used in this study is shown in **Table 1**, and the summarized demographic data of the GWAS of 5 primary outcomes are shown in **Table 2**.

**Table 1:** Data characteristics of all included GWAS studies.

GWAS ID	Trait	year	Population	Sex	ncase	ncontrol	Sample size	Number of SNPs
bbj-a-107	Colorectal cancer	2019	East Asian	Males and Females	7,062	195,745	202,807	8,885,369
bbj-a-119	Gastric cancer	2019	East Asian	Males and Females	6,563	195,745	202,308	8,885,324
bbj-a-140	Pancreatic cancer	2019	East Asian	Males and Females	442	195,745	196,187	8,885,075
bbj-a-117	Esophageal cancer	2019	East Asian	Males and Females	4,050	208,403	212,453	8,885,805
bbj-a-158	hepatocellular carcinoma	2019	East Asian	Males and Females	1,866	195,745	197,611	8,885,115
ieu-a-1057	Gallbladder cancer	2012	East Asian	Males and Females	41	866	907	425,707
ieu-a-822	Pancreatic cancer	2009	European	Males and Females	1896	1939	3835	521863
finn-b-C3_PANCREAS_EXALLC	Malignant neoplasm of pancreas (all cancers excluded)	2021	European	Males and Females	605	174006	174611	16380306
finn-b-C3_STOMACH_EXALLC	Malignant neoplasm of stomach (all cancers excluded)	2021	European	Males and Females	633	174006	174639	16380305
finn-b-C3_OESOPHAGUS_EXALLC	Malignant neoplasm of oesophagus (all cancers excluded)	2021	European	Males and Females	232	174006	174238	16380304
ieu-b-4960	Oesophageal cancer	2021	European	Males and Females	740	372,016	372,756	8,970,465
finn-b-C3_COLON_ADENO_EXALLC	Colon adenocarcinoma (all cancers excluded)	2021	European	Males and Females	1396	174006	175402	16380311
finn-b-C3_COLORECTAL_EXALLC	Colorectal cancer (all cancers excluded)	2021	European	Males and Females	3022	174006	177028	16380321
ieu-b-4953	Liver cell carcinoma	2021	European	Males and Females	168	372,016	372,184	6,304,034
bbj-a-77	Type 2 diabetes	2019	East Asian	Males and Females	36,614	155,150	191,764	12,557,761
bbj-a-153	Type 2 diabetes	2019	East Asian	Males and Females	40,250	170,615	210,865	8,885,694
ebi-a-GCST010118	Type 2 diabetes	2020	East Asian	NA	77,418	356,122	433,540	11,222,507
ebi-a-GCST90002237	Fasting insulin	2021	East Asian	NA	NA	NA	29,792	13,550,620
bbj-a-26	Hemoglobin A1c	2019	East Asian	Males and Females	NA	NA	42,790	6,108,953
ebi-a-GCST90018735	Glucose levels	2021	East Asian	NA	NA	NA	133,336	12,500,242
ebi-a-GCST90002226	Two-hour glucose	2021	East Asian	NA	NA	NA	8,509	8,427,199
ukb-e-30740_EAS	Glucose	2020	East Asian	Males and Females	2,342	NA	2,342	8,259,748
finn-b-E4_DM2_STRICT	Type 2 diabetes, strict	2021	European	Males and Females	29166	183185	212351	16380434
ebi-a-GCST005413	Type 2 diabetes	2018	European	NA	12,931	57,196	70,127	14,277,791
ukb-a-75	Type 2 diabetes	2017	European	Males and Females	2,133	335,026	337,159	10,894,596
ukb-a-159	Treatment/medication code: metformin	2017	European	Males and Females	8,392	328,767	337,159	10,894,596
ukb-b-14609	Treatment/medication code: metformin	2018	European	Males and Females	11,552	451,381	462,933	9,851,867
ebi-a-GCST004939	Glycated hemoglobin levels	2017	European	NA	NA	NA	9,436	21,269,114
ebi-a-GCST90002244	Glycated hemoglobin levels	2021	European	NA	NA	NA	146,806	30,649,064

NA, Not Available.

**Table 2:** Summary Demographic Data of the GWAS of 5 Cancers.

Trait	GWAS ID	Total samples	Case samples			Control samples		
			*n* (total)	male%	Mean age	*n* (total)	male%	Mean age
Colorectal cancer	bbj-a-107	202807	7062	63.66%	66.99	195745	49.89%	61.56
Gastric cancer	bbj-a-119	202308	6563	74.43%	66.82	195745	49.89%	61.56
Hepatocellular carcinoma	bbj-a-158	197611	1866	74.17%	67.96	195745	49.89%	61.56
Esophageal cancer	bbj-a-117	197045	1300	87.08%	65.91	195745	49.89%	61.56
Pancreatic cancer	bbj-a-140	196187	442	65.16%	66.38	195745	49.89%	61.56

#### Instrumental variables selection

Single-nucleotide polymorphisms (SNPs) related to T2DM, Hemoglobin A1c, Fasting insulin, Blood sugar, Fasting glucose, and Two-hour glucose were selected as instrumental variables from IEU OpenGWAS project (mrcieu.ac.uk). The process of instrumental variable selection process in this study involves the following steps: (1) The SNPs were considered as strongly correlated with the exposure factor if P< 5×10^8^, and if no SNPs could be screened with this threshold, SNPs were considered as strongly correlated with the exposure factor if P< 5×10^6^. The F-statistics (15) were calculated using Equation 1, in which N represents the sample size of the GWAS analysis, k the number of instrumental variables, R^2^ (16) the extent to which instrumental variables explain the exposure factor, which was calculated using Equation 2, and F was set >10 to avoid weak instrumental variable bias. Maf, β and SE represents the minor allele frequency, the effect value of the SNP on the exposure factor and the standard error of β, respectively. (2) to avoid linkage disequilibrium (LD) caused by exposure-related SNPs, SNPs with linkage disequilibrium (LD) (R^2^ > 0.001, clump distance< 10000kb, P< 5×10^8^) were removed.


(1) $$F=\frac{N-k-1}{k}\times \frac{R^{2}}{1-R^{2}}$$

(2) $$R^{2}=2\times \left(1-Maf\right)\times Maf\times \frac{\beta }{SE\times \sqrt{N}}$$

### Statistical analysis

#### Software and R packages

In this study, mendelian randomization was conducted using the R software (version 4.2.2), the CRAN packages of TwoSampleMR (version 0.5.7) and MRPRESSO (version 1.0) for analysis. The study employed a two-tailed test with a significance level of α=0.05.

#### The statistical methods of TSMR

To quantify the strength of the association between exposure and outcome, inverse variance weighting (IVW), MR Egger, weighted median (WM), simple model and weighted model analyses were conducted, among which IVW analysis was used as the main result. If heterogeneity was detected by Cochran’s Q test, the IVW (multiplicative random effects) method was used. To distinguish causal effects from reverse causality, the MR Steiger directionality test (17) was used, and the result of “TRUE” means that the predicting association was in the expected orientation.

#### Multivariable MR (MVMR) to assess the direct causal effect

Previous studies have provided evidence that chronic hepatitis C infection and cirrhosis are 2 potential confounders influencing the incidence risk of hepatocellular carcinoma (18–21). Therefore, further summary results and additional IVs of these 2 confounders were extracted to perform an IVW-based MVMR to confirm the direct effect of T2DM with controlling for the effect of chronic hepatitis C infection and cirrhosis, respectively.

#### Sensitivity analysis

The heterogeneity of SNP effect sizes was evaluated by Cochran’s Q test, where P<0.05 indicates the presence of heterogeneity. The IVW random-effect model was used when P<0.05. The leave-one-out method was employed to assess the impact of each SNP on the results, checking if the results were robust. MR-Egger and MR Pleiotropy RESidual Sum and Outlier (MR-PRESSO) tests were used to test horizontal pleiotropy and outliers. The Egger-intercept method was used for pleiotropy test, which could estimate whether instrumental variables affect outcome through other paths than exposure. The intercept from the MR-Egger analysis can be interpreted as the average pleiotropic effect of a genetic variant included in the analysis (22, 23). There is no horizontal gene multiplicity in genetic variation if the intercept value is close to zero. The MR-PRESSO global test evaluates overall horizontal pleiotropy amongst all IVs in a single MR test by comparing the observed distance of all the variants to the regression line (residual sum of squares) to the expected distance under the null hypothesis of no horizontal pleiotropy (24). The MR-PRESSO test comprises three parts: (1) the MR-PRESSO global test detects directional horizontal pleiotropy, (2) the outlier-corrected causal estimate corrects the detected directional horizontal pleiotropy, and (3) the MR-PRESSO distortion test estimates whether the causal estimates differ significantly (P< 0.05) after adjustment for the outliers (24).

#### Meta-analysis of MR results

When there were multiple data sources for the same exposure and outcome factors, meta-analysis was conducted to summarize the results. The exposure and outcome data ID, outcome type, population ethnicity, number of instrumental variables, MR analysis method, OR value, P value and 95% confidence interval (CI) of each MR results were summarized as meta-analysis data. The R (version 4.2.2) meta package was used for meta-analysis. Heterogeneity analysis of included studies was evaluated using the Chi^2^ test, the heterogeneity among studies was low if P≥0.1 and I^2^ ≤ 50%, in such cases a fixed-effect model was used, otherwise, a random-effects model was used. The publication bias of the included studies was evaluated using the funnel plot method. All included indicators were subjected to two-tailed tests. The difference was considered statistically significant if P<0.05.

## Results

### Validity of the instrumental variables

To investigate the causal effects of T2DM on digestive system tumors, SNPs associated with T2DM-related exposures were identified and F-statistics were calculated. Inclusion criteria were explained in the methods section. The number of included SNPs for each data set and exposure is shown in **Table 3**. The F-statistic of each SNP was > 10, which indicated that no weak instrument bias existed (**Supplementary Tables 1–4**).

**Table 3:** The number of included SNPs of the instrumental variables.

exposure	no. SNPs strongly correlated with the exposure after removing SNPs with LDs	no. SNPs F-statistics< 10	no. SNPs included	mean F‐statistics
Type 2 diabetes || id:ebi-a-GCST010118	174	24	150	27.33
Type 2 diabetes || id:bbj-a-153	102	39	63	17.58
Type 2 Diabetes || id:bbj-a-77	86	24	62	18.62
Hemoglobin A1c || id:bbj-a-26	25	2	23	26.76
Fasting insulin || id:ebi-a-GCST90002237	7	4	3	44.83
Blood sugar || id:bbj-a-10	18	0	18	60.52
Fasting glucose || id:ebi-a-GCST90002231	15	2	13	43.79
Two-hour glucose || id:ebi-a-GCST90002226	16	8	8	12.19
Type 2 diabetes || id:ebi-a-GCST005413	16	1	15	49.91
Non-cancer illness code self-reported: type 2 diabetes || id:ukb-a-75	3	0	3	506.19
Type 2 diabetes, strict (exclude DM1) || id:finn-b-E4_DM2_STRICT	58	15	43	23.19

### The causal relationship between T2DM and digestive system tumors

**Figure 1:**
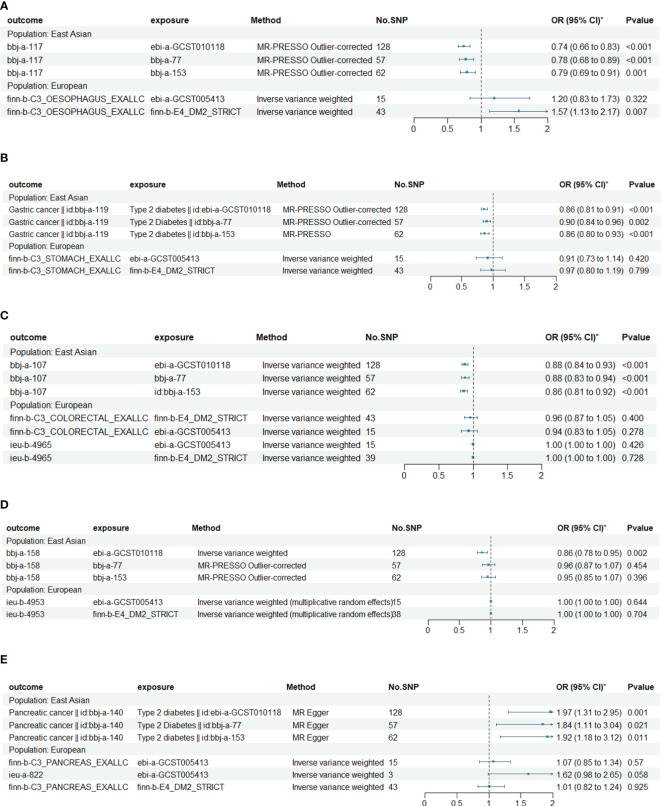
Causal relationships between T2DM and esophageal cancer, gastric cancer, colorectal cancer, hepatocellular carcinoma and pancreatic cancer in East Asian and European. **(A)** The Mendelian randomization (MR) effects of T2DM on esophageal cancer in European (estimated by IVW method) and East Asian populations (estimated by MR-PRESS method). **(B)** The MR effects of T2DM on gastric cancer in European (estimated by IVW method) and East Asian populations (estimated by MR-PRESS method). **(C)** The MR effects of T2DM on colorectal cancer in European and East Asian populations (estimated by IVW method). **(D)** The MR effects of T2DM on hepatocellular carcinoma in European and East Asian populations (estimated by IVW method). **(E)** The MR effects of T2DM on pancreatic cancer in European (estimated by IVW method) and East Asian populations (estimated by MR Egger method).

**Figure 2:**
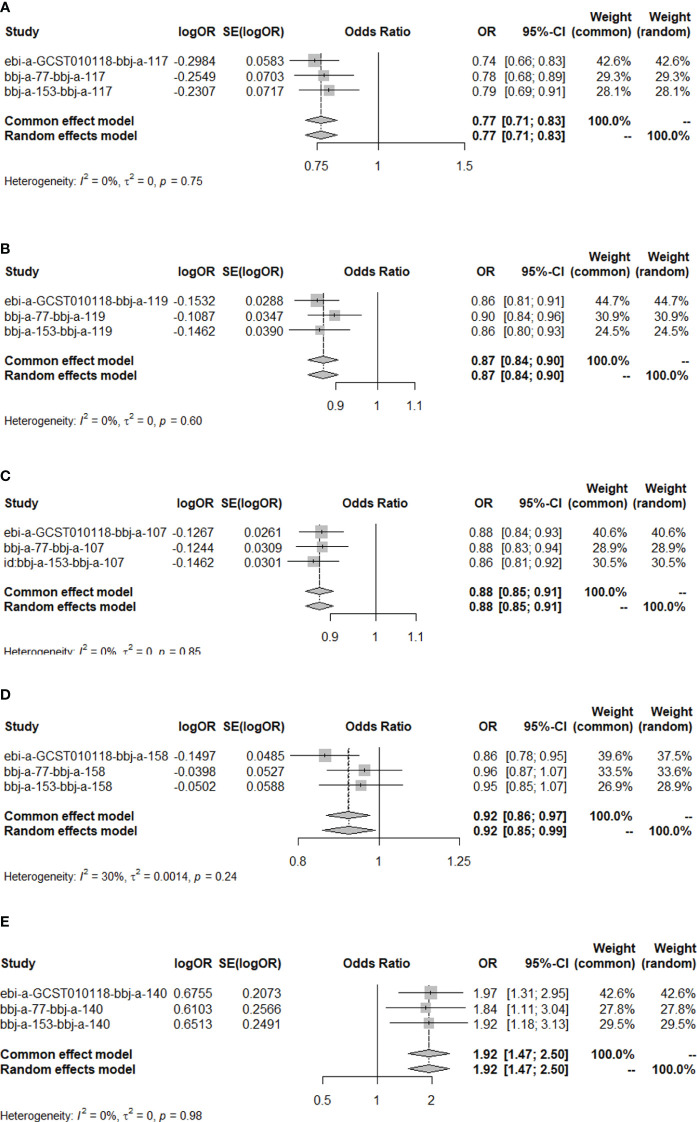
The MR results of T2DM on esophageal cancer, gastric cancer, colorectal cancer, hepatocellular carcinoma and pancreatic cancer in East Asian population. **(A)** Meta-analysis of the MR effects of T2DM on esophageal cancer in East Asian population (estimated by MR-PRESS method). **(B)** Meta-analysis of the MR effects of T2DM on gastric cancer in East Asian population (estimated by MR-PRESS method). **(C)** Meta-analysis of the MR effects of T2DM on colorectal cancer in East Asian population (estimated by IVW method). **(D)** Meta-analysis of the MR effects of T2DM on hepatocellular carcinoma in East Asian population (estimated by IVW method). **(E)** Meta-analysis of the MR effects of T2DM on pancreatic cancer in East Asian population (estimated by MR Egger method).

The results of the MR analysis on the causal relationship between T2DM and various digestive system tumors are shown in **Table 4**. Through meta-analysis of the MR results of the same outcomes, we found a causal relationship between T2DM with esophageal cancer, gastric cancer, colorectal cancer and hepatocellular carcinoma (**Figure 1**). However, there were differences among different ethnicities and outcomes. In East Asian population, T2DM was found to decrease the risk of esophageal cancer (OR=0.77, 95% CI: 0.71 to 0.83, P<0.001, **Figure 2A**), gastric cancer (OR=0.87, 95% CI: 0.84 to 0.90, P<0.001, **Figure 2B**), colorectal cancer (OR=0.88, 95% CI: 0.85 to 0.91, P<0.001, **Figure 2C**) and hepatocellular carcinoma (OR=0.92, 95% CI: 0.86 to 0.97, P=0.005, **Figure 2D**), however, T2DM was found to increase the risk of pancreatic cancer (OR=1.92, 95% CI: 1.47 to 2.50, P<0.001, **Figure 2E**). Whereas, in European population, no significant causal relationship was observed for the colorectal cancer or pancreatic cancer (**Supplementary Figure 1**, **Supplementary Table 5**). Using MR Steiger directionality test, no reverse association between T2DM and digestive system tumors was observed (**Supplementary Table 6**).

**Table 4:** Results of MR analysis of the causal effect of T2DM on digestive system tumors.

outcome	exposure	Method	No.SNP	OR (95% CI)	Pvalue	Q_pval Q’_pval RSS_pval	egger_ intercept	I Pvalue	No.Outliers	P value for MR-PRESSO distortion test
Colorectal cancer	Type 2 diabetes || id:ebi-a-GCST010118	MR Egger	128	0.85 (0.76 to 0.95)	0.006	0.049	0.003	0.503		
		Weighted median	128	0.88 (0.81 to 0.96)	0.002					
		IVW	128	0.88 (0.84 to 0.93)	0.000	0.052				
		MR-PRESSO	128	0.88 (0.84 to 0.93)	<0.001	0.048			0	
Colorectal cancer	Type 2 Diabetes || id:bbj-a-77	MR Egger	57	0.84 (0.74 to 0.97)	0.018	0.121	0.004	0.481		
		Weighted median	57	0.87 (0.80 to 0.96)	0.004					
		IVW	57	0.88 (0.83 to 0.94)	<0.001	0.129				
		MR-PRESSO	57	0.88 (0.82 to 0.94)	<0.001	0.093			0	
Colorectal cancer	Type 2 diabetes || id:bbj-a-153	MR Egger	62	0.85 (0.75 to 0.97)	0.016	0.112	0.002	0.763		
		Weighted median	62	0.87 (0.80 to 0.95)	0.002					
		IVW	62	0.86 (0.81 to 0.92)	<0.001	0.128				
		MR-PRESSO	62	0.87 (0.81 to 0.92)	<0.001	0.128			0	
Esophageal cancer	Type 2 diabetes || id:ebi-a-GCST010118	MR Egger	128	0.84 (0.65 to 1.10)	0.211	0.008	-0.007	0.453		
		Weighted median	128	0.77 (0.64 to 0.92)	0.003					
		IVW (multiplicative random effects)	128	0.77 (0.68 to 0.87)	<0.001	0.009				
		MR-PRESSO Outlier-corrected	128	0.74 (0.66 to 0.83)	<0.001	0.010			1	0.696
Esophageal cancer	Type 2 Diabetes || id:bbj-a-77	MR Egger	57	1.00 (0.71 to 1.41)	0.998	0.007	-0.021	0.194		
		Weighted median	57	0.78 (0.63 to 0.96)	0.017					
		IVW (multiplicative random effects)	57	0.81 (0.70 to 0.95)	0.009	0.006				
		MR-PRESSO Outlier-corrected	57	0.78 (0.68 to 0.89)	<0.001	0.006			1	0.667
Esophageal cancer	Type 2 diabetes || id:bbj-a-153	MR Egger	62	1.00 (0.71 to 1.40)	0.986	0.002	-0.018	0.235		
		Weighted median	62	0.81 (0.66 to 0.99)	0.038					
		IVW (multiplicative random effects)	62	0.83 (0.71 to 0.97)	0.018	0.001				
		MR-PRESSO Outlier-corrected	62	0.79 (0.69 to 0.91)	0.001	<0.001			1	0.640
Gastric cancer	Type 2 diabetes || id:ebi-a-GCST010118	MR Egger	128	0.83 (0.73 to 0.95)	0.006	0.001	0.003	0.576		
		Weighted median	128	0.88 (0.81 to 0.96)	0.006					
		IVW (multiplicative random effects)	128	0.86 (0.81 to 0.91)	<0.001	0.001				
		MR-PRESSO Outlier-corrected	128	0.86 (0.81 to 0.91)	<0.001	0.001			1	0.890
Gastric cancer	Type 2 Diabetes || id:bbj-a-77	MR Egger	57	0.91 (0.76 to 1.09)	0.324	<0.001	-0.003	0.712		
		Weighted median	57	0.84 (0.76 to 0.93)	<0.001					
		IVW (multiplicative random effects)	57	0.88 (0.82 to 0.96)	0.003	<0.001				
		MR-PRESSO Outlier-corrected	57	0.90 (0.84 to 0.96)	0.002	<0.001			4	0.124
Gastric cancer	Type 2 diabetes || id:bbj-a-153	MR Egger	62	0.85 (0.72 to 1.00)	0.051	<0.001	0.002	0.822		
		Weighted median	62	0.86 (0.78 to 0.95)	0.003					
		IVW (multiplicative random effects)	62	0.86 (0.80 to 0.93)	<0.001	<0.001				
		MR-PRESSO	62	0.86 (0.80 to 0.93)	<0.001	0.001			0	
Pancreatic cancer	Type 2 diabetes || id:ebi-a-GCST010118	MR Egger	128	1.97 (1.31 to 2.95)	0.001	0.306	-0.049	0.001		
		Weighted median	128	1.13 (0.82 to 1.56)	0.440					
		IVW	128	1.07 (0.88 to 1.30)	0.483	0.131				
		MR-PRESSO	128	1.07 (0.90 to 1.29)	0.435	0.166			0	
Pancreatic cancer	Type 2 Diabetes || id:bbj-a-77	MR Egger	57	1.84 (1.11 to 3.04)	0.021	0.253	-0.048	0.041		
		Weighted median	57	1.14 (0.80 to 1.60)	0.468					
		IVW	57	1.14 (0.90 to 1.44)	0.271	0.160				
		MR-PRESSO	57	1.19 (0.95 to 1.49)	0.136	0.125			0	
Pancreatic cancer	Type 2 diabetes || id:bbj-a-153	MR Egger	62	1.92 (1.18 to 3.12)	0.011	0.189	-0.045	0.041		
		Weighted median	62	1.41 (0.99 to 2.00)	0.056					
		IVW	62	1.21 (0.96 to 1.52)	0.108	0.114				
		MR-PRESSO	62	1.21 (0.96 to 1.52)	0.106	0.133			0	
hepatocellular carcinoma	Type 2 diabetes || id:ebi-a-GCST010118	MR Egger	128	0.93 (0.76 to 1.15)	0.523	0.121	-0.007	0.388		
		Weighted median	128	0.91 (0.78 to 1.05)	0.193					
		IVW	128	0.86 (0.78 to 0.95)	0.002	0.123				
		MR-PRESSO	128	0.89 (0.81 to 0.98)	0.014	0.099			0	
hepatocellular carcinom	Type 2 Diabetes || id:bbj-a-77	MR Egger	57	0.93 (0.69 to 1.25)	0.625	0.004	-0.004	0.787		
		Weighted median	57	0.92 (0.78 to 1.08)	0.305					
		IVW (multiplicative random effects)	57	0.90 (0.79 to 1.02)	0.098	0.005				
		MR-PRESSO Outlier-corrected	57	0.96 (0.87 to 1.07)	0.454	0.003			2	0.144
hepatocellular carcinom	Type 2 diabetes || id:bbj-a-153	MR Egger	62	0.90 (0.67 to 1.21)	0.492	<0.001	0.001	0.958		
		Weighted median	62	0.97 (0.83 to 1.15)	0.760					
		IVW (multiplicative random effects)	62	0.91 (0.79 to 1.04)	0.155	<0.001				
		MR-PRESSO Outlier-corrected	62	0.95 (0.85 to 1.07)	0.396	0.001			2	0.172

Q_Pvalue, for the Q test from IVW; Q_ Pvalue′, P-value for the Q′ test from MR-Egger; RSS_ Pvalue, P-value for MR-PRESSO global test; IVW, for Inverse variance weighted.

Considering chronic hepatitis C infection and cirrhosis could affect T2DM and play an important role in the pathogenesis of hepatocellular carcinoma, we conducted an MVMR to estimate a direct effect of T2DM on hepatocellular carcinoma accounting for their confounding effects. After adjusting for chronic hepatitis C infection (OR: 0.99, 95% CI: 0.89 to 1.09, P = 0.783) and cirrhosis (OR: 0.95, 95% CI: 0.88 to 1.04, P = 0.296), the effect of T2DM on hepatocellular carcinoma was not significant (**Supplementary Figure 2**, **Supplementary Table 7**).

Smoking, alcohol consumption, obesity, physical activity and sedentary behavior are common modifiable risk factors for gastrointestinal cancers in East Asia (25), all of which affect insulin secretion and action, and are major risk factors of T2DM (26). Limited by data resource, we conducted an IVW-based MVMR to estimate a direct effect of T2DM on digestive system tumors accounting for the confounding effect from smoking status, BMI, and Alcohol intake frequency (**Supplementary Table 8**). For colorectal cancer (smoking status adjusted: OR: 0.89, 95% CI: 0.84 to 0.93, P< 0.001; BMI adjusted: OR: 0.90, 95% CI: 0.85 to 0.95, P< 0.001; Alcohol intake frequency adjusted: OR: 0.89, 95% CI: 0.84 to 0.94, P< 0.001), esophageal cancer (smoking status adjusted: OR: 0.78, 95% CI: 0.68 to 0.90, P< 0.001; BMI adjusted: OR: 0.79, 95% CI: 0.66 to 0.94, P< 0.001; Alcohol intake frequency adjusted: OR: 0.78, 95% CI: 0.67 to 0.90, P< 0.001), gastric cancer (smoking status adjusted: OR: 0.88, 95% CI: 0.82 to 0.93, P< 0.001; BMI adjusted: OR: 0.88, 95% CI: 0.82 to 0.95, P< 0.001; Alcohol intake frequency adjusted: OR: 0.87, 95% CI: 0.82 to 0.92, P< 0.001), and hepatocellular carcinoma (smoking status adjusted: OR: 0.89, 95% CI: 0.80 to 0.99, P = 0.025; BMI adjusted: OR: 0.89, 95% CI: 0.79 to 0.99, P = 0.026; Alcohol intake frequency adjusted: OR: 0.89, 95% CI: 0.80 to 0.90, P = 0.026) the results of MVMR remained consistent with our primary findings. For pancreatic cancer, MVMR failed to find a significant effect of T2DM after adjustment smoking status (OR: 1.17, 95% CI: 0.95 to 1.44, P = 0.133), BMI (OR: 1.16, 95% CI: 0.93 to 1.45, P = 0.177), and Alcohol intake frequency (OR: 1.20, 95% CI: 0.97 to 1.40, P = 0.086).

### The causal relationship between T2DM and esophageal cancer in East Asian population is partially mediated by fasting insulin levels

To further explore the mechanism underlying the causal relationship between T2DM and digestive system tumors, we conducted a two-step MR study between T2DM, fasting insulin levels and fasting insulin levels and the aforementioned 5 types of tumors. Results are shown in **Table 5**. The results showed that T2DM decreased fasting insulin levels (OR = 0.97, 95% CI: 0.95 to 0.98, P< 0.001, **Supplementary Figures 3A–C**). Elevated fasting insulin levels increased the risk of esophageal cancer (OR = 10.35, 95% CI: 1.10 to 97.25, P = 0.041, **Supplementary Figure 4**), but no causal relationship was found with the other four types of tumors (**Table 5**).

**Table 5:** Results of MR analysis of the causal effect of T2DM on blood sugar, fasting blood sugar, and glycated hemoglobin A1c (HbA1c) levels, and the latter three on the 5 types of digestive system tumors.

outcome	exposure	method	number of snp	p value	or	95% CI	Q_pval	Egger intercept	pval	MR-PRESSO global test	Corrected Pvalue
Colorectal cancer || id:bbj-a-107	Blood sugar || id:bbj-a-10	Inverse variance weighted (multiplicative random effects)	15	0.792	1.075	(0.627,1.844)	8.12E-11	-0.005	0.928	NA	NA
Esophageal cancer || id:bbj-a-117	Blood sugar || id:bbj-a-10	Inverse variance weighted (multiplicative random effects)	15	0.63	0.677	(0.139,3.307)	1.08E-20	-0.196	0.25	<0.001	0.315
Gastric cancer || id:bbj-a-119	Blood sugar || id:bbj-a-10	Inverse variance weighted (multiplicative random effects)	15	0.365	0.818	(0.53,1.263)	2.46E-05	-0.031	0.512	NA	NA
Pancreatic cancer || id:bbj-a-140	Blood sugar || id:bbj-a-10	Inverse variance weighted	15	0.243	1.929	(0.641,5.809)	9.21E-02	-0.081	0.499	NA	NA
hepatocellular carcinoma || id:bbj-a-158	Blood sugar || id:bbj-a-10	Inverse variance weighted (multiplicative random effects)	15	0.834	1.089	(0.492,2.407)	2.91E-05	0.002	0.98	NA	NA
Esophageal cancer || id:bbj-a-117	Hemoglobin A1c || id:bbj-a-26	Inverse variance weighted	21	0.172	0.77	(0.529,1.120)	0.084503	0.051	0.309	NA	NA
Pancreatic cancer || id:bbj-a-140	Hemoglobin A1c || id:bbj-a-26	Inverse variance weighted	21	0.002	2.332	(1.371,3.965)	0.748545	-0.044	0.531	NA	NA
Colorectal cancer || id:bbj-a-107	Hemoglobin A1c || id:bbj-a-26	Inverse variance weighted (multiplicative random effects)	21	0.124	0.839	0.670,1.049)	6.24E-05	0.036	0.222	NA	NA
Gastric cancer || id:bbj-a-119	Hemoglobin A1c || id:bbj-a-26	Inverse variance weighted (multiplicative random effects)	21	0.044	0.801	(0.645,0.994)	0.001	0.014	0.642	NA	NA
hepatocellular carcinoma || id:bbj-a-158	Hemoglobin A1c || id:bbj-a-26	Inverse variance weighted (multiplicative random effects)	21	0.184	0.774	(0.531,1.129)	0.003	-0.014	0.789	NA	NA
Colorectal cancer || id:bbj-a-107	fasting insulin || id:ebi-a-GCST90002237	Inverse variance weighted	2	0.572	0.753	(0.281,2.015)	0.943	NA	NA	NA	NA
Esophageal cancer || id:bbj-a-117	fasting insulin || id:ebi-a-GCST90002237	Inverse variance weighted	2	0.041	10.351	(1.100,97.250)	0.925	NA	NA	NA	NA
Gastric cancer || id:bbj-a-119	fasting insulin || id:ebi-a-GCST90002237	Inverse variance weighted	2	0.666	0.798	(0.280,2.221)	0.952	NA	NA	NA	NA
hepatocellular carcinoma || id:bbj-a-158	fasting insulin || id:ebi-a-GCST90002237	Inverse variance weighted	2	0.928	0.917	(0.140,5.991)	0.731	NA	NA	NA	NA
Pancreatic cancer || id:bbj-a-140	fasting insulin || id:ebi-a-GCST90002237	Inverse variance weighted	2	0.811	1.736	(0.010,159.848)	0.236	NA	NA	NA	NA
Colorectal cancer || id:bbj-a-107	Fasting glucose || id:ebi-a-GCST90002231	Inverse variance weighted	10	0.434	1.18	(0.780,1.784)	0.103	-0.04	0.081	NA	NA
Esophageal cancer || id:bbj-a-117	Fasting glucose || id:ebi-a-GCST90002231	Inverse variance weighted (multiplicative random effects)	10	0.423	1.731	(0.450,6.631)	0	-0.045	0.588	NA	NA
Gastric cancer || id:bbj-a-119	Fasting glucose || id:ebi-a-GCST90002231	Inverse variance weighted	10	0.562	1.123	(0.758,1.665)	0.202	-0.007	0.76	NA	NA
Pancreatic cancer || id:bbj-a-140	Fasting glucose || id:ebi-a-GCST90002231	Inverse variance weighted	10	0.45	1.639	(0.455,5.906)	0.415	-0.124	0.122	NA	NA
hepatocellular carcinoma || id:bbj-a-158	Fasting glucose || id:ebi-a-GCST90002231	Inverse variance weighted	10	0.391	0.727	(0.351,1.506)	0.193	-0.053	0.216	NA	NA
Type 2 Diabetes || id:bbj-a-77	Fasting insulin || id:ebi-a-GCST90002237	Inverse variance weighted (multiplicative random effects)	55	0.001	0.962	(0.940,0.985)	1.20E-05	0.003	0.16	<0.001	1.58E-07
Type 2 diabetes || id:bbj-a-153	Fasting insulin || id:ebi-a-GCST90002237	Inverse variance weighted (multiplicative random effects)	59	0.005	0.967	(0.940,0.990)	3.36E-08	0.006	0.013	<0.001	5.09E-05
Type 2 diabetes || id:ebi-a-GCST010118	Fasting insulin || id:ebi-a-GCST90002237	Inverse variance weighted (multiplicative random effects)	130	0	0.967	(0.951,0.984)	3.13E-07	0.004	0.003	<0.001	2.85E-06
Type 2 Diabetes || id:bbj-a-77	Hemoglobin A1c || id:bbj-a-26	Inverse variance weighted (multiplicative random effects)	53	0	1.409	(1.362,1.458)	7.96E-07	-0.003	0.425	<0.001	1.62E-28
Type 2 diabetes || id:bbj-a-153	Hemoglobin A1c || id:bbj-a-26	Inverse variance weighted (multiplicative random effects)	57	0	1.419	(1.369,1.471)	2.91E-10	-0.009	0.018	<0.001	3.02E-27
Type 2 diabetes || id:ebi-a-GCST010118	Hemoglobin A1c || id:bbj-a-26	Inverse variance weighted (multiplicative random effects)	115	0	1.414	(1.379,1.449)	4.31E-06	-0.004	0.036	<0.001	3.81E-59
Gastric cancer || id:bbj-a-119	Two-hour glucose || id:ebi-a-GCST90002226	Inverse variance weighted (multiplicative random effects)	6	0.897	-0.125	(0.883,1.116)	0.002	-0.033	0.315	NA	NA

NA, Not Available.

### The causal relationship between T2DM and gastric cancer in the East Asian population is partially mediated by glycated hemoglobin A1c levels

Further, we conducted a two-step MR study between T2DM with blood sugar, fasting blood sugar and glycated hemoglobin A1c (HbA1c) levels, and the latter three with the aforementioned 5 types of tumors. Results were shown in **Table 5**. The results showed that T2DM increased the levels of HbA1c (OR=1.41, 95% CI: 1.39 to 1.44, P<0.001, **Supplementary Figure 5**) and an increase in the levels of HbA1c could reduce the risk of gastric cancer (OR=0.80, 95% CI: 0.65 to 0.99, P=0.044, **Supplementary Figures 3D–G**). However, no causal relationship was found between blood sugar and fasting blood sugar with gastric cancer (**Table 5**). It should be noted that heterogeneity existed for the MR analysis between HbA1c and gastric cancer as P<0.05 by Cochran’s Q test. However, the leave-one-out method confirmed the robustness of the result (P=0.044, **Supplementary Figure 3G**).

### The causal relationship between T2DM and pancreatic cancer in East Asian population is partially mediated by the HbA1c levels

Our results showed that elevated HbA1c levels increased the risk of pancreatic cancer (OR=2.33, 95% CI: 1.37 to 3.97, P=0.002, **Supplementary Figures 3H–K**), but no causal relationship was found between blood glucose and fasting blood glucose with pancreatic cancer (**Table 5**). Considering that T2DM increases HbA1c levels (OR=1.41, P<0.001, **Supplementary Figure 5**) and T2DM has a significant direct causal relationship with pancreatic cancer (OR=1.92, 95% CI: 1.47 to 2.50, P<0.001, **Figure 2E**), the causal relationship between T2DM and pancreatic cancer is partially mediated by HbA1c levels.

In the sensitivity analyses, the three MR-PRESSO global tests failed to detect any horizontal pleiotropy (p = 0.166, 0.125, 0.133, with instruments of exposure from ebi-a-GCST010118, bbj-a-77,bbj-a-153, respectively) or outliers, while all three TSMR analyses using the MR Egger method with pancreatic cancer as the outcome have detected significant Egger intercepts (-0.049, -0.048, -0.045; p = 0.001, 0.041, 0.041, respectively), indicating some evidence of pleiotropy and the pleiotropy not deriving from individual outliers. Therefore, MR-Egger could be more suitable for detecting and correcting for the bias due to directional pleiotropy. However, causal estimates from the MR-Egger method may be biased and have inflated Type 1 error rates in practice (23).

## Discussion

We analyzed a series of phenotypic GWAS data through Mendelian randomization method, and the results showed that T2DM had a significant causal effect on esophageal cancer, colorectal cancer and hepatocellular carcinoma, and a discrepancy existed between European and East Asian populations. In the East Asian population, T2DM has a significant causal effect on esophageal cancer, gastric cancer, colorectal cancer and hepatocellular carcinoma, and the causal relationships between T2DM with esophageal cancer and gastric cancer are partly mediated by fasting insulin and Hb1Ac levels, respectively. Direct causal relationship between T2DM and pancreatic cancer was confirmed, and meanwhile, two-step MR suggests that T2DM increases the risk of pancreatic cancer by increasing Hb1Ac levels.

### The association between T2DM and hepatocellular carcinoma

The findings of a study in 2020 confirmed that patients with T2DM carry a higher risk of developing hepatocellular carcinoma (HCC), but the risk may depend on the underlying liver disease etiology (21). When compared with nondiabetics, the strongest correlation was seen among patients with non-alcoholic steatohepatitis (NASH), and the increased risk was confirmed after adjusting for other known risk factors for HCC. Diabetics with NASH, cryptogenic cirrhosis, HCV, and alcoholic liver disease showed a higher risk of HCC than nondiabetics, whereas T2DM did not increase the risk of HCC among patients with HBV or primary biliary cholangitis (PBC) (21). Previous studies have provided evidence that chronic HCV infection may induce insulin resistance (18) and sustained virological response (SVR) reduces the risk of impaired fasting glucose (IFG) and/or T2DM development in patients with chronic HCV (19, 20). Therefore, chronic HCV infection and cirrhosis are 2 potential confounders influencing the incidence risk of HCC. However, we failed to figure out the prevalence of chronic liver disease in the cohorts of HCC (27). We performed an IVW-based MVMR to confirm the direct effect of T2DM on HCC after adjusting for chronic HCV infection and cirrhosis, respectively. After adjusting for chronic HCV infection (OR: 0.99, 95% CI: 0.89 to 1.09, P = 0.783) and cirrhosis (OR: 0.95, 95% CI: 0.88 to 1.04, P = 0.296), the effect of T2DM on HCC was not significant (**Supplementary Figure 2**, **Supplementary Table 7**).

### The association between T2DM with gastric and colorectal cancer

Our study found a significant causal relationship between T2DM and a reduced incidence of esophageal cancer, gastric cancer and colorectal cancer in East Asian population. However, previous observational cohort studies have suggested an increased risk of cancer associated with diabetes. Tsilidis et al. summarized data from observational studies on the incidence and mortality of cancers in individuals with T2DM and found an increased risk of several cancers, including hepatocellular carcinoma, pancreatic cancer and gastrointestinal cancer, was associated with T2DM (3). A meta-analysis by Noto et al. found an increased incidence of all cancer types associated with diabetes, with significantly higher cancer incidence in Asian male compared to non-Asian male (28). Prospective cohort studies in Japan showed that an increased risk of colorectal, liver and pancreatic cancer was associated with diabetes (4). A cohort study based on the United Kingdom (UK) Clinical Practice Research Datalink (CPRD) (1988–2012) found an increased incidence of liver, colorectal and pancreatic cancer in patients with diabetes compared to those without (5).

The potential mechanisms underlying the link between T2DM and cancer include hyperglycemia, insulin resistance, high insulin levels and increased insulin-like growth factor I (IGF-1) levels, etc (29). Smoking, male gender and low-density lipoprotein cholesterol (LDL-C)<100mg/dl were found to be risk factors for diabetic patients to develop cancer, while body mass index (BMI), alcohol consumption and HbA1C levels were not associated with cancer occurrence in diabetic population (30). A prospective case-control study in Korea showed that higher blood glucose levels, lower high-density lipoprotein cholesterol (HDL-C) and homeostasis model assessment of insulin resistance (HOMA-IR) levels were associated with the risk of early stage gastric cancer (31). A study in Sweden found that high blood glucose levels were associated with male colon cancer risk (32). In contrast, we found that HbA1C levels were inversely associated with gastric cancer risk in East Asian population. Although HbA1c levels are associated with the glycemic control status of diabetic patients, we were unable to determine the causal relationship between blood sugar levels (including overall blood sugar levels, fasting blood sugar levels and 2-hour blood sugar levels) and gastric cancer. Some studies have shown a negative correlation between gastric mucosal innervation density (MID) with fasting blood sugar levels and glycated hemoglobin levels (33), besides, vagotomy inhibits gastric cancer development by inhibiting tumor cell proliferation through suppressing WNT signaling pathway (34, 35), which may help explain our results.

### The association between T2DM and esophageal cancer

Our research suggests that in the European population, individuals at high risk of T2DM are at increased risk for esophageal cancer, whereas in the East Asian population, individuals at high risk of T2DM may have a lower risk for esophageal cancer. This protective effect is partially related to the decrease in fasting insulin levels caused by T2DM. Previous studies have shown conflicting results regarding the effect of T2DM on the incidence of esophageal cancer. Mendelian randomization analysis mainly based on European populations showed that the genetic susceptibility of T2DM is negatively associated with the incidence of esophageal cancer (36).

A cohort study based on the UK CPRD (1988–2012) found that the incidence of esophageal cancer was lower in diabetic patients than that in non-diabetic patients (5). Squamous cell carcinoma is the most common histological type of esophageal cancer worldwide, including in China, however, adenocarcinoma is the dominant histological type of esophageal cancer in European and American populations (37). A meta-analysis found a significant correlation between T2DM and esophageal cancer in European and American subjects, while no correlation was found in Asian subjects (38). Another meta-analysis based on American population found a significant correlation between T2DM and the risk of esophageal adenocarcinoma (EADC) (39).

Research from Finland showed that high levels of fasting blood glucose and fasting insulin were associated with an increased risk of liver cancer (40), colorectal cancer (41), pancreatic cancer (42) and colon adenoma (41) in certain populations. A meta-analysis in 2015 showed a significant correlation between high insulin levels and colon adenoma, but the correlation was weak in Asian populations (43). A Mendelian randomization study based on European GWAS data found that fasting insulin levels, rather than high blood glucose, was causally related to the risk of colon cancer (44). Currently, research on the relationship between fasting insulin levels and the risk of esophageal cancer is lacking. Our research found that high fasting insulin levels increased the risk of esophageal cancer, but there was no significant causal relationship between fasting insulin levels and the risks of liver, gastric, colorectal or pancreatic cancer. The characteristics of diabetes in the East Asian population differ from those in the European and American populations. The East Asian population has a lower average BMI, a greater tendency towards body fat and visceral fat, and a younger age of onset and mainly presents with insulin resistance and early-stage beta cell dysfunction (1). The earlier onset and lower levels of fasting insulin levels in the East Asian population (1) may be the reasons for the protective effect of T2DM against esophageal cancer.

### The association between T2DM and pancreatic cancer

Previous observational studies showed that T2DM was associated with pancreatic cancer (3–5, 28). We have validated this relationship through TSMR, and what’s more, we found that T2DM may cause elevated HbA1c levels in the East Asian population and high HbA1c levels increased the risk of pancreatic cancer. The meta-analysis conducted by Hope et al. showed that elevated HbA1c levels were associated with an increased risk of colorectal and pancreatic cancer, but not with gastrointestinal malignancies (45). Studies of the British population showed that elevated HbA1c levels were associated with an increased risk of colon, liver, esophageal and pancreatic cancer (46). Results of our study are consistent with these observational studies.

### Limitations

This study included four phenotypes of T2DM and its manifestations, as well as five types of digestive system cancers. We conducted a meta-analysis of multiple GWAS datasets for the same phenotype. The main advantage of a TSMR study design is reducing the impacts of confounding factors and reverse causality. However, our study still has certain limitations: currently, there are few instrumental variables for digestive system cancers in East Asian populations. Our GWAS datasets source for digestive system cancers in East Asian populations mainly rely on a single study (27), thus requiring more data sources to confirm our results. In addition, the numbers of SNPs instruments in several analyses were small, and sensitivity analyses could not be performed for three MR analyses, which may have affected the reliability of the results. Third, East Asian regions have a higher prevalence of *Helicobacter pylori*, liver fluke, and HBV and HCV infections, hot beverage consumption and biliary cyst development, which might have increased the risk of gastrointestinal cancers (25). Instrumental variables may only account for a small portion of the observed variability, and further research is needed to fully understand the complex changes in the gastrointestinal carcinogenesis. Fourth, though MR-PRESSO global test in all three TSMR analyses with pancreatic cancer as the outcome were insignificant, the results of MR Egger intercept test indicated some evidence of pleiotropy. And causal estimates from the MR-Egger method may be biased and have inflated Type 1 error rates in practice. Finally, since detailed baseline characteristics of study subjects (e.g. tumor markers, tumor stage, etc.) were not provided in the GWAS studies we used, we could not further investigate the effect of T2DM on different subgroups of the populations and also could not exclude the possibility that survivorship bias exists in our study.

### Conclusion

Our findings suggest that T2DM can reduce the incidence of esophageal cancer, gastric cancer, colorectal cancer and hepatocellular carcinoma in East Asian population. The causal relationships between T2DM with esophageal cancer and gastric cancer are partially attributed to the reduction in fasting insulin levels and the elevation in glycated hemoglobin levels, respectively. T2DM indirectly increases the risk of pancreatic cancer by increasing glycated hemoglobin levels.

## Data availability statement

The original contributions presented in the study are included in the article/**Supplementary Material**. Further inquiries can be directed to the corresponding authors.

## Ethics statement

Ethical approval was not required for the study involving humans in accordance with the local legislation and institutional requirements. Written informed consent to participate in this study was not required from the participants or the participants’ legal guardians/next of kin in accordance with the national legislation and the institutional requirements.

## Author contributions

NA: Conceptualization, Writing – original draft, Writing – review & editing. YZ: Investigation, Methodology, Visualization, Writing – original draft. ZS: Project administration, Validation, Writing – review & editing. ZX: Resources, Supervision, Writing – review & editing. XL: Project administration, Resources, Supervision, Writing – review & editing.

## Supplementary material

Supplementary Figure 1 The MR results of T2DM on colorectal cancer and pancreatic cancer in European population. **(A)** Meta-analysis of the MR effects of T2DM on colorectal cancer in European population (estimated by IVW method). **(B)** Meta-analysis of the MR effects of T2DM on pancreatic cancer in European population (estimated by IVW method).

Supplementary Figure 2 The MR results of CHC, Cirrhosis, and T2DM on HCC in East Asian population.

Supplementary Figure 3 The MR results of fasting insulin and Hb1Ac levels on esophageal, gastric and pancreatic cancer in East Asian population. **(C)** Scatter plot, forest plot and volcano plot of MR analysis of the relationship between fasting insulin levels and esophageal cancer, respectively. **(D–G)** Scatter plot, forest plot, volcano plot and leave-one-out plot of MR analysis of the relationship between Hb1Ac levels and gastric cancer, respectively. **(H–K)** Scatter plot, forest plot, volcano plot and leave-one-out plot of MR analysis of the relationship between Hb1Ac levels and pancreatic cancer, respectively.

Supplementary Figure 4 The MR results of T2DM on fasting insulin levels in East Asian population. **(A)** Meta-analysis of the MR effects of T2DM on fasting insulin levels in East Asian population (estimated by IVW method). **(B, C)** Scatter plot and Leave-one-out plot of MR analysis of the relationship between T2DM and fasting insulin levels, with GWAS ID of bbj-a-77 as the instrumental variable for T2DM, respectively. **(D, E)** Scatter plot and Leave-one-out plot of MR analysis of the relationship between T2DM and fasting insulin levels, with GWAS ID of bbj-a-153 as the instrumental variable for T2DM, respectively. **(F, G)** Scatter plot and Leave-one-out plot of MR analysis of the relationship between T2DM and fasting insulin levels, with GWAS ID of ebi-a-GCST010118 as the instrumental variable for T2DM, respectively.

Supplementary Figure 5 The MR results of T2DM on Hb1Ac levels in East Asian population. **(A)** Meta-analysis of the MR effects of T2DM on Hb1Ac levels in East Asian population (estimated by IVW method). **(B, C)** Scatter plot and Leave-one-out plot of MR analysis of the relationship between T2DM and Hb1Ac levels, with GWAS ID of bbj-a-77 as the instrumental variable for T2DM, respectively. **(D, E)** Scatter plot and Leave-one-out plot of MR analysis of the relationship between T2DM and Hb1Ac levels, with GWAS ID of bbj-a-153 as the instrumental variable for T2DM, respectively. **(F, G)** Scatter plot and Leave-one-out plot of MR analysis of the relationship between T2DM and Hb1Ac levels, with GWAS ID of ebi-a-GCST010118 as the instrumental variable for T2DM, respectively.

Supplemental File 1 The plot of MR result of T2DM on colorectal cancer in East Asian.

Supplemental File 2 The plot of MR result of T2DM on esophageal cancer in East Asian.

Supplemental File 3 The plot of MR result of T2DM on gastric cancer in East Asian.

Supplemental File 4 The plot of MR result of T2DM on hepatocellular carcinoma in East Asian.

Supplemental File 5 The plot of MR result of T2DM on pancreatic cancer in East Asian.
