# Supplementary figures and images for: T2DM may exert a protective effect against digestive system tumors in East Asian populations: a Mendelian randomization analysis
Source: Front Oncol. 2024 Jun 14;14:1327154. doi: 10.3389/fonc.2024.1327154 (PMC11211363; doi:10.3389/fonc.2024.1327154)

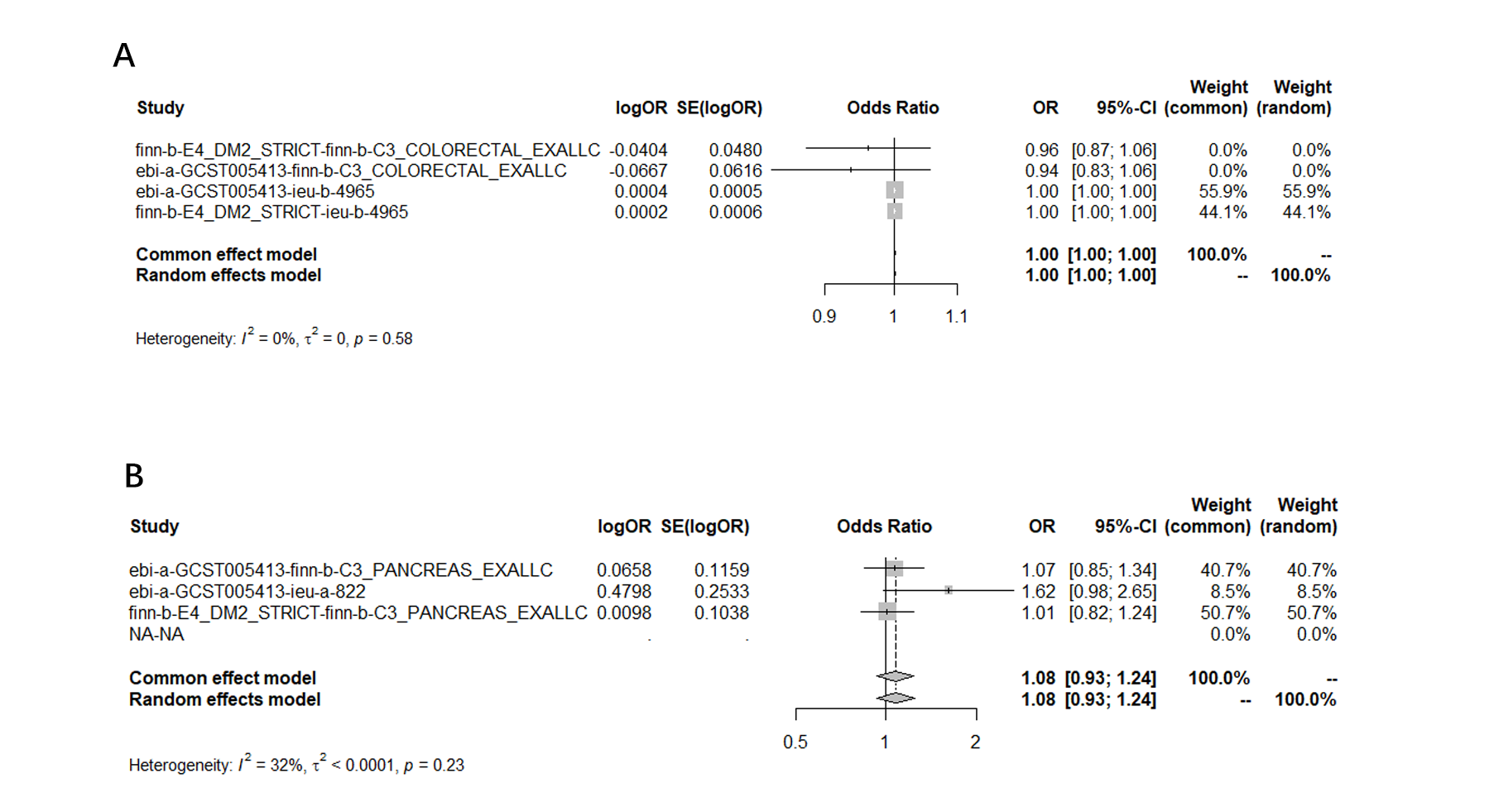

Supplement: Supplementary Figure 1 — The MR results of T2DM on colorectal cancer and pancreatic cancer in European population. (A) Meta-analysis of the MR effects of T2DM on colorectal cancer in European population (estimated by IVW method). (B) Meta-analysis of the MR effects of T2DM on pancreatic cancer in European population (estimated by IVW method). [file Image_1.tif]

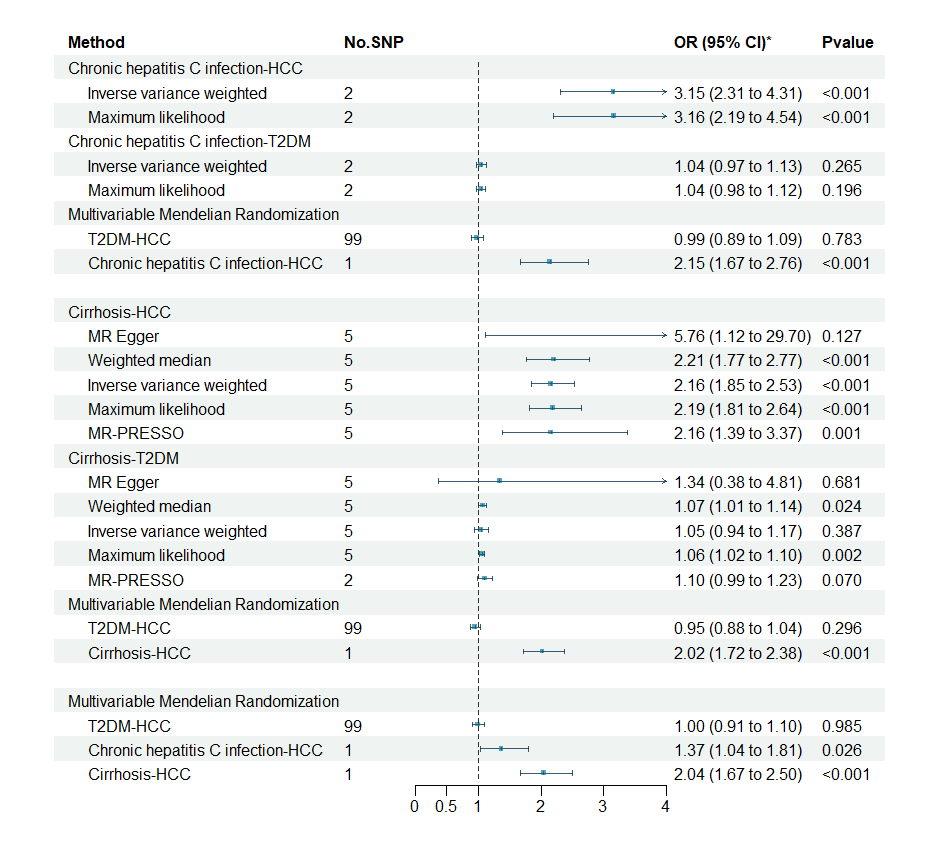

Supplement: Supplementary Figure 2 — The MR results of CHC, Cirrhosis, and T2DM on HCC in East Asian population. [file Image_2.tiff]

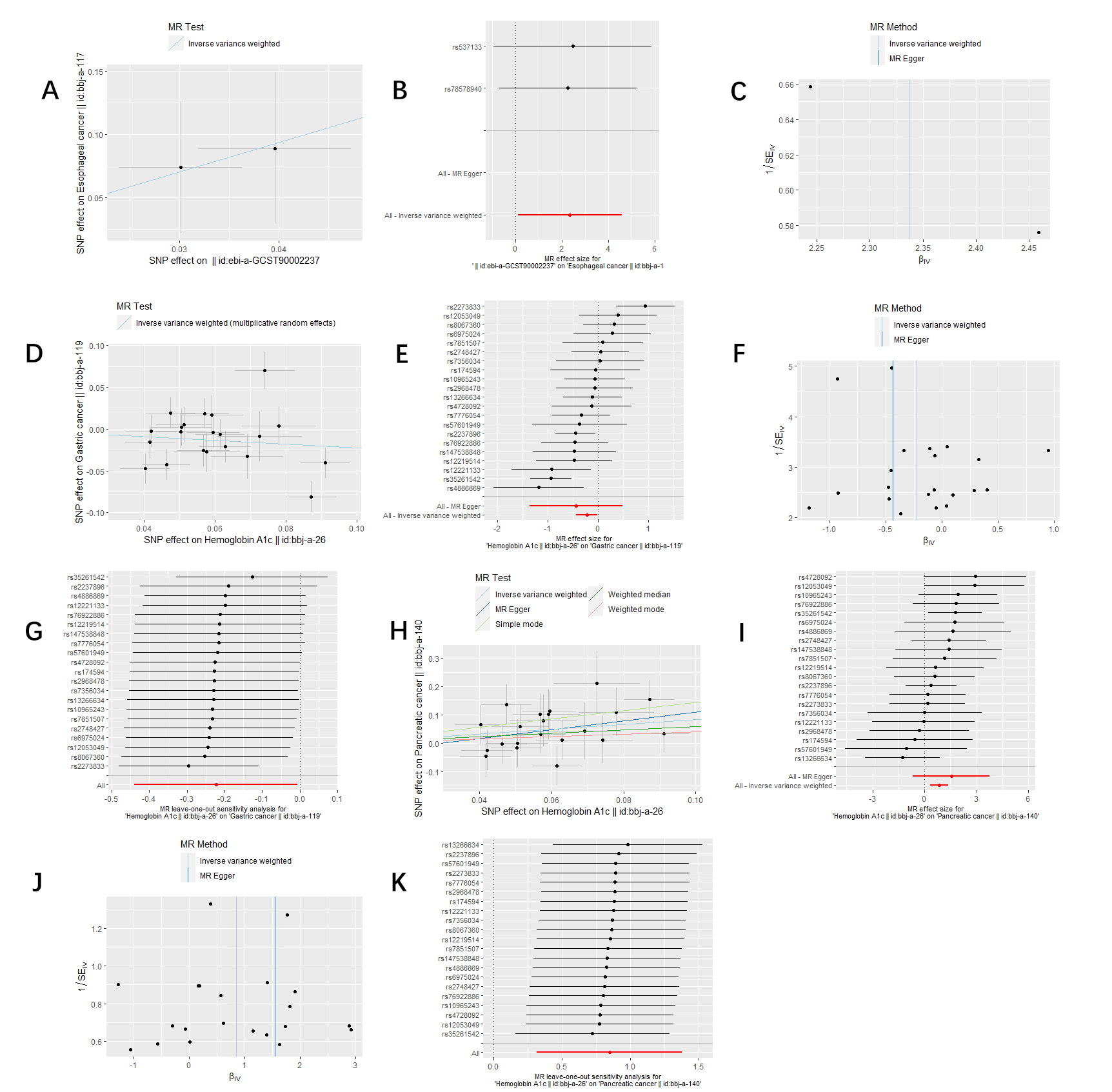

Supplement: Supplementary Figure 3 — The MR results of fasting insulin and Hb1Ac levels on esophageal, gastric and pancreatic cancer in East Asian population. (C) Scatter plot, forest plot and volcano plot of MR analysis of the relationship between fasting insulin levels and esophageal cancer, respectively. (D–G) Scatter plot, forest plot, volcano plot and leave-one-out plot of MR analysis of the relationship between Hb1Ac levels and gastric cancer, respectively. (H–K) Scatter plot, forest plot, volcano plot and leave-one-out plot of MR analysis of the relationship between Hb1Ac levels and pancreatic cancer, respectively. [file Image_3.tif]

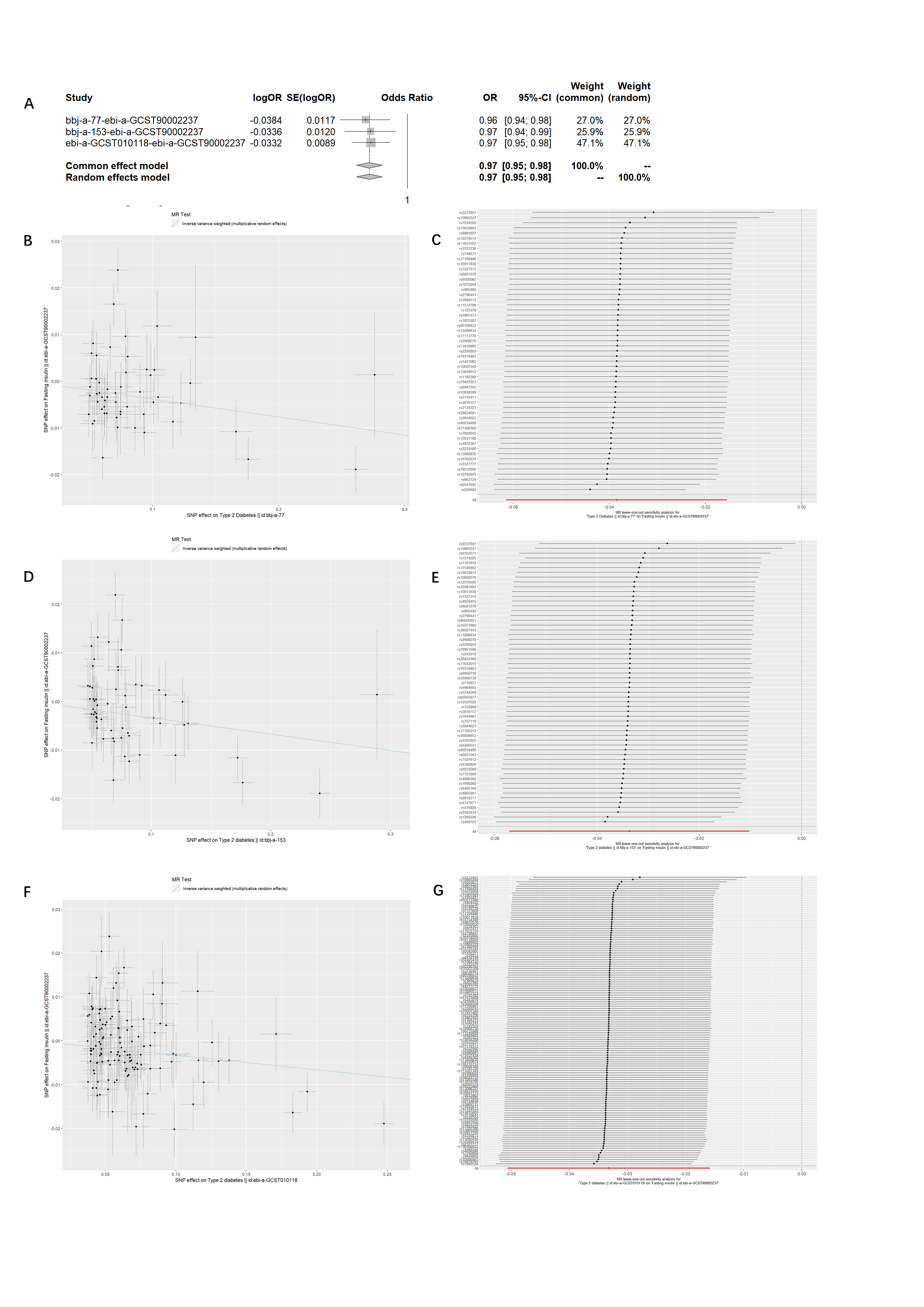

Supplement: Supplementary Figure 4 — The MR results of T2DM on fasting insulin levels in East Asian population. (A) Meta-analysis of the MR effects of T2DM on fasting insulin levels in East Asian population (estimated by IVW method). (B, C) Scatter plot and Leave-one-out plot of MR analysis of the relationship between T2DM and fasting insulin levels, with GWAS ID of bbj-a-77 as the instrumental variable for T2DM, respectively. (D, E) Scatter plot and Leave-one-out plot of MR analysis of the relationship between T2DM and fasting insulin levels, with GWAS ID of bbj-a-153 as the instrumental variable for T2DM, respectively. (F, G) Scatter plot and Leave-one-out plot of MR analysis of the relationship between T2DM and fasting insulin levels, with GWAS ID of ebi-a-GCST010118 as the instrumental variable for T2DM, respectively. [file Image_4.tif]

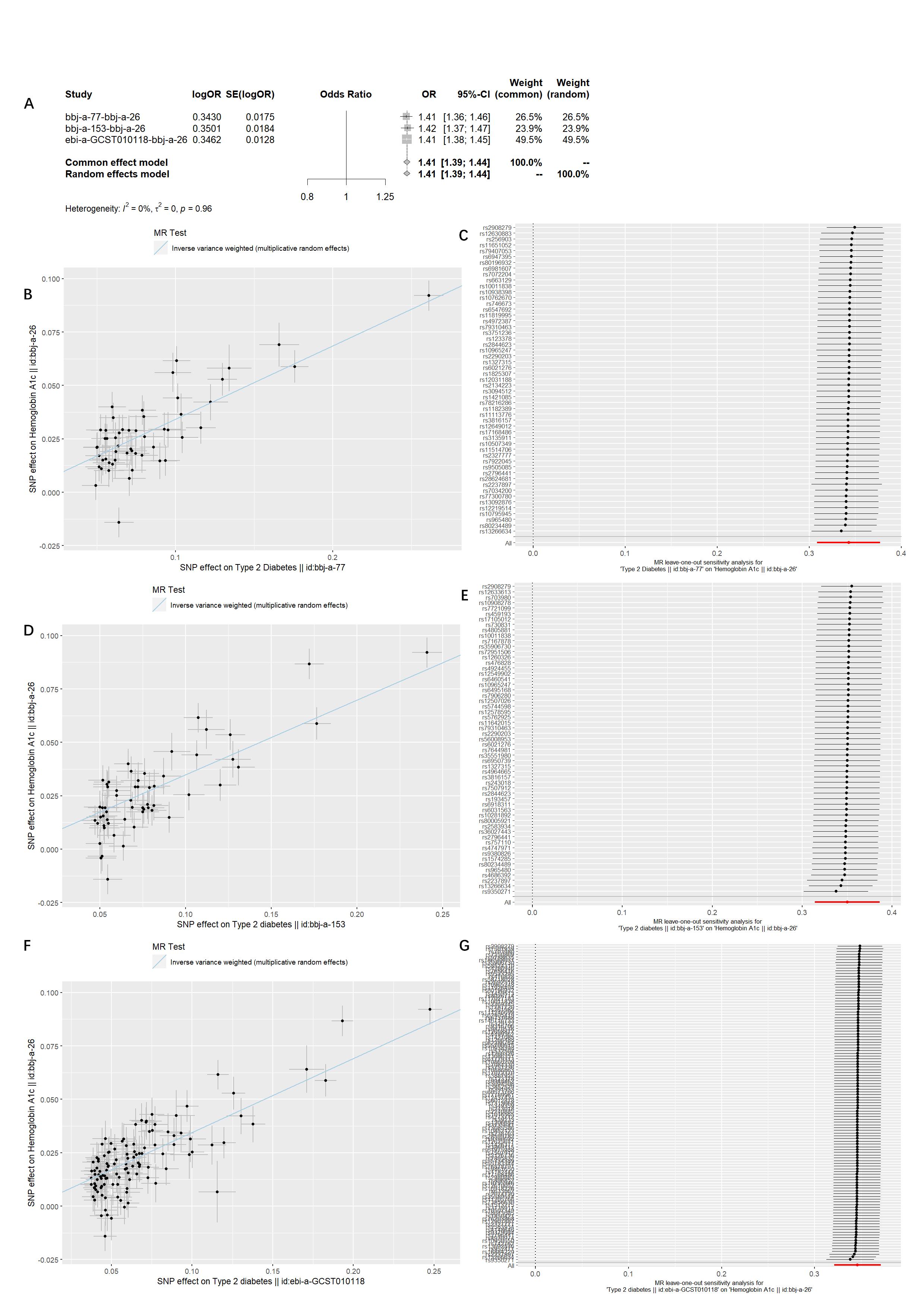

Supplement: Supplementary Figure 5 — The MR results of T2DM on Hb1Ac levels in East Asian population. (A) Meta-analysis of the MR effects of T2DM on Hb1Ac levels in East Asian population (estimated by IVW method). (B, C) Scatter plot and Leave-one-out plot of MR analysis of the relationship between T2DM and Hb1Ac levels, with GWAS ID of bbj-a-77 as the instrumental variable for T2DM, respectively. (D, E) Scatter plot and Leave-one-out plot of MR analysis of the relationship between T2DM and Hb1Ac levels, with GWAS ID of bbj-a-153 as the instrumental variable for T2DM, respectively. (F, G) Scatter plot and Leave-one-out plot of MR analysis of the relationship between T2DM and Hb1Ac levels, with GWAS ID of ebi-a-GCST010118 as the instrumental variable for T2DM, respectively. [file Image_5.tif]
