# Supplementary material for: T2DM may exert a protective effect against digestive system tumors in East Asian populations: a Mendelian randomization analysis
Source: Front Oncol. 2024 Jun 14;14:1327154. doi: 10.3389/fonc.2024.1327154 (PMC11211363; doi:10.3389/fonc.2024.1327154)
Supplement: Supplemental File 1 — The plot of MR result of T2DM on colorectal cancer in East Asian. [file DataSheet_1.pdf]

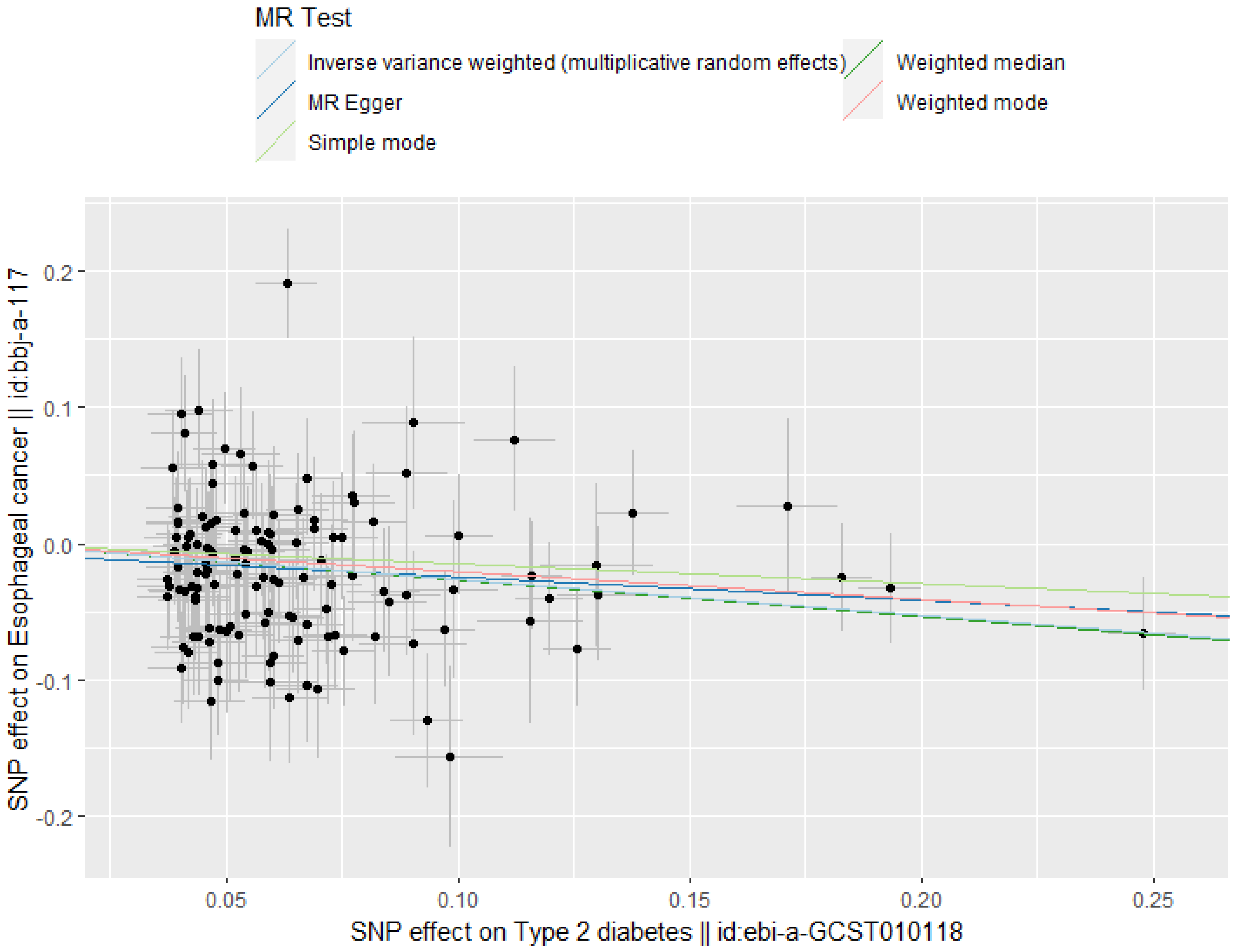

MR Test
Inverse variance weighted (multiplicative random effects)
MR Egger
Simple mode
Weighted median
Weighted mode
SNP effect on Esophageal cancer || id:bbj-a-117
0.2
0.1
0.0
-0.1
-0.2
0.05
0.10
0.15
0.20
0.25
SNP effect on Type 2 diabetes || id:ebi-a-GCST010118

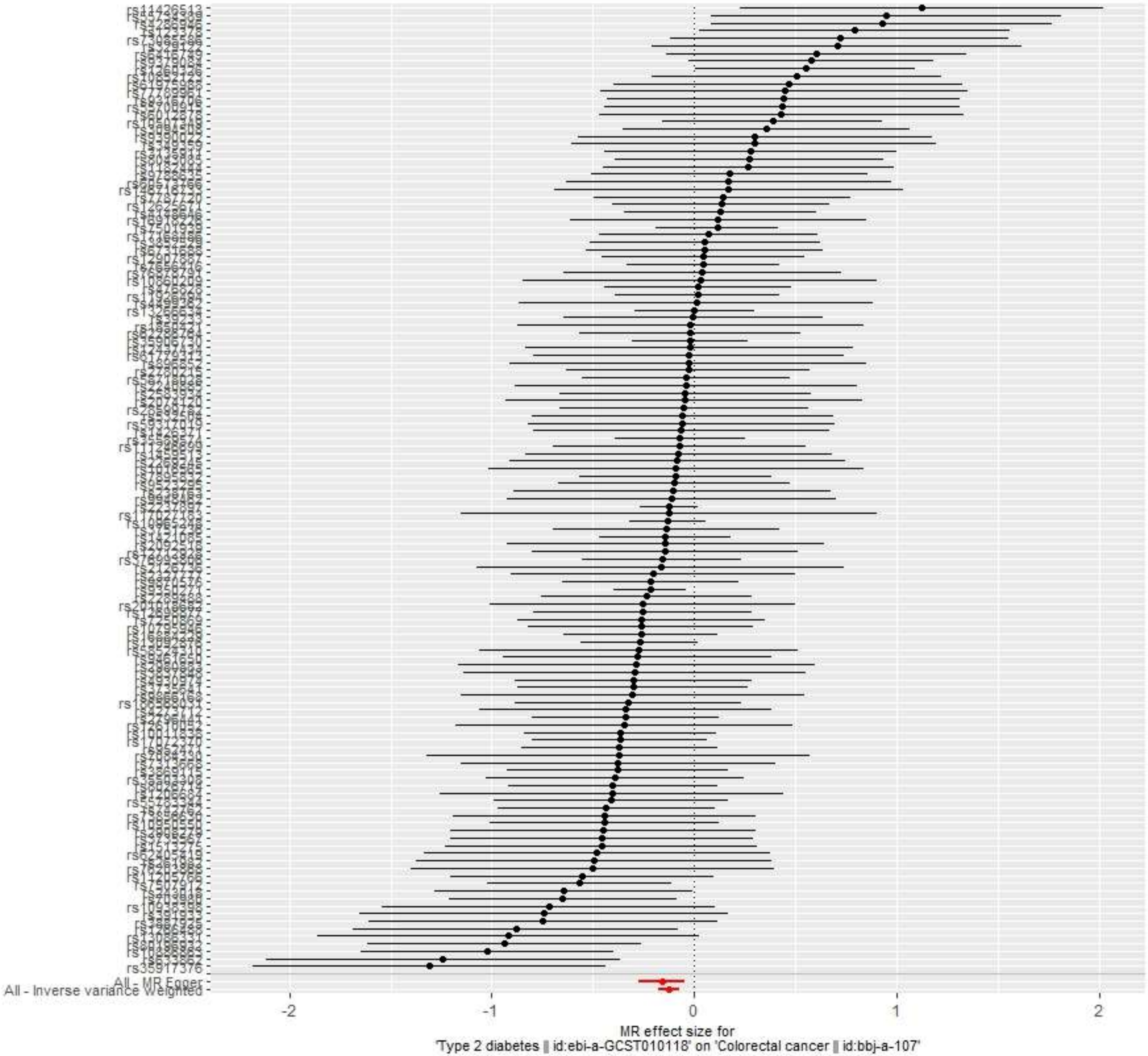

rs11426513
rs35917376
All - MR Egger
All - Inverse variance weighted
-2
-1
0
1
2
MR effect size for
'Type 2 diabetes || id:ebi-a-GCST010118' on 'Colorectal cancer || id:bbj-a-107'

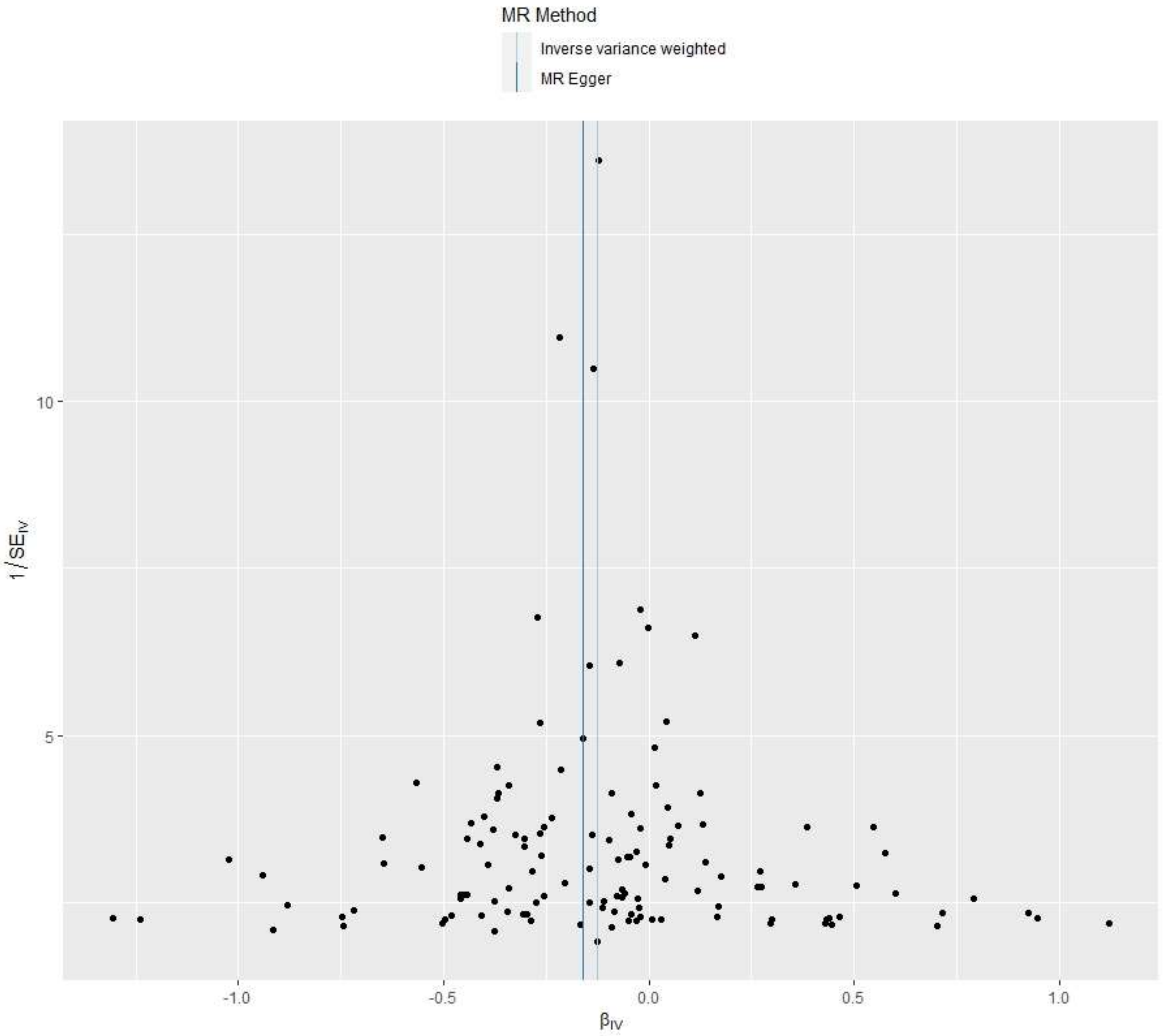
MR Method
Inverse variance weighted
MR Egger
10
5
-1.0
-0.5
0.0
0.5
1.0
$1/SE_{IV}$
β_{IV}

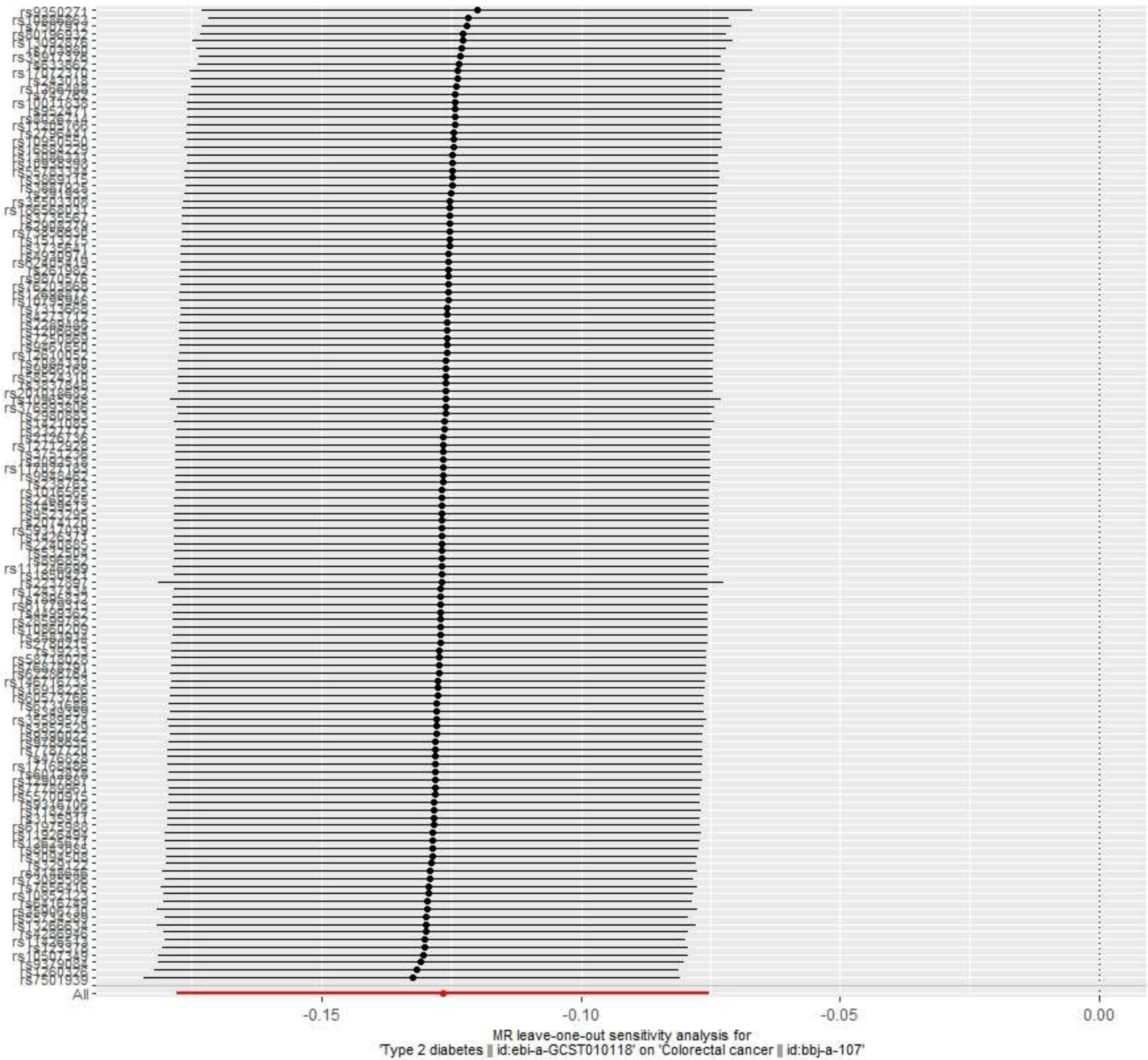
rs9350271
rs7501939
All
-0.15
-0.10
-0.05
0.00
MR leave-one-out sensitivity analysis for
'Type 2 diabetes || id:ebi-a-GCST010118' on 'Colorectal cancer || id:bbj-a-107'

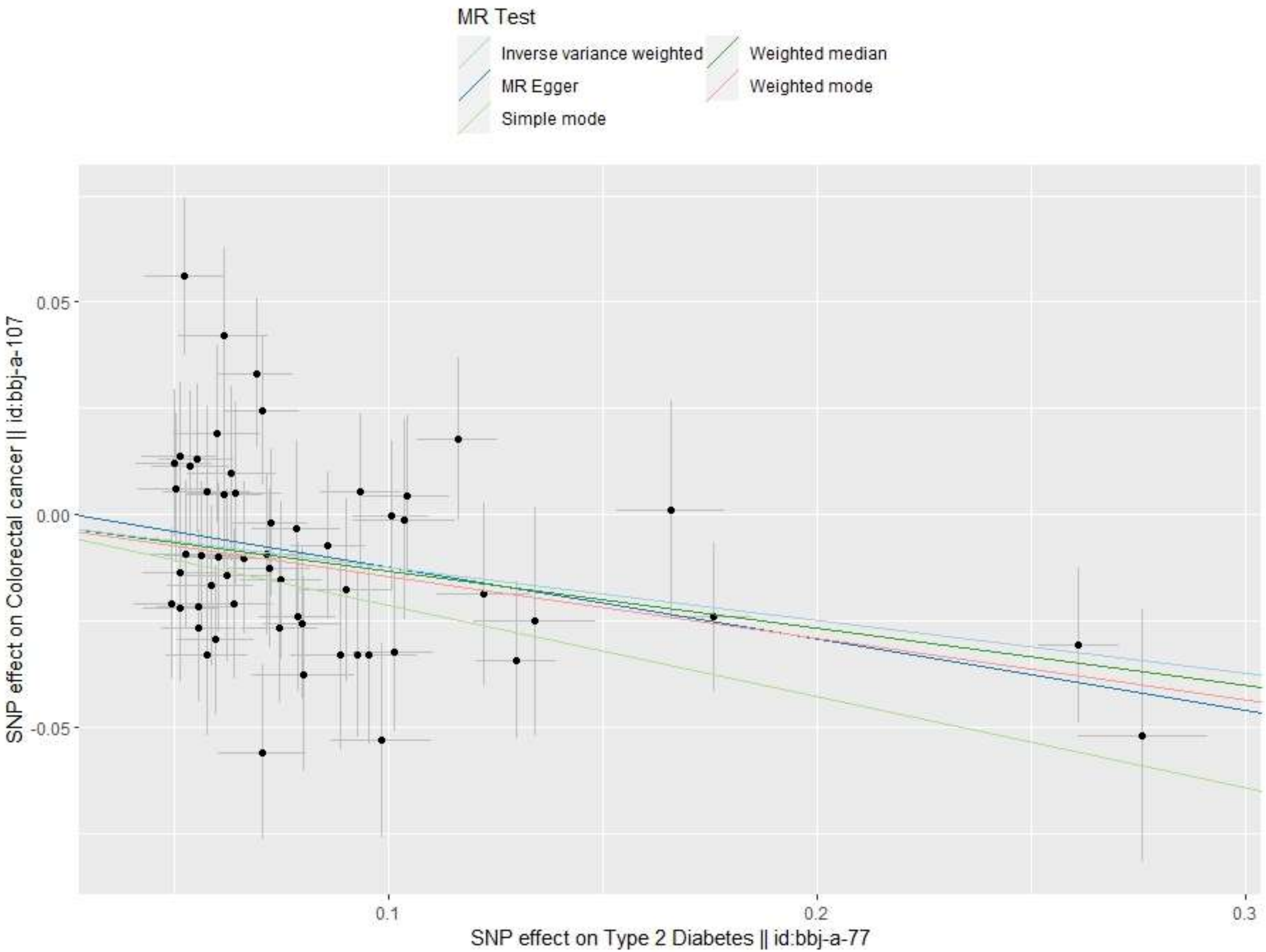

MR Test
Inverse variance weighted
MR Egger
Simple mode
Weighted median
Weighted mode
0.05
0.00
-0.05
0.1
0.2
0.3
SNP effect on Colorectal cancer || id:bbj-a-107
SNP effect on Type 2 Diabetes || id:bbj-a-77

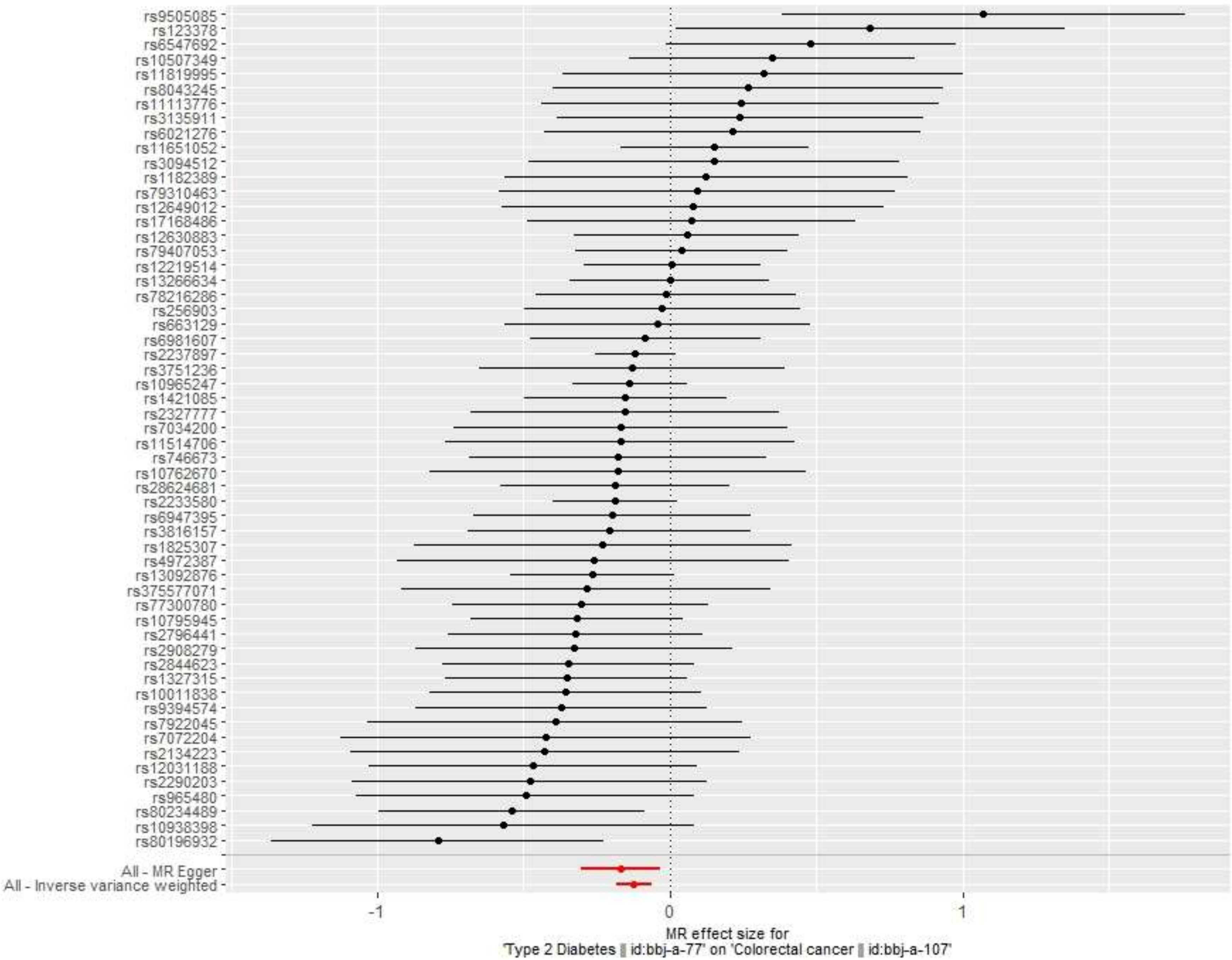

rs9505085
rs123378
rs6547692
rs10507349
rs11819995
rs8043245
rs11113776
rs3135911
rs6021276
rs11651052
rs3094512
rs1182389
rs79310463
rs12649012
rs17168486
rs12630883
rs79407053
rs12219514
rs13266634
rs78216286
rs256903
rs663129
rs6981607
rs2237897
rs3751236
rs10965247
rs1421085
rs2327777
rs7034200
rs11514706
rs746673
rs10762670
rs28624681
rs2233580
rs6947395
rs3816157
rs1825307
rs4972387
rs13092876
rs375577071
rs77300780
rs10795945
rs2796441
rs2908279
rs2844623
rs1327315
rs10011838
rs9394574
rs7922045
rs7072204
rs2134223
rs12031188
rs2290203
rs965480
rs80234489
rs10938398
rs80196932
All - MR Egger
All - Inverse variance weighted
-1
0
1
MR effect size for
'Type 2 Diabetes || id:bbj-a-77' on 'Colorectal cancer || id:bbj-a-107'

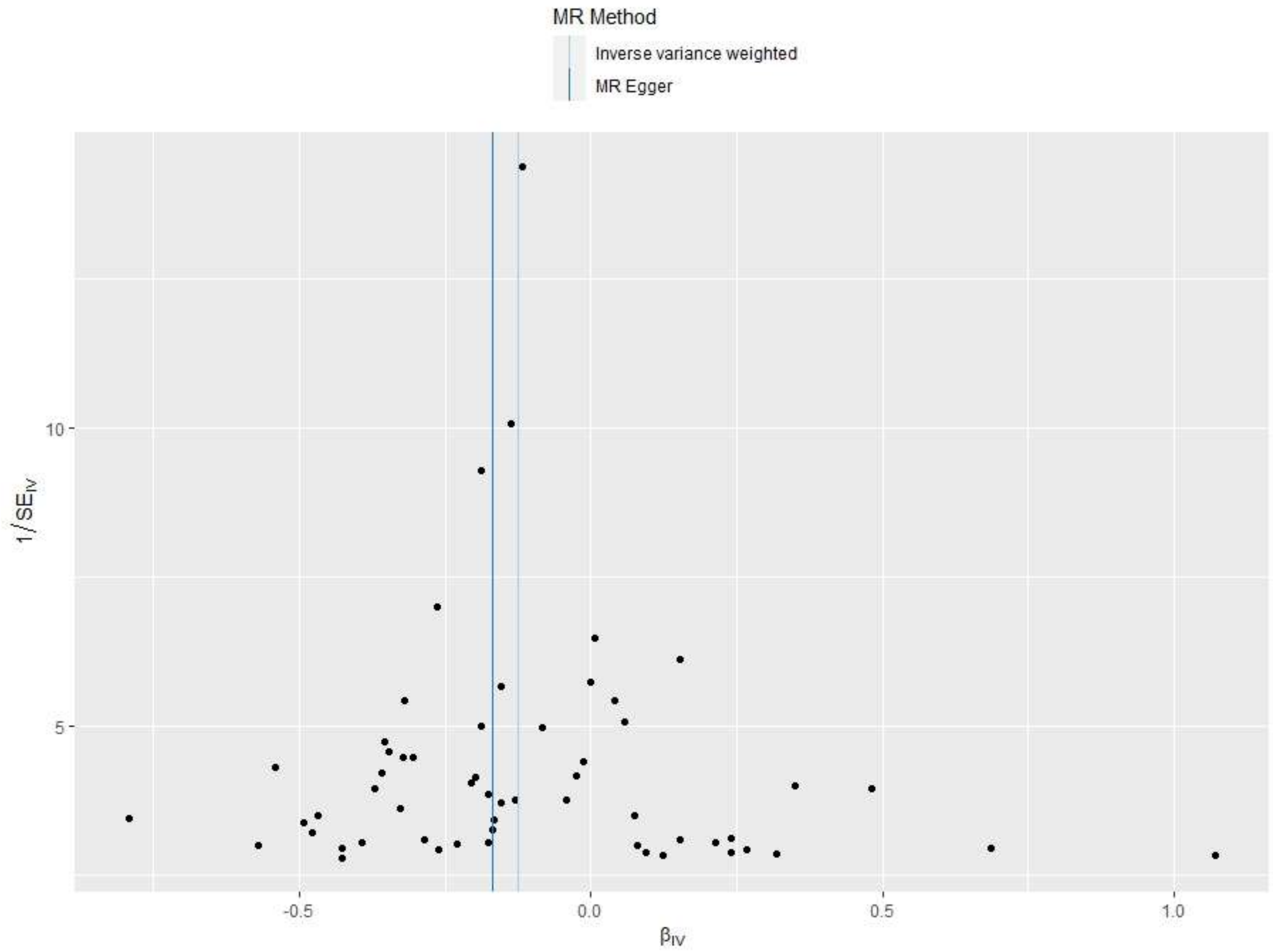

MR Method
Inverse variance weighted
MR Egger
10
5
1/SE_IV
-0.5
0.0
0.5
1.0
β_IV

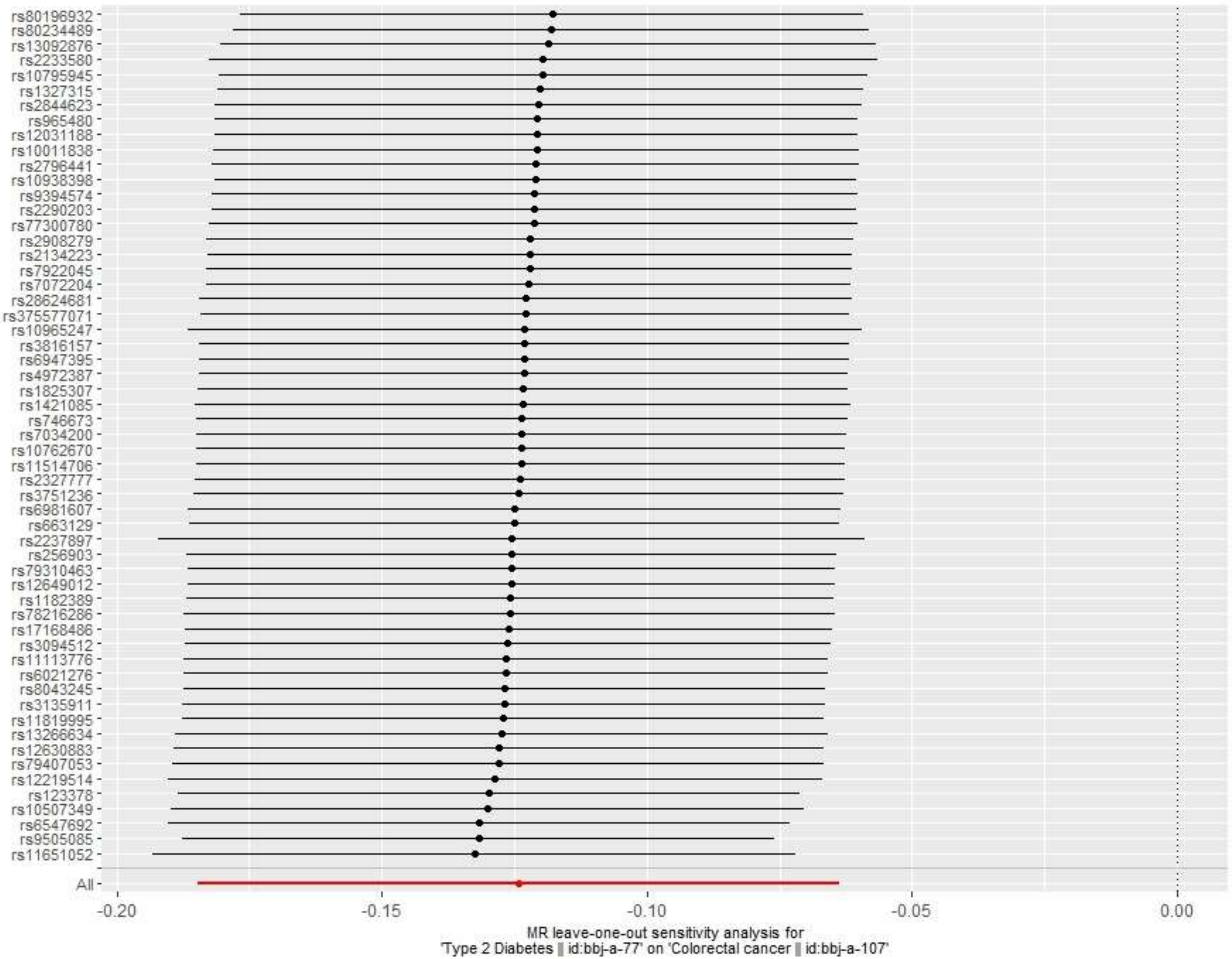

rs80196932
rs80234489
rs13092876
rs2233580
rs10795945
rs1327315
rs2844623
rs965480
rs12031188
rs10011838
rs2796441
rs10938398
rs9394574
rs2290203
rs77300780
rs2908279
rs2134223
rs7922045
rs7072204
rs28624681
rs375577071
rs10965247
rs3816157
rs6947395
rs4972387
rs1825307
rs1421085
rs746673
rs7034200
rs10762670
rs11514706
rs2327777
rs3751236
rs6981607
rs663129
rs2237897
rs256903
rs79310463
rs12649012
rs1182389
rs78216286
rs17168486
rs3094512
rs11113776
rs6021276
rs8043245
rs3135911
rs11819995
rs13266634
rs12630883
rs79407053
rs12219514
rs123378
rs10507349
rs6547692
rs9505085
rs11651052
All
-0.20
-0.15
-0.10
-0.05
0.00
MR leave-one-out sensitivity analysis for
'Type 2 Diabetes || id:bbj-a-77' on 'Colorectal cancer || id:bbj-a-107'

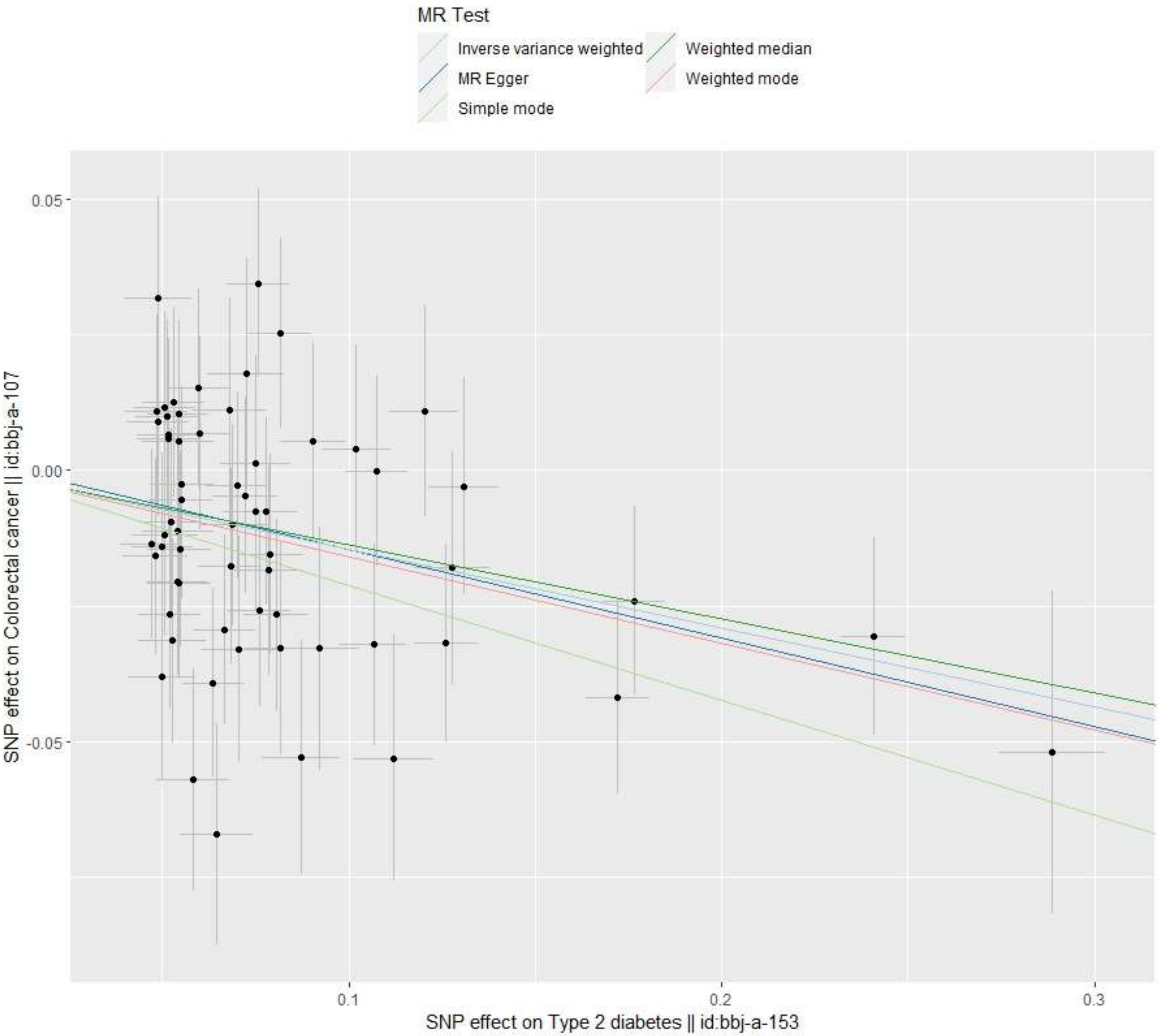
MR Test
Inverse variance weighted
MR Egger
Simple mode
Weighted median
Weighted mode
0.05
0.00
-0.05
0.1
0.2
0.3
SNP effect on Colorectal cancer || id:bbj-a-107
SNP effect on Type 2 diabetes || id:bbj-a-153

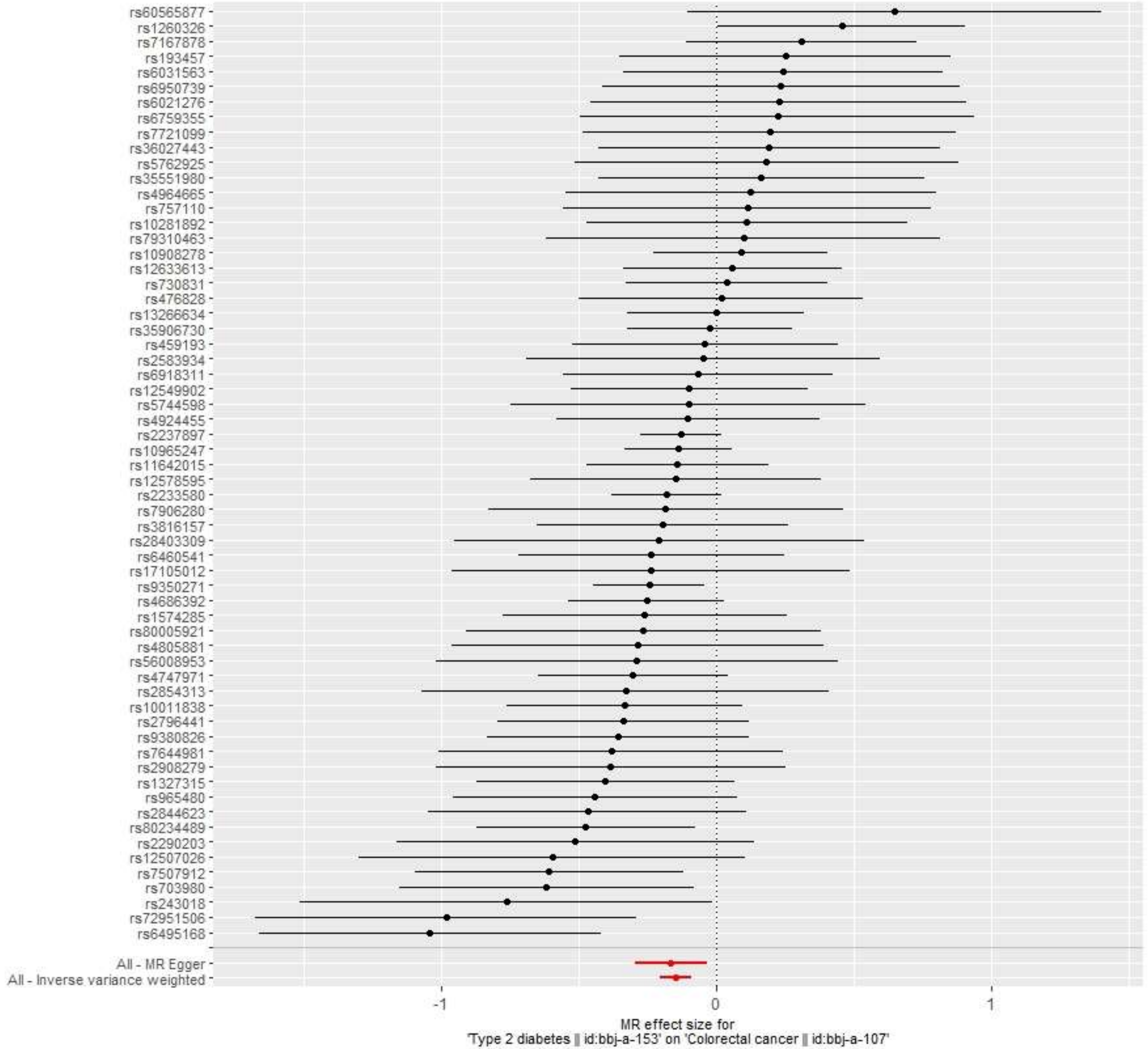
rs60565877
rs1260326
rs7167878
rs193457
rs6031563
rs6950739
rs6021276
rs6759355
rs7721099
rs36027443
rs5762925
rs35551980
rs4964665
rs757110
rs10281892
rs79310463
rs10908278
rs12633613
rs730831
rs476828
rs13266634
rs35906730
rs459193
rs2583934
rs6918311
rs12549902
rs5744598
rs4924455
rs2237897
rs10965247
rs11642015
rs12578595
rs2233580
rs7906280
rs3816157
rs28403309
rs6460541
rs17105012
rs9350271
rs4686392
rs1574285
rs80005921
rs4805881
rs56008953
rs4747971
rs2854313
rs10011838
rs2796441
rs9380826
rs7644981
rs2908279
rs1327315
rs965480
rs2844623
rs80234489
rs2290203
rs12507026
rs7507912
rs703980
rs243018
rs72951506
rs6495168
All - MR Egger
All - Inverse variance weighted
-1
0
1
MR effect size for
'Type 2 diabetes || id:bbj-a-153' on 'Colorectal cancer || id:bbj-a-107'

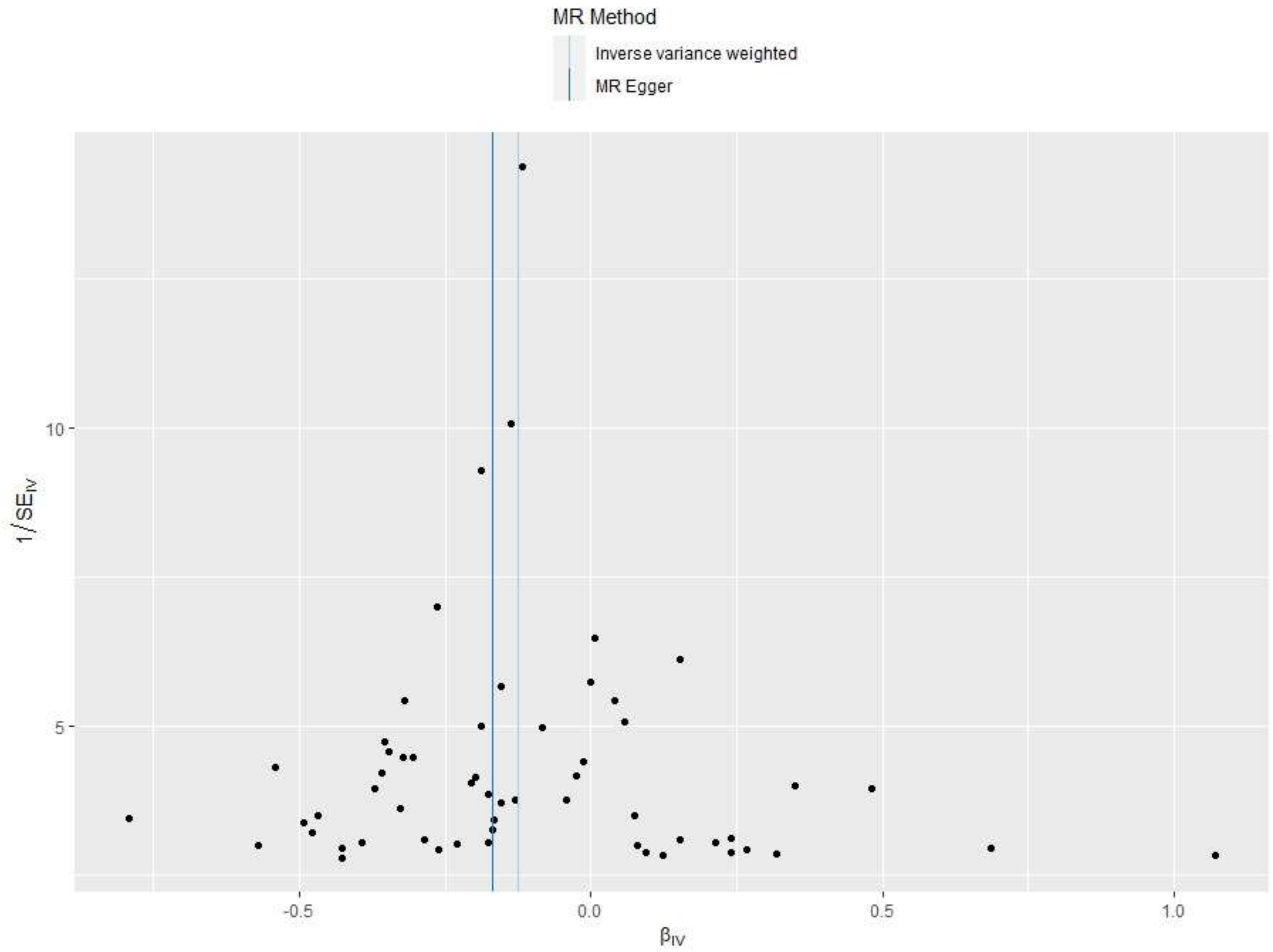
MR Method
Inverse variance weighted
MR Egger
10
5
1/SE_IV
-0.5
0.0
0.5
1.0
β_IV

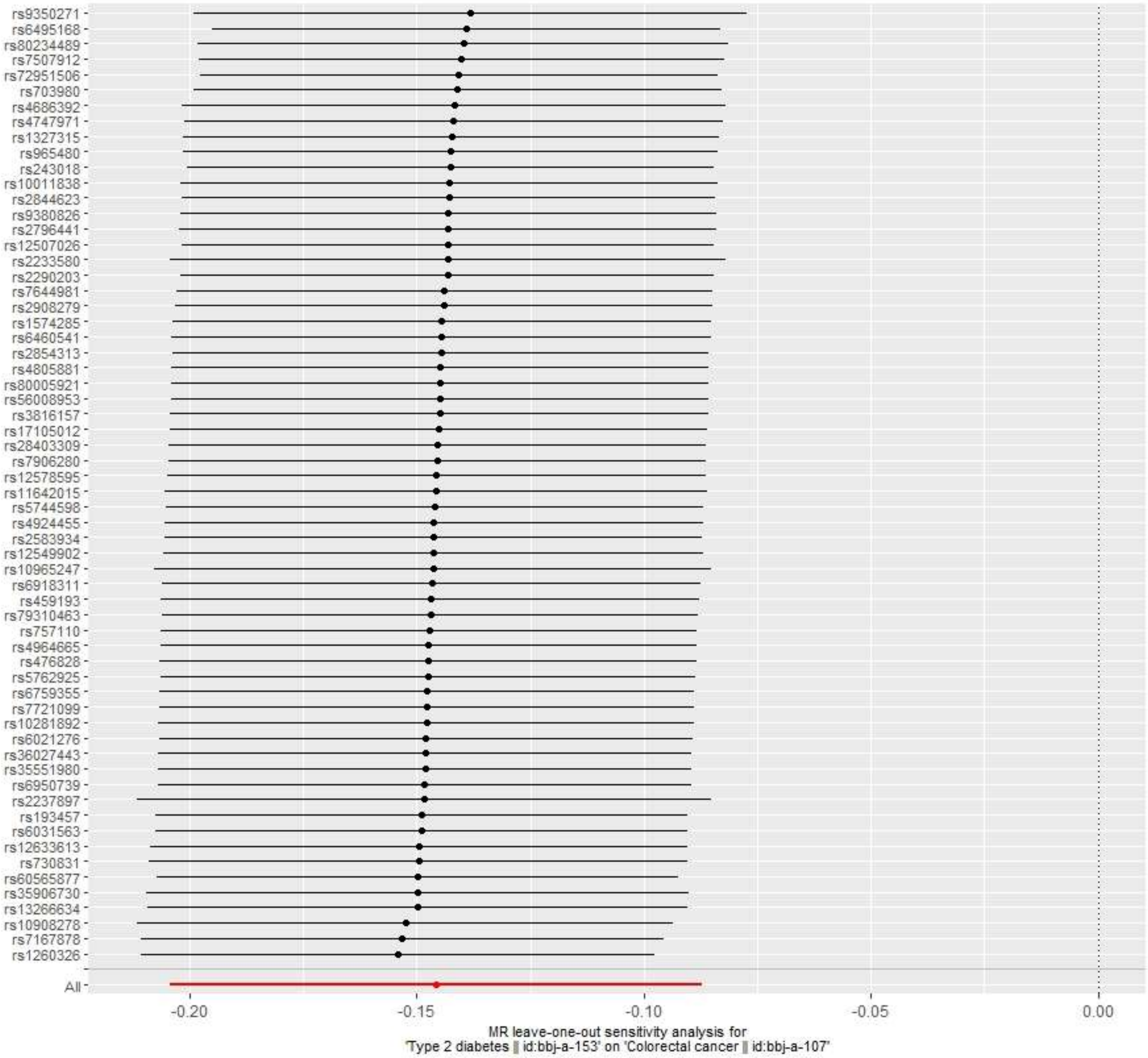
rs9350271
rs6495168
rs80234489
rs7507912
rs72951506
rs703980
rs4686392
rs4747971
rs1327315
rs965480
rs243018
rs10011838
rs2844623
rs9380826
rs2796441
rs12507026
rs2233580
rs2290203
rs7644981
rs2908279
rs1574285
rs6460541
rs2854313
rs4805881
rs80005921
rs56008953
rs3816157
rs17105012
rs28403309
rs7906280
rs12578595
rs11642015
rs5744598
rs4924455
rs2583934
rs12549902
rs10965247
rs6918311
rs459193
rs79310463
rs757110
rs4964665
rs476828
rs5762925
rs6759355
rs7721099
rs10281892
rs6021276
rs36027443
rs35551980
rs6950739
rs2237897
rs193457
rs6031563
rs12633613
rs730831
rs60565877
rs35906730
rs13266634
rs10908278
rs7167878
rs1260326
All
-0.20
-0.15
-0.10
-0.05
0.00
MR leave-one-out sensitivity analysis for
'Type 2 diabetes || id:bbj-a-153' on 'Colorectal cancer || id:bbj-a-107'
