# Supplementary material for: T2DM may exert a protective effect against digestive system tumors in East Asian populations: a Mendelian randomization analysis
Source: Front Oncol. 2024 Jun 14;14:1327154. doi: 10.3389/fonc.2024.1327154 (PMC11211363; doi:10.3389/fonc.2024.1327154)
Supplement: Supplemental File 2 — The plot of MR result of T2DM on esophageal cancer in East Asian. [file DataSheet_2.pdf]

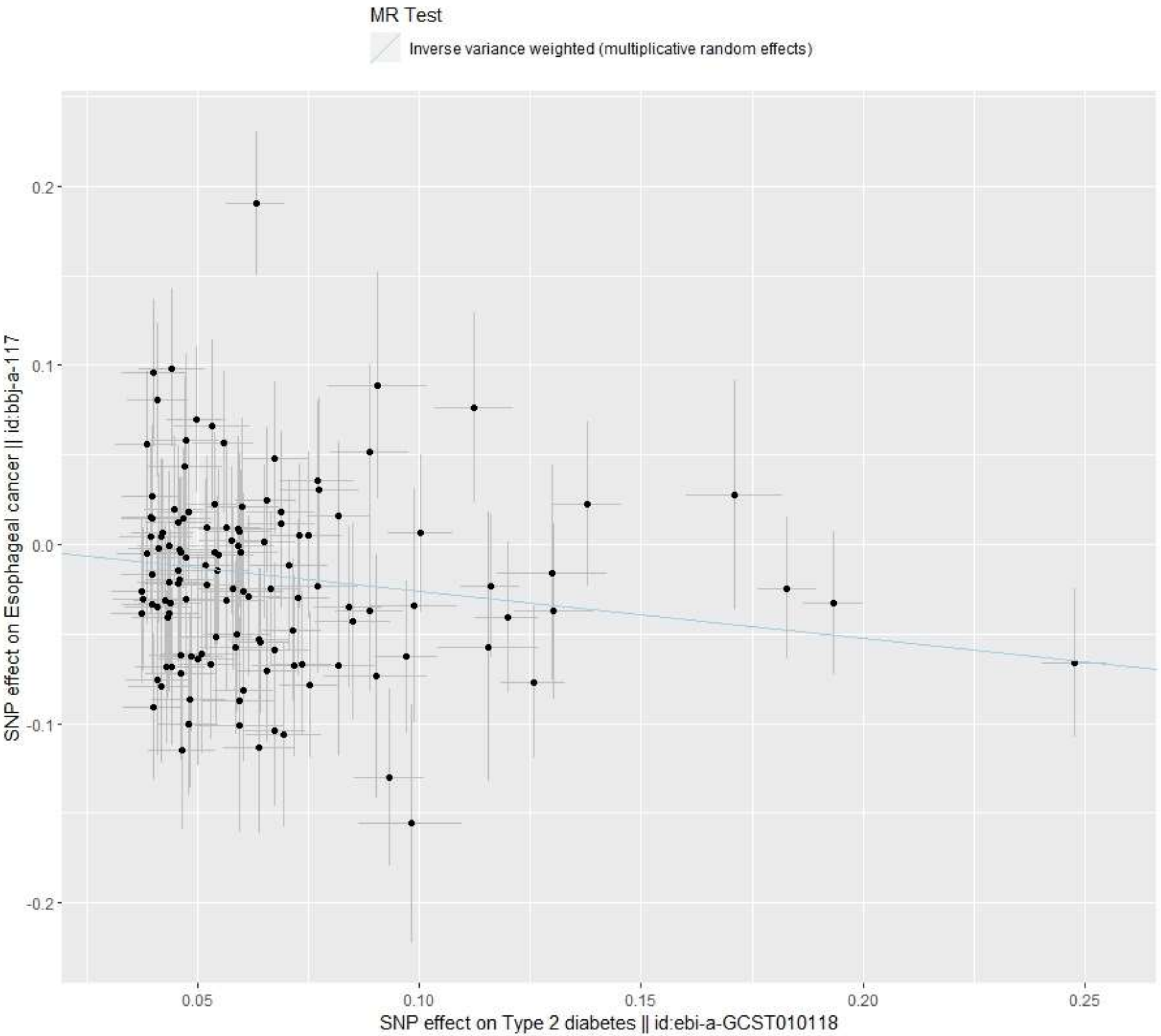
MR Test
Inverse variance weighted (multiplicative random effects)
SNP effect on Esophageal cancer || id:bbj-a-117
0.2
0.1
0.0
-0.1
-0.2
0.05
0.10
0.15
0.20
0.25
SNP effect on Type 2 diabetes || id:ebi-a-GCST010118

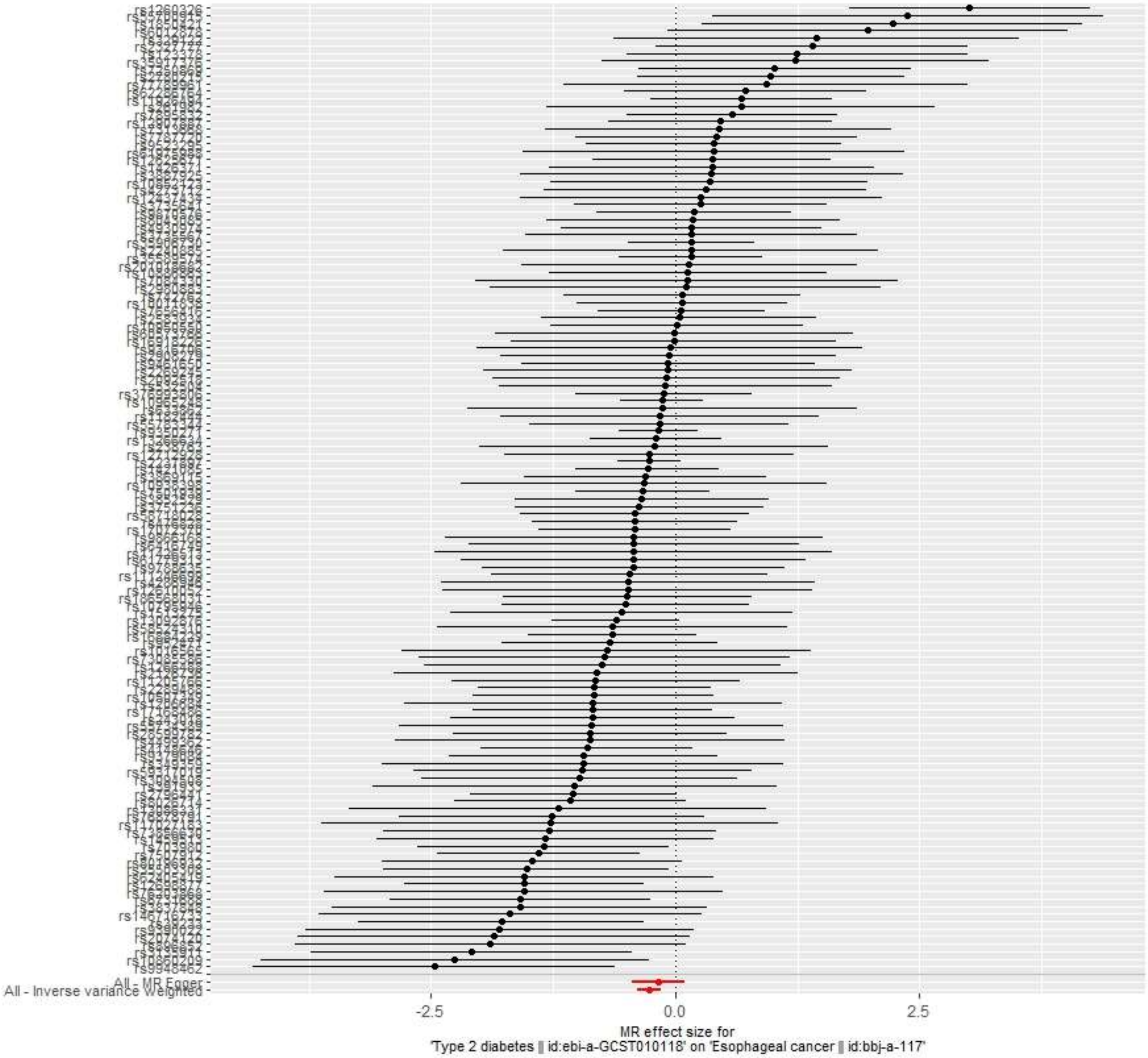
All - MR Egger
All - Inverse variance weighted
-2.5
0.0
2.5
MR effect size for
'Type 2 diabetes || id:ebi-a-GCST010118' on 'Esophageal cancer || id:bbj-a-117'

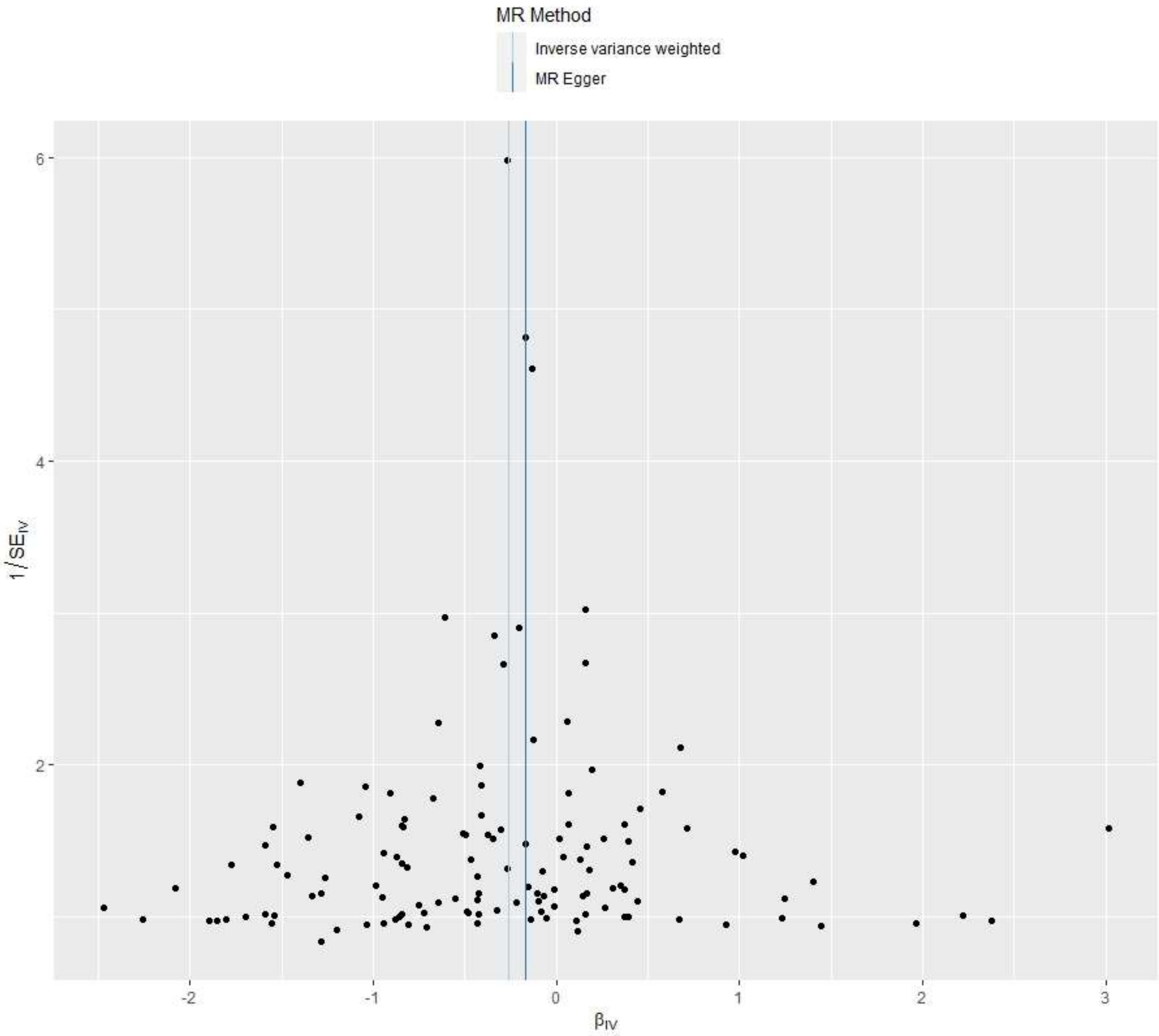
MR Method
Inverse variance weighted
MR Egger
6
4
2
1/SE$_{IV}$
-2
-1
0
1
2
3
β$_{IV}$

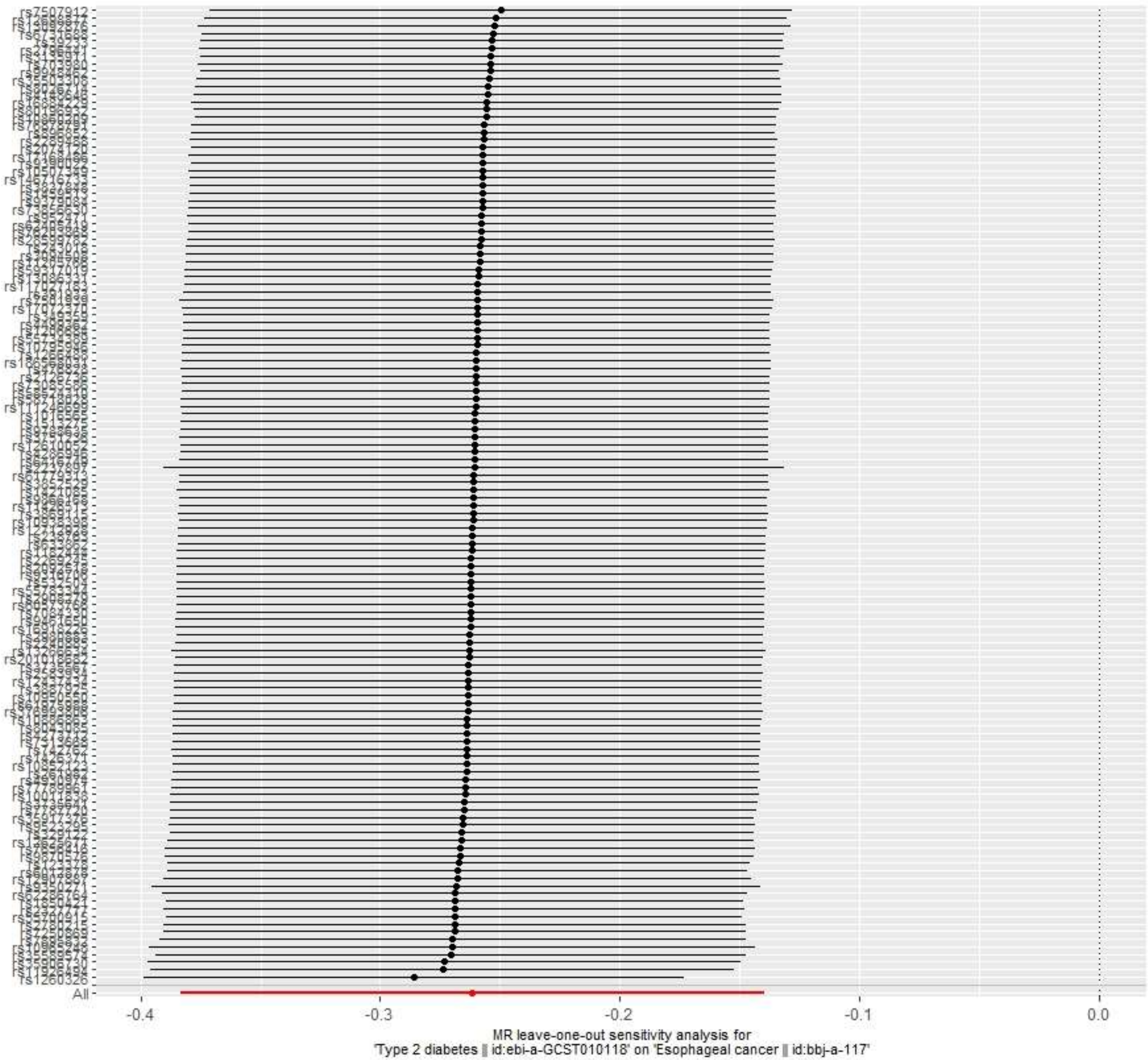
rs7507912
rs1260326
All
-0.4
-0.3
-0.2
-0.1
0.0
MR leave-one-out sensitivity analysis for
'Type 2 diabetes || id:ebi-a-GCST010118' on 'Esophageal cancer || id:bbj-a-117'

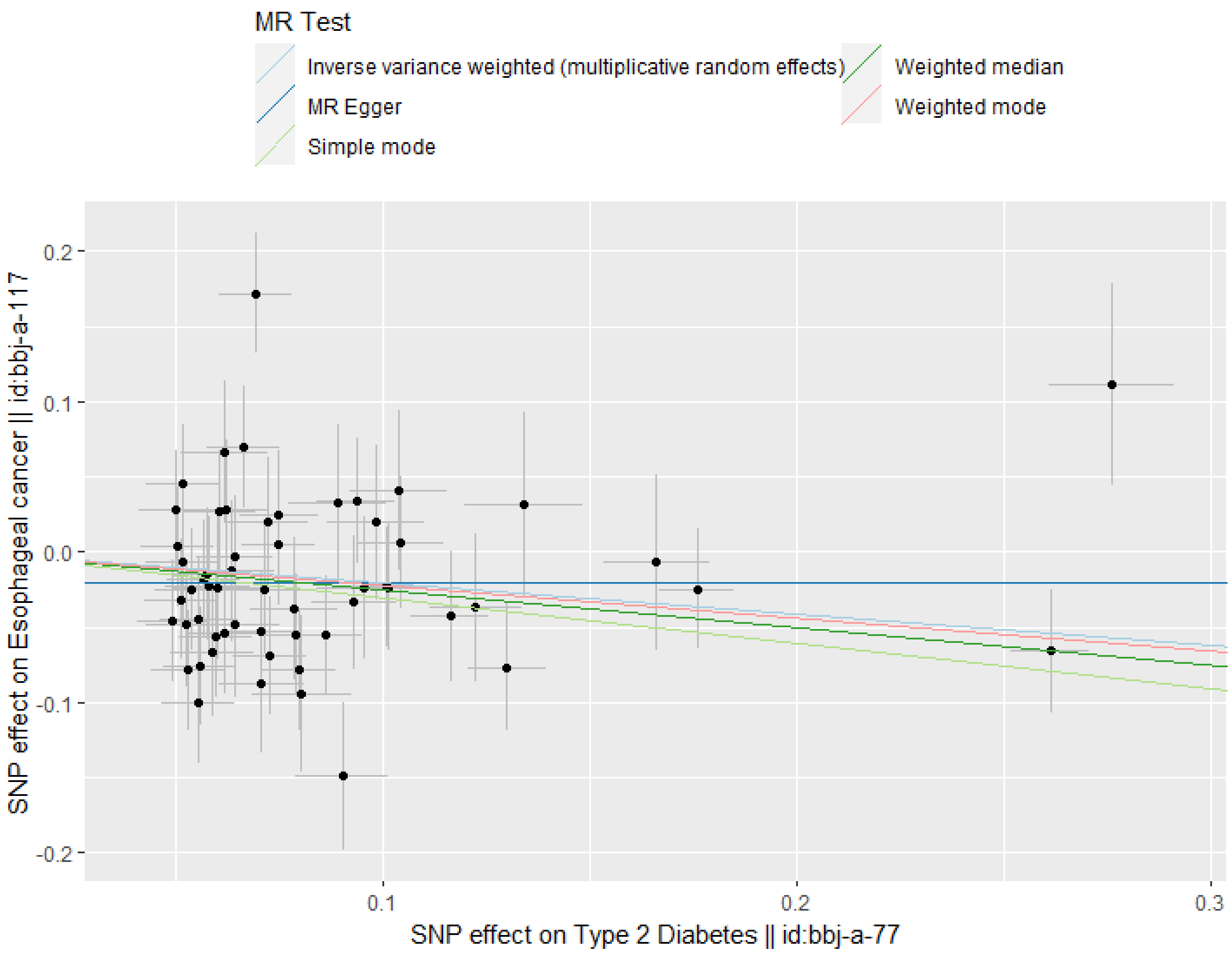
MR Test
Inverse variance weighted (multiplicative random effects)
MR Egger
Simple mode
Weighted median
Weighted mode
SNP effect on Esophageal cancer || id:bbj-a-117
SNP effect on Type 2 Diabetes || id:bbj-a-77
0.2
0.1
0.0
-0.1
-0.2
0.1
0.2
0.3

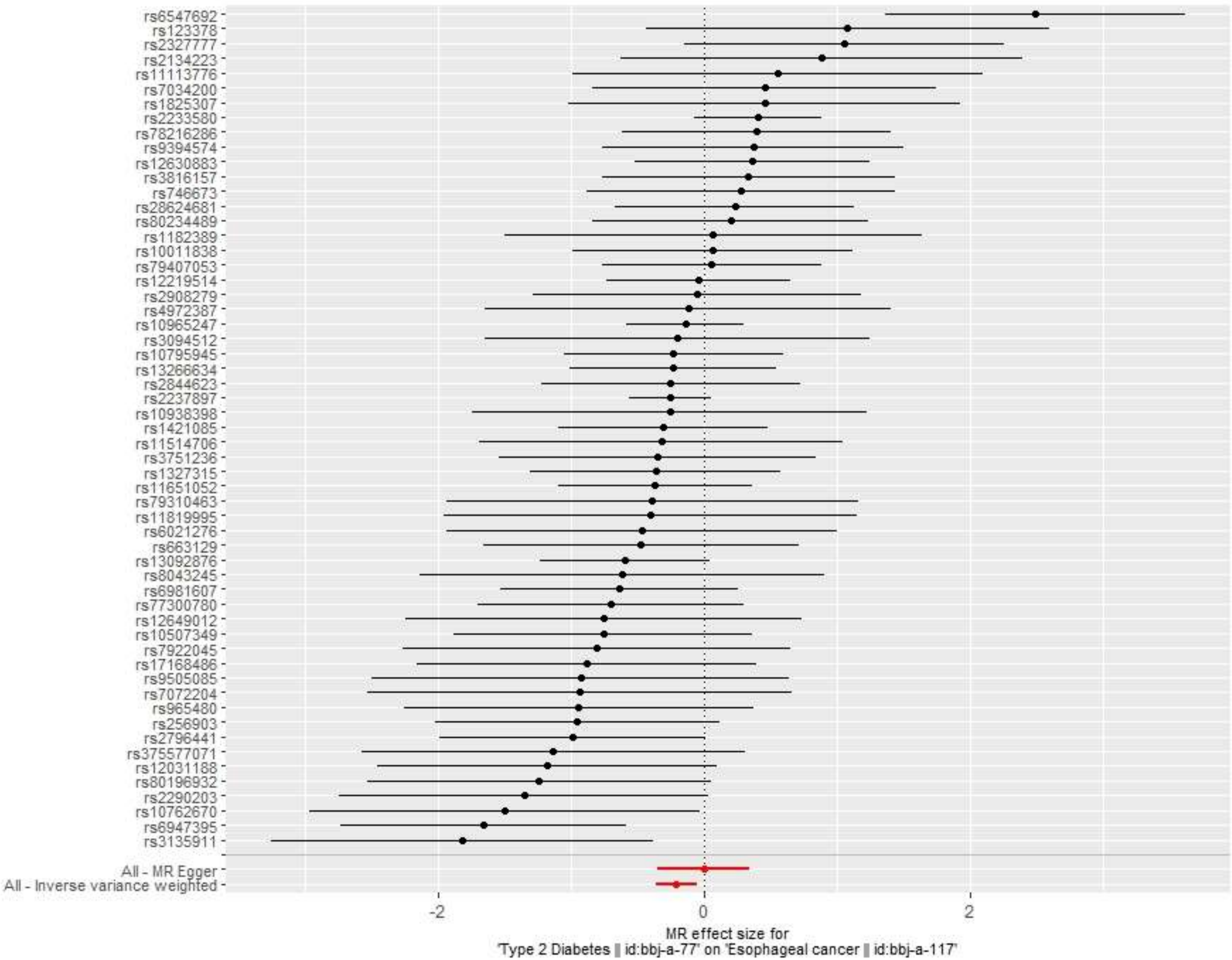
rs6547692
rs123378
rs2327777
rs2134223
rs11113776
rs7034200
rs1825307
rs2233580
rs78216286
rs9394574
rs12630883
rs3816157
rs746673
rs28624681
rs80234489
rs1182389
rs10011838
rs79407053
rs12219514
rs2908279
rs4972387
rs10965247
rs3094512
rs10795945
rs13266634
rs2844623
rs2237897
rs10938398
rs1421085
rs11514706
rs3751236
rs1327315
rs11651052
rs79310463
rs11819995
rs6021276
rs663129
rs13092876
rs8043245
rs6981607
rs77300780
rs12649012
rs10507349
rs7922045
rs17168486
rs9505085
rs7072204
rs965480
rs256903
rs2796441
rs375577071
rs12031188
rs80196932
rs2290203
rs10762670
rs6947395
rs3135911
All - MR Egger
All - Inverse variance weighted
-2
0
2
MR effect size for
'Type 2 Diabetes || id:bbj-a-77' on 'Esophageal cancer || id:bbj-a-117'

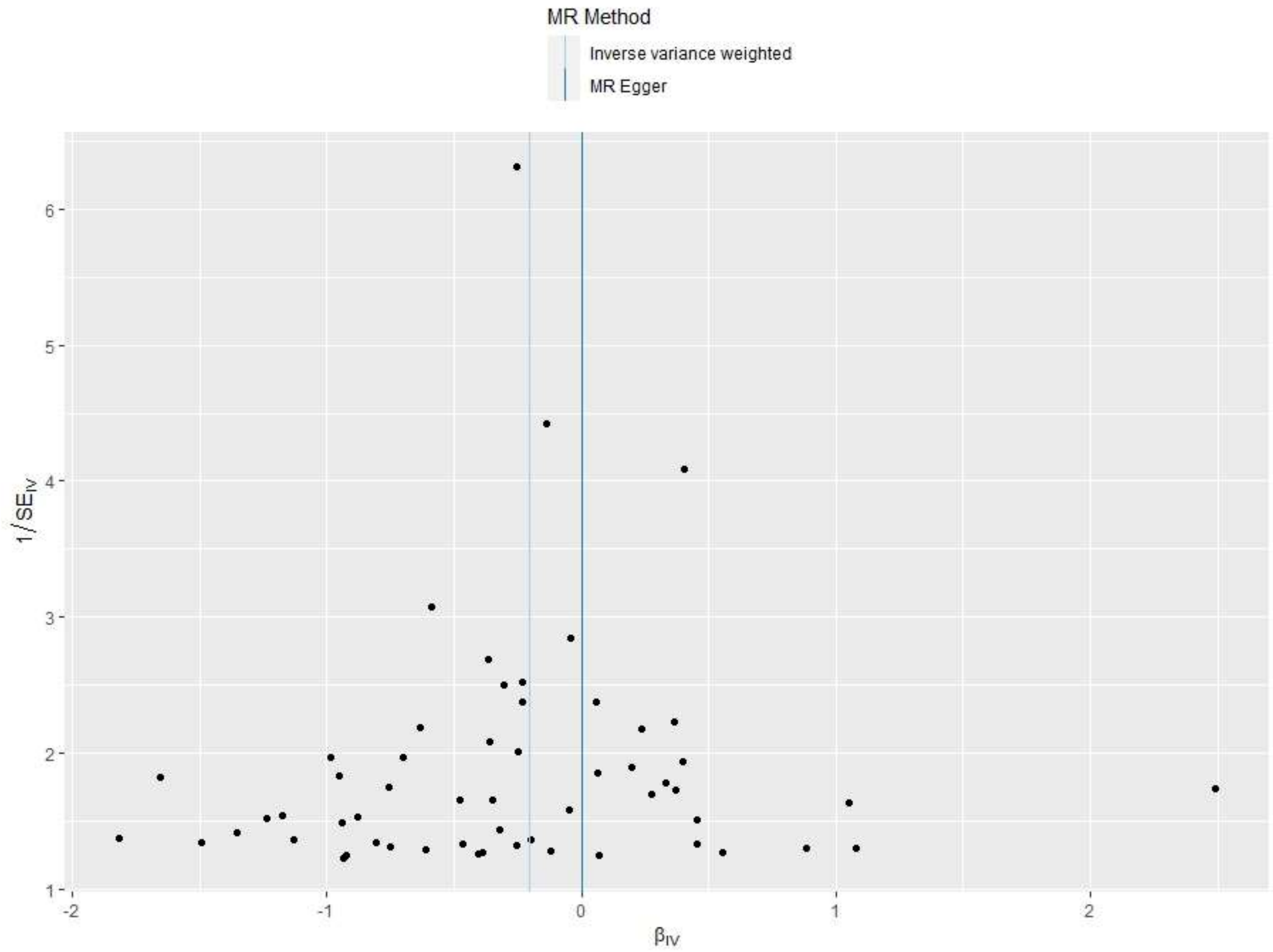
MR Method
Inverse variance weighted
MR Egger
1/SE$_{IV}$
β$_{IV}$
6
5
4
3
2
1
-2
-1
0
1
2

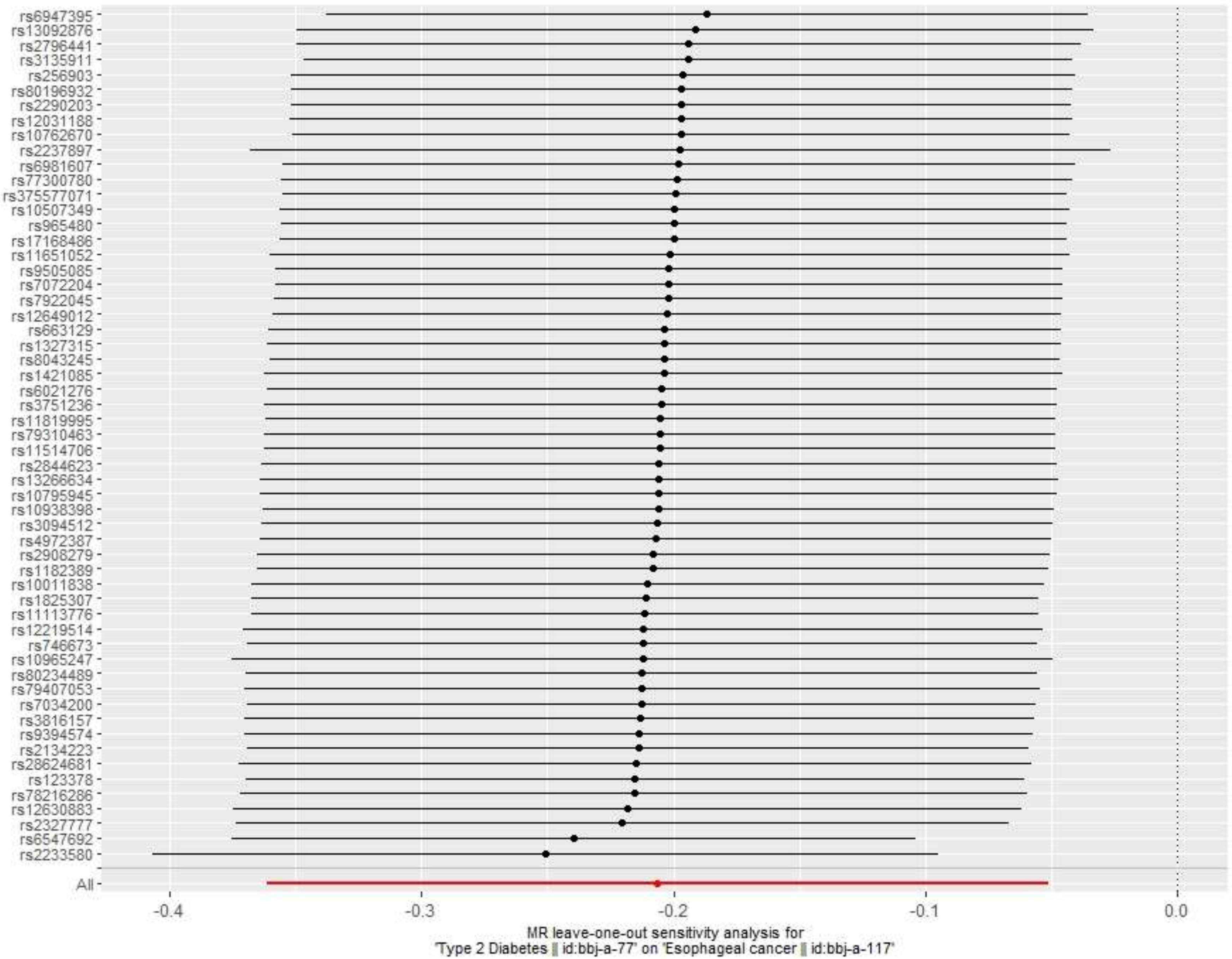

rs6947395
rs13092876
rs2796441
rs3135911
rs256903
rs80196932
rs2290203
rs12031188
rs10762670
rs2237897
rs6981607
rs77300780
rs375577071
rs10507349
rs965480
rs17168486
rs11651052
rs9505085
rs7072204
rs7922045
rs12649012
rs663129
rs1327315
rs8043245
rs1421085
rs6021276
rs3751236
rs11819995
rs79310463
rs11514706
rs2844623
rs13266634
rs10795945
rs10938398
rs3094512
rs4972387
rs2908279
rs1182389
rs10011838
rs1825307
rs11113776
rs12219514
rs746673
rs10965247
rs80234489
rs79407053
rs7034200
rs3816157
rs9394574
rs2134223
rs28624681
rs123378
rs78216286
rs12630883
rs2327777
rs6547692
rs2233580
All
-0.4
-0.3
-0.2
-0.1
0.0
MR leave-one-out sensitivity analysis for
'Type 2 Diabetes || id:bbj-a-77' on 'Esophageal cancer || id:bbj-a-117'

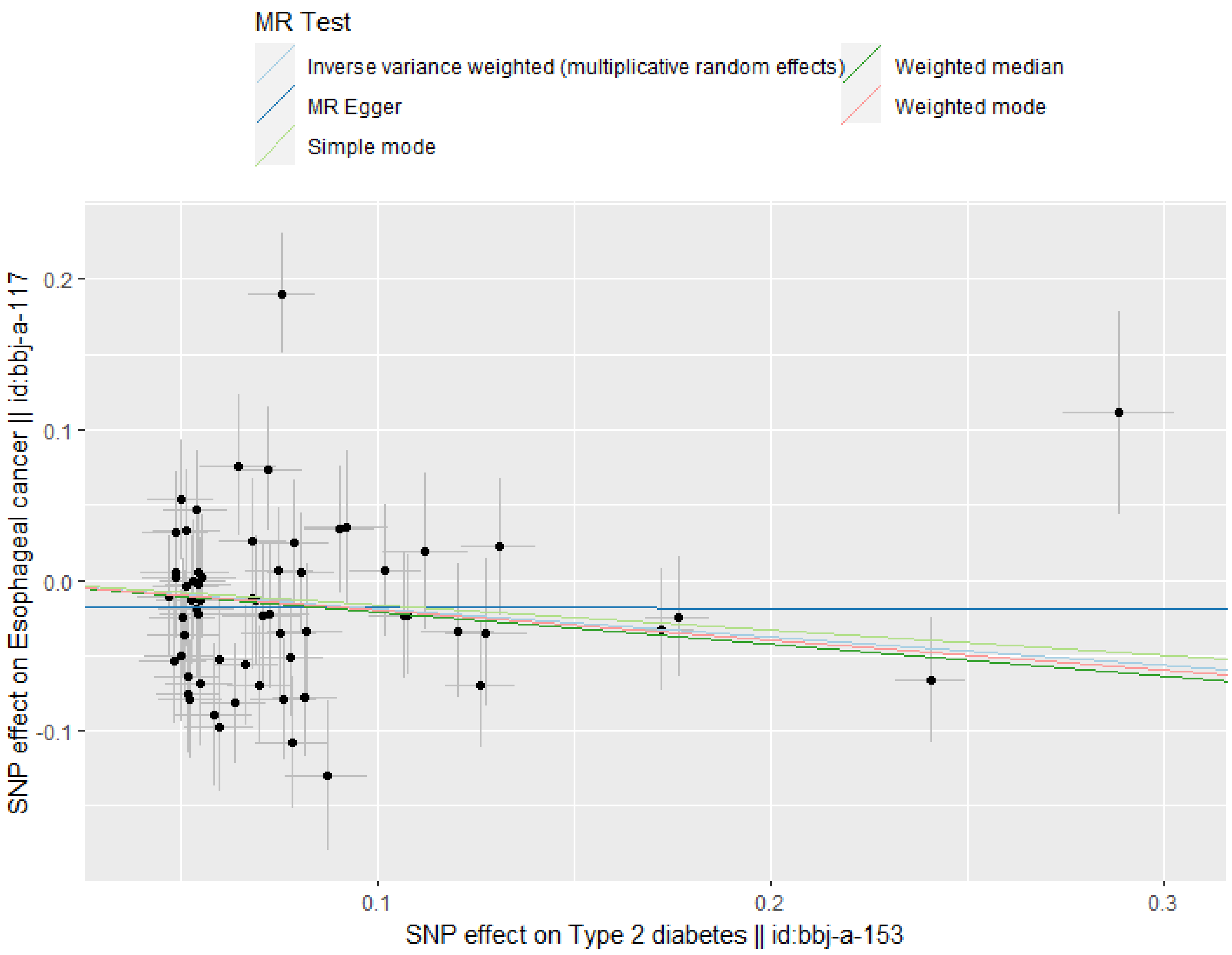
MR Test
Inverse variance weighted (multiplicative random effects)
MR Egger
Simple mode
Weighted median
Weighted mode
0.2
0.1
0.0
-0.1
SNP effect on Esophageal cancer || id:bbj-a-117
0.1
0.2
0.3
SNP effect on Type 2 diabetes || id:bbj-a-153

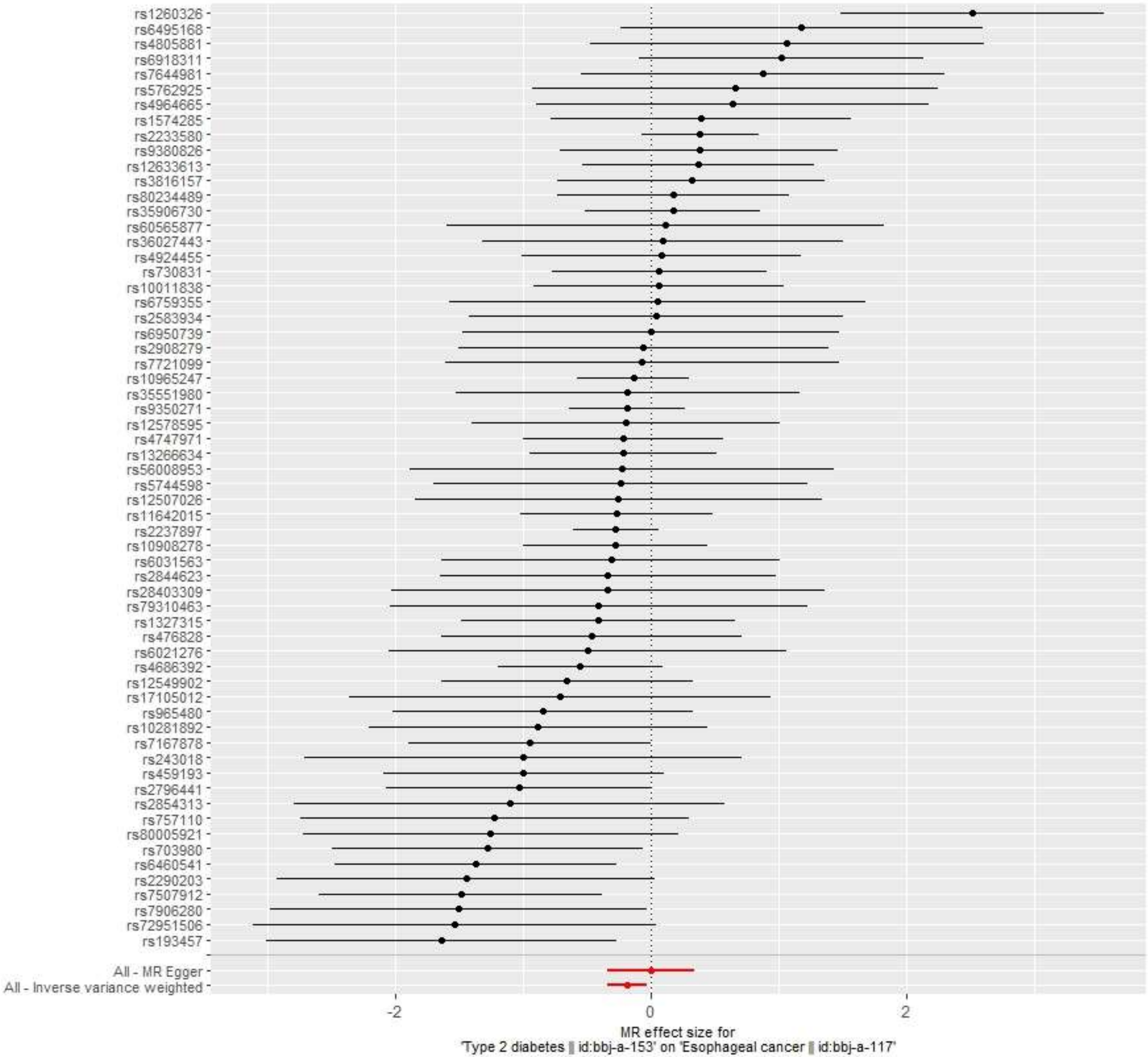
rs1260326
rs6495168
rs4805881
rs6918311
rs7644981
rs5762925
rs4964665
rs1574285
rs2233580
rs9380826
rs12633613
rs3816157
rs80234489
rs35906730
rs60565877
rs36027443
rs4924455
rs730831
rs10011838
rs6759355
rs2583934
rs6950739
rs2908279
rs7721099
rs10965247
rs35551980
rs9350271
rs12578595
rs4747971
rs13266634
rs56008953
rs5744598
rs12507026
rs11642015
rs2237897
rs10908278
rs6031563
rs2844623
rs28403309
rs79310463
rs1327315
rs476828
rs6021276
rs4686392
rs12549902
rs17105012
rs965480
rs10281892
rs7167878
rs243018
rs459193
rs2796441
rs2854313
rs757110
rs80005921
rs703980
rs6460541
rs2290203
rs7507912
rs7906280
rs72951506
rs193457
All - MR Egger
All - Inverse variance weighted
-2
0
2
MR effect size for
'Type 2 diabetes || id:bbj-a-153' on 'Esophageal cancer || id:bbj-a-117'

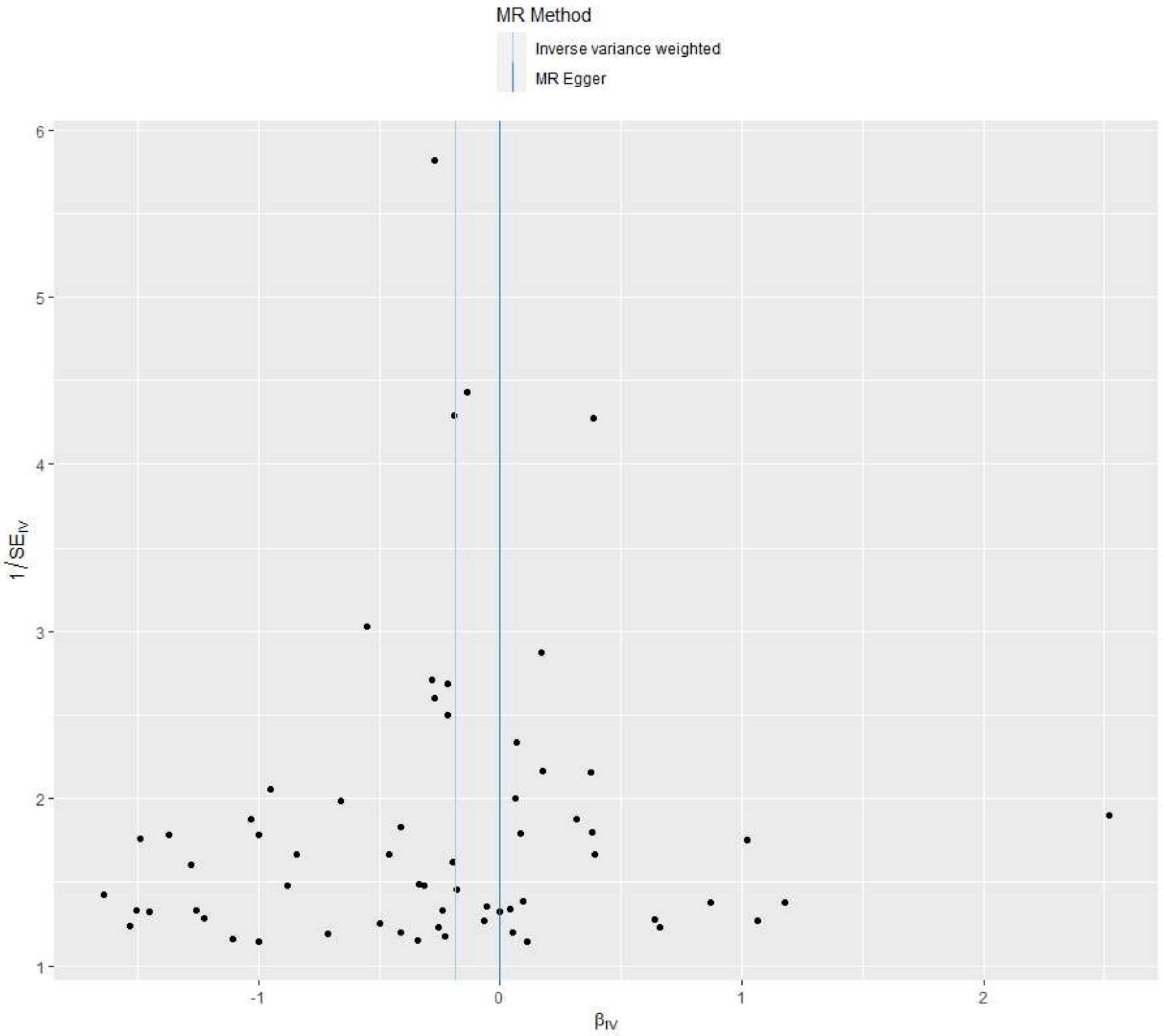
MR Method
Inverse variance weighted
MR Egger
$1/SE_{IV}$
β_{IV}
6
5
4
3
2
1
-1
0
1
2

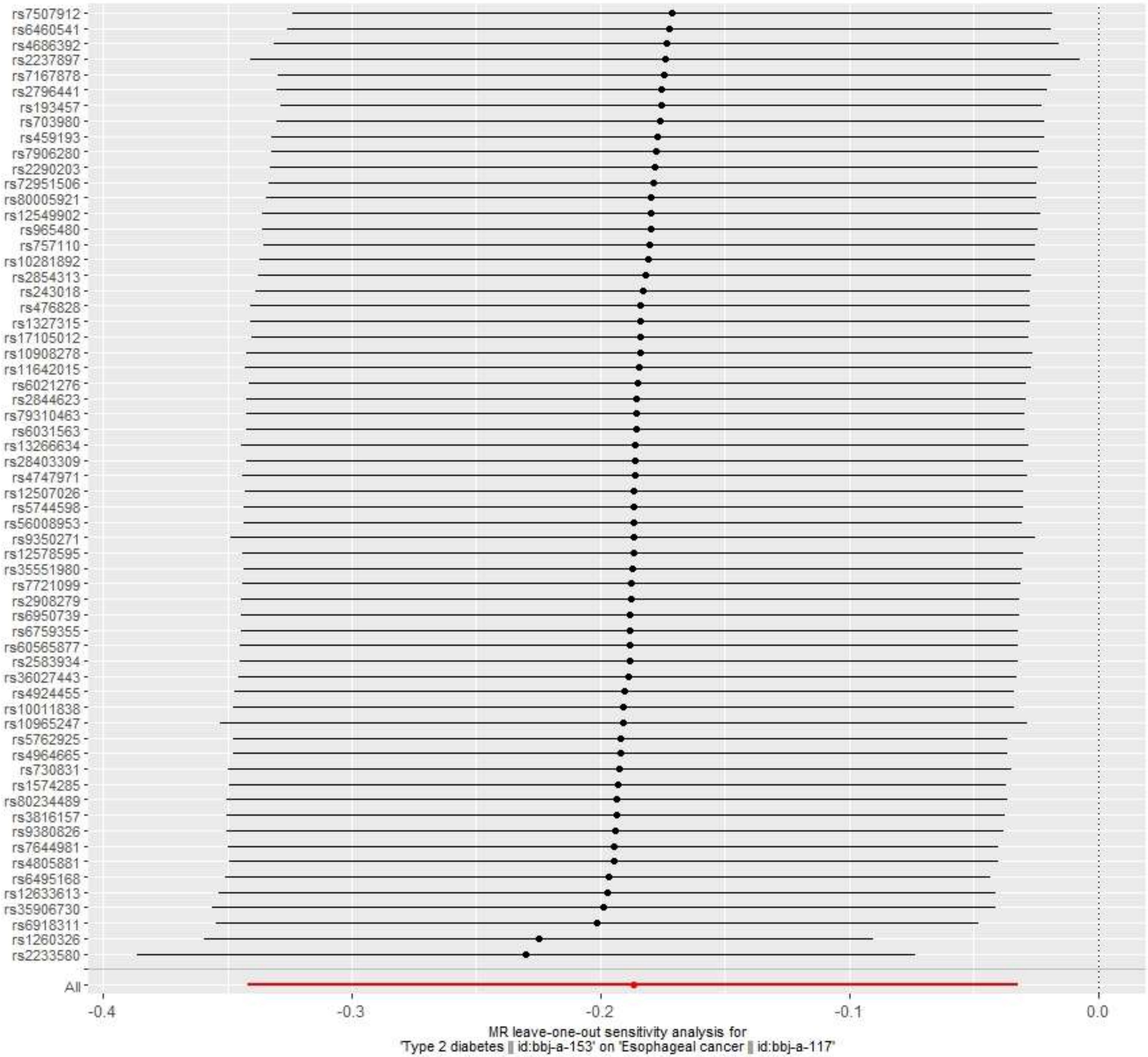
rs7507912
rs6460541
rs4686392
rs2237897
rs7167878
rs2796441
rs193457
rs703980
rs459193
rs7906280
rs2290203
rs72951506
rs80005921
rs12549902
rs965480
rs757110
rs10281892
rs2854313
rs243018
rs476828
rs1327315
rs17105012
rs10908278
rs11642015
rs6021276
rs2844623
rs79310463
rs6031563
rs13266634
rs28403309
rs4747971
rs12507026
rs5744598
rs56008953
rs9350271
rs12578595
rs35551980
rs7721099
rs2908279
rs6950739
rs6759355
rs60565877
rs2583934
rs36027443
rs4924455
rs10011838
rs10965247
rs5762925
rs4964665
rs730831
rs1574285
rs80234489
rs3816157
rs9380826
rs7644981
rs4805881
rs6495168
rs12633613
rs35906730
rs6918311
rs1260326
rs2233580
All
-0.4
-0.3
-0.2
-0.1
0.0
MR leave-one-out sensitivity analysis for
'Type 2 diabetes || id:bbj-a-153' on 'Esophageal cancer || id:bbj-a-117'
