# Supplementary material for: T2DM may exert a protective effect against digestive system tumors in East Asian populations: a Mendelian randomization analysis
Source: Front Oncol. 2024 Jun 14;14:1327154. doi: 10.3389/fonc.2024.1327154 (PMC11211363; doi:10.3389/fonc.2024.1327154)
Supplement: Supplemental File 3 — The plot of MR result of T2DM on gastric cancer in East Asian. [file DataSheet_3.pdf]

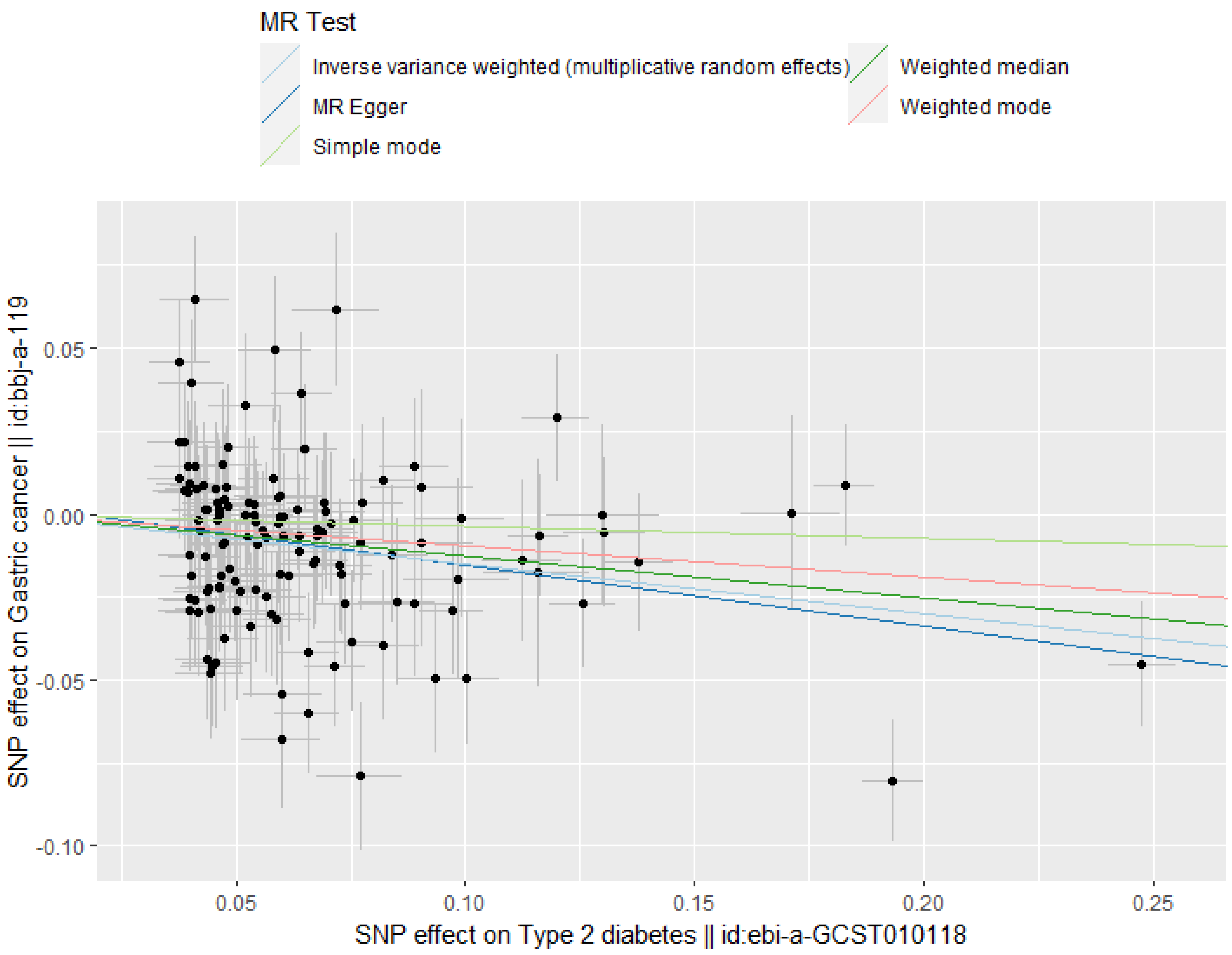

MR Test
Inverse variance weighted (multiplicative random effects)
MR Egger
Simple mode
Weighted median
Weighted mode
SNP effect on Gastric cancer || id:bbj-a-119
0.05
0.00
-0.05
-0.10
0.05
0.10
0.15
0.20
0.25
SNP effect on Type 2 diabetes || id:ebi-a-GCST010118

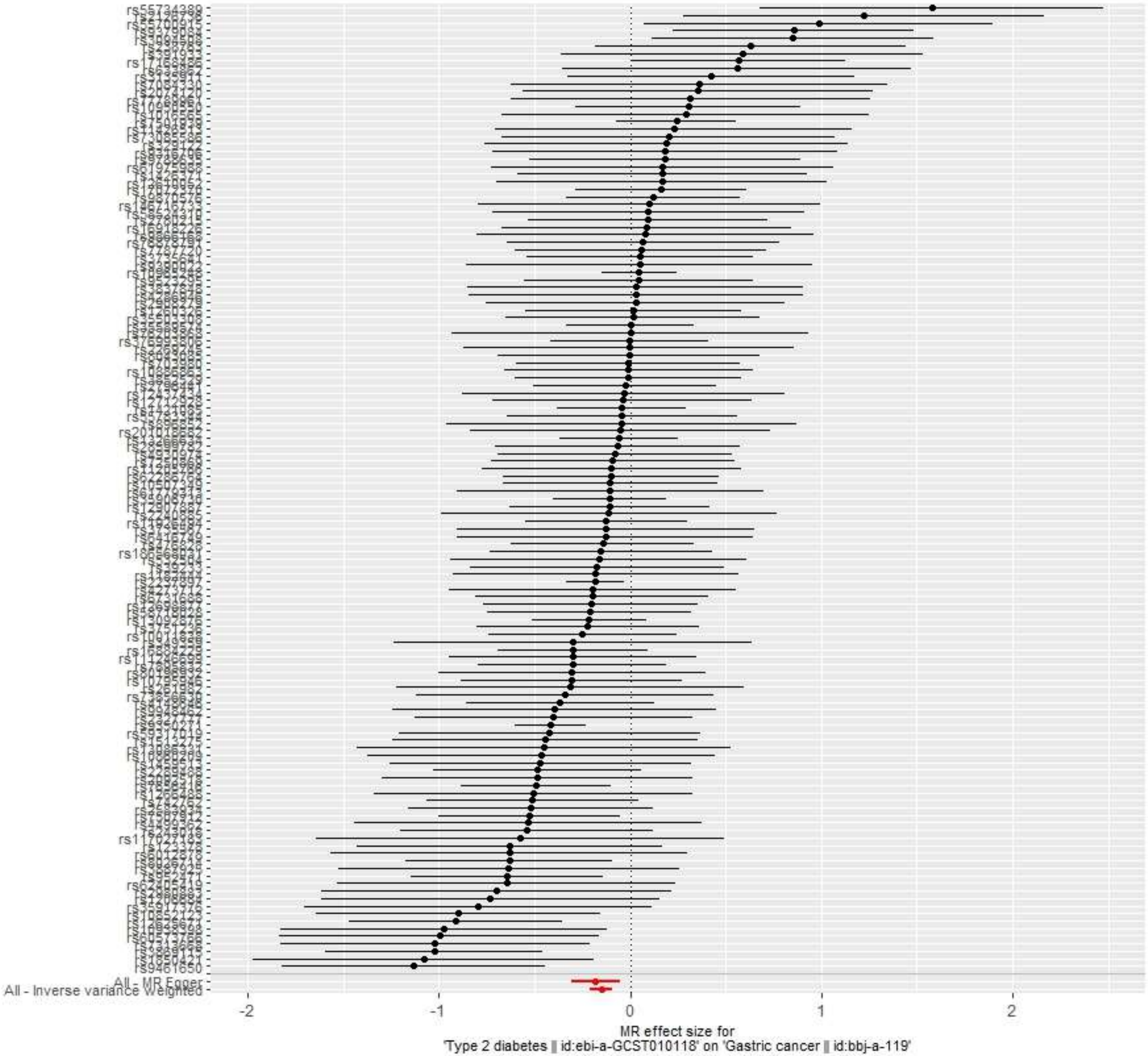
rs55734389
rs9461650
All - MR Egger
All - Inverse variance weighted
-2
-1
0
1
2
MR effect size for
'Type 2 diabetes || id:ebi-a-GCST010118' on 'Gastric cancer || id:bbj-a-119'

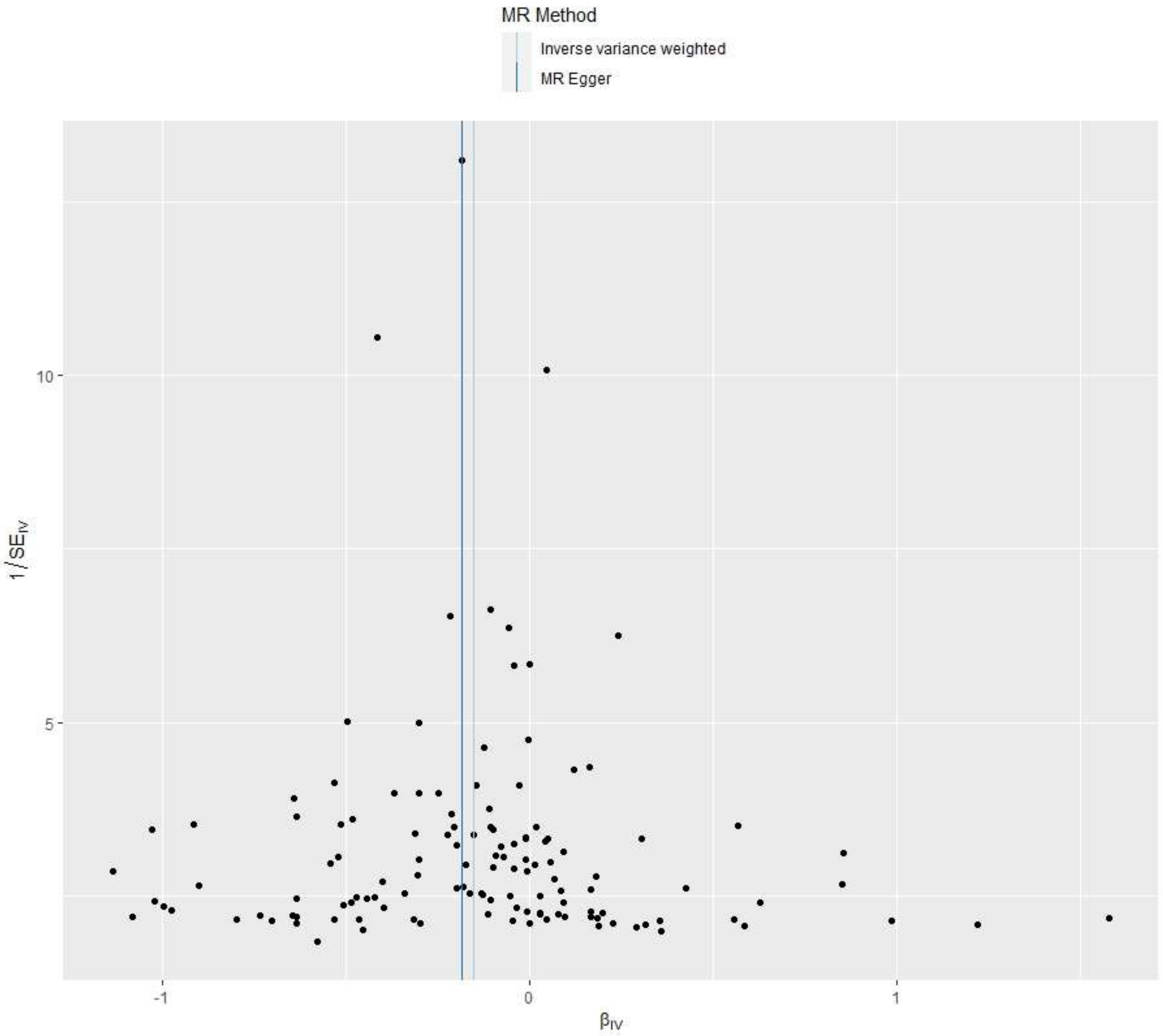
MR Method
Inverse variance weighted
MR Egger
10
5
1/SE_IV
-1
0
1
β_IV

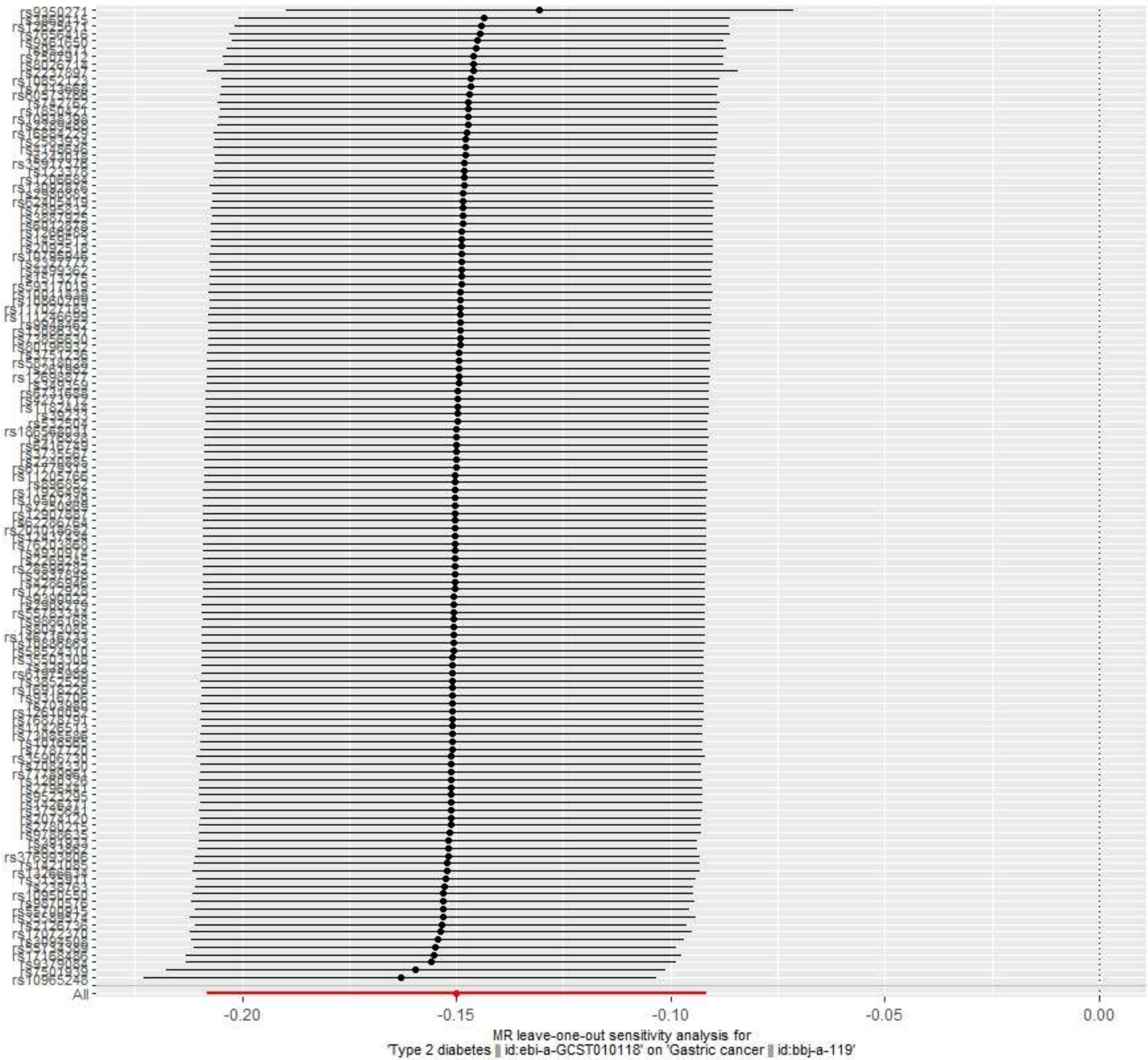

rs9350271
rs10965248
All
-0.20
-0.15
-0.10
-0.05
0.00
MR leave-one-out sensitivity analysis for
'Type 2 diabetes || id:ebi-a-GCST010118' on 'Gastric cancer || id:bbj-a-119'

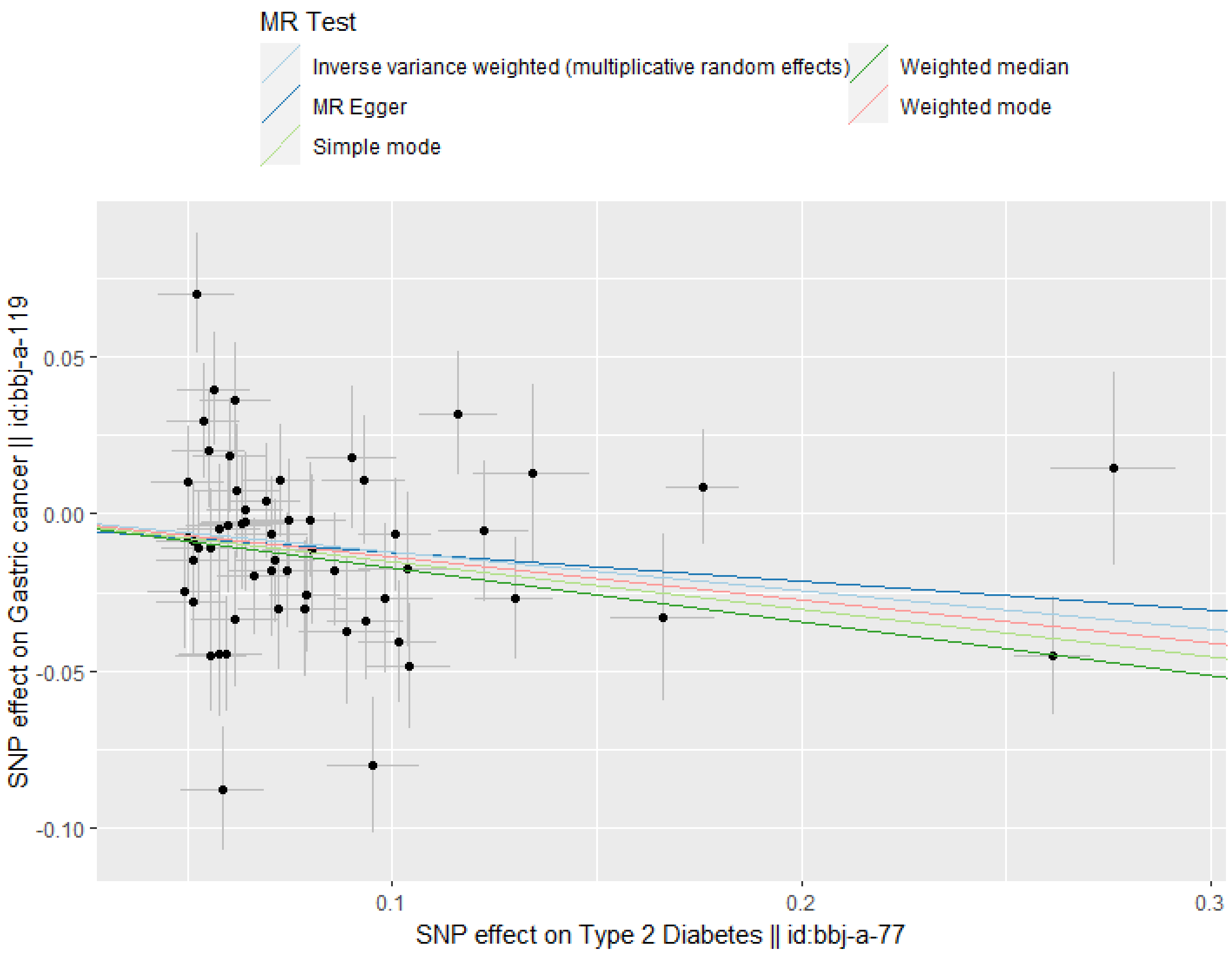
MR Test
Inverse variance weighted (multiplicative random effects)
MR Egger
Simple mode
Weighted median
Weighted mode
SNP effect on Gastric cancer || id:bbj-a-119
0.05
0.00
-0.05
-0.10
0.1
0.2
0.3
SNP effect on Type 2 Diabetes || id:bbj-a-77

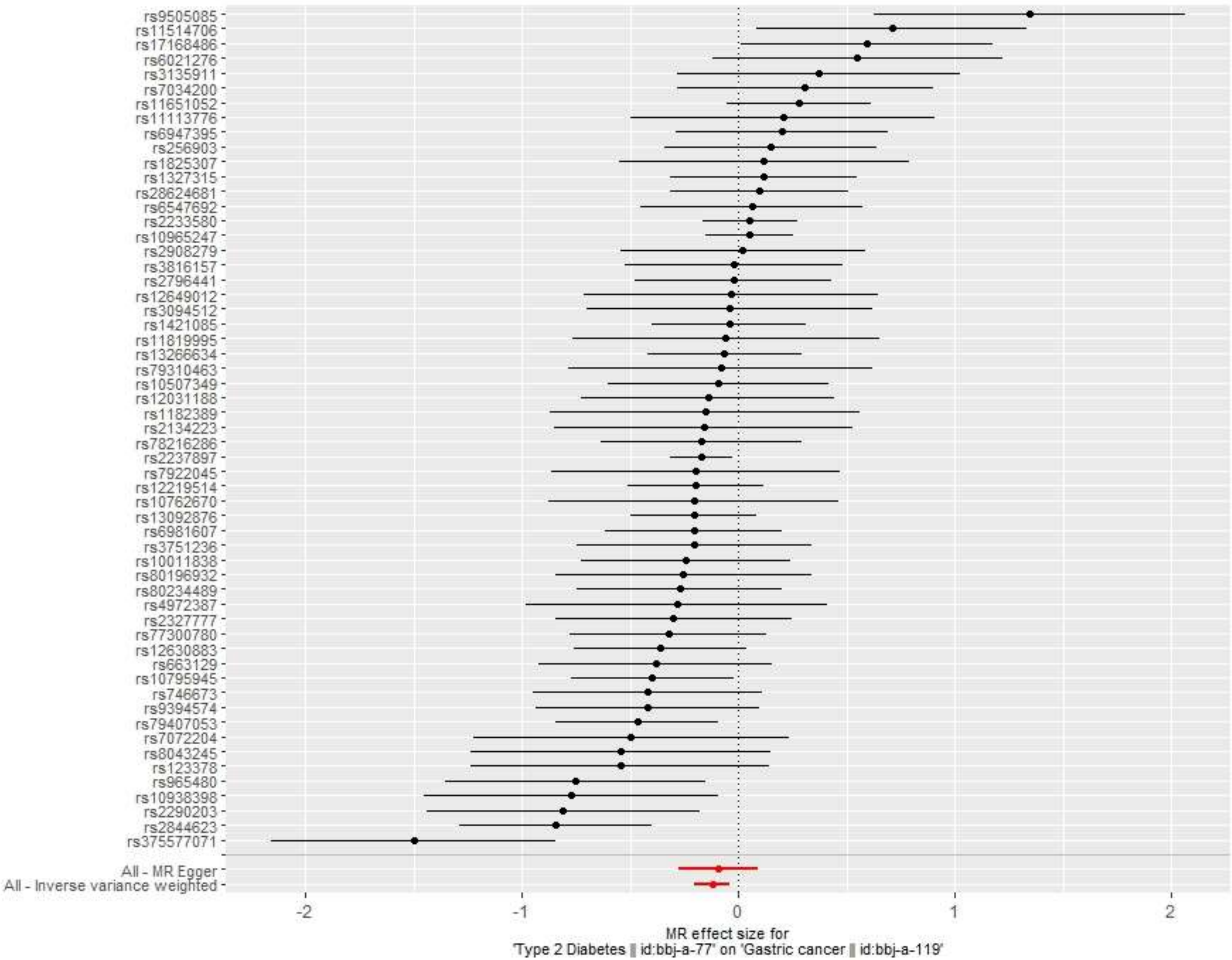
rs9505085
rs11514706
rs17168486
rs6021276
rs3135911
rs7034200
rs11651052
rs11113776
rs6947395
rs256903
rs1825307
rs1327315
rs28624681
rs6547692
rs2233580
rs10965247
rs2908279
rs3816157
rs2796441
rs12649012
rs3094512
rs1421085
rs11819995
rs13266634
rs79310463
rs10507349
rs12031188
rs1182389
rs2134223
rs78216286
rs2237897
rs7922045
rs12219514
rs10762670
rs13092876
rs6981607
rs3751236
rs10011838
rs80196932
rs80234489
rs4972387
rs2327777
rs77300780
rs12630883
rs663129
rs10795945
rs746673
rs9394574
rs79407053
rs7072204
rs8043245
rs123378
rs965480
rs10938398
rs2290203
rs2844623
rs375577071
All - MR Egger
All - Inverse variance weighted
-2
-1
0
1
2
MR effect size for
'Type 2 Diabetes || id:bbj-a-77' on 'Gastric cancer || id:bbj-a-119'

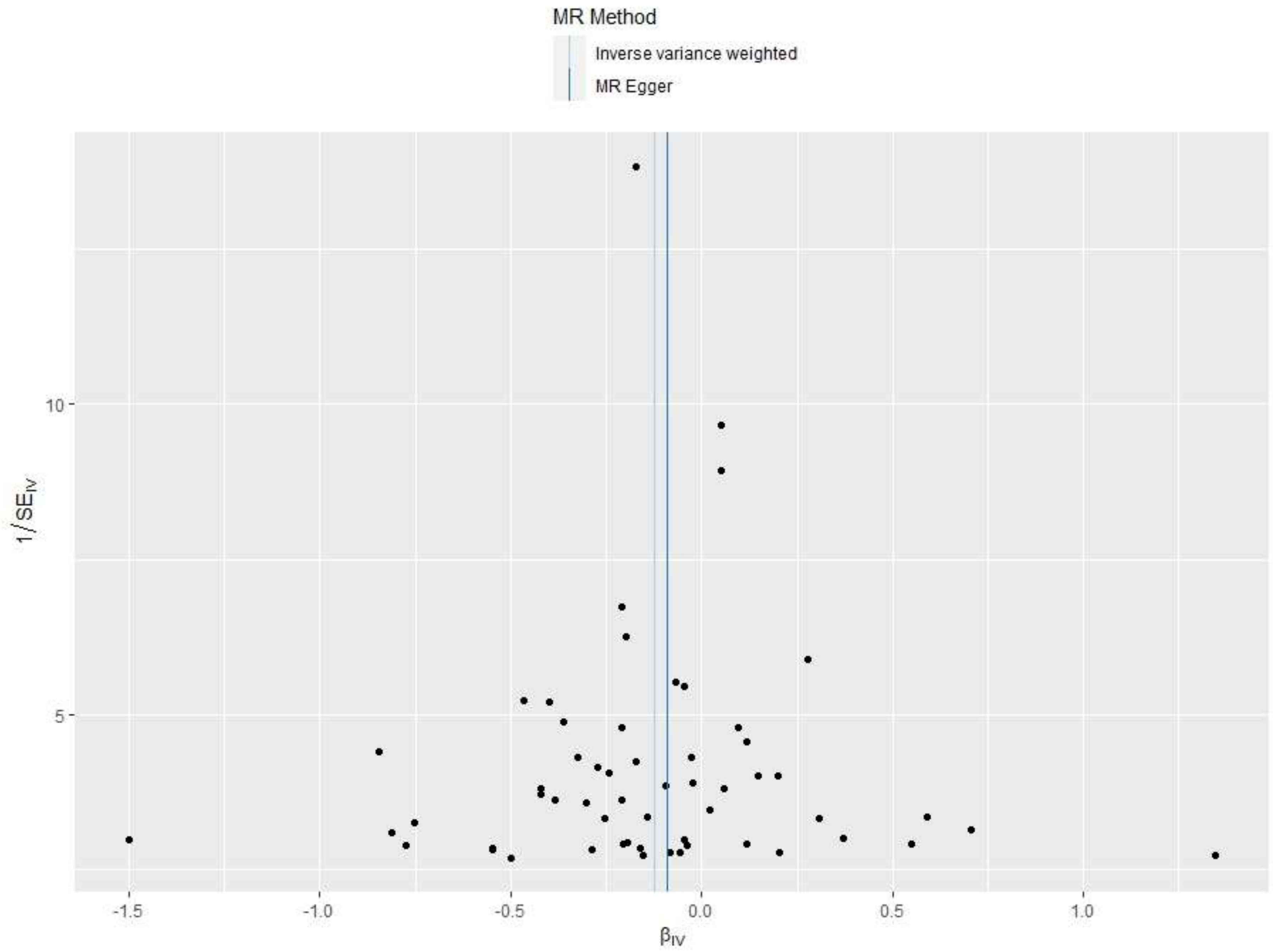
MR Method
Inverse variance weighted
MR Egger
10
5
1/SE_IV
-1.5
-1.0
-0.5
0.0
0.5
1.0
β_IV

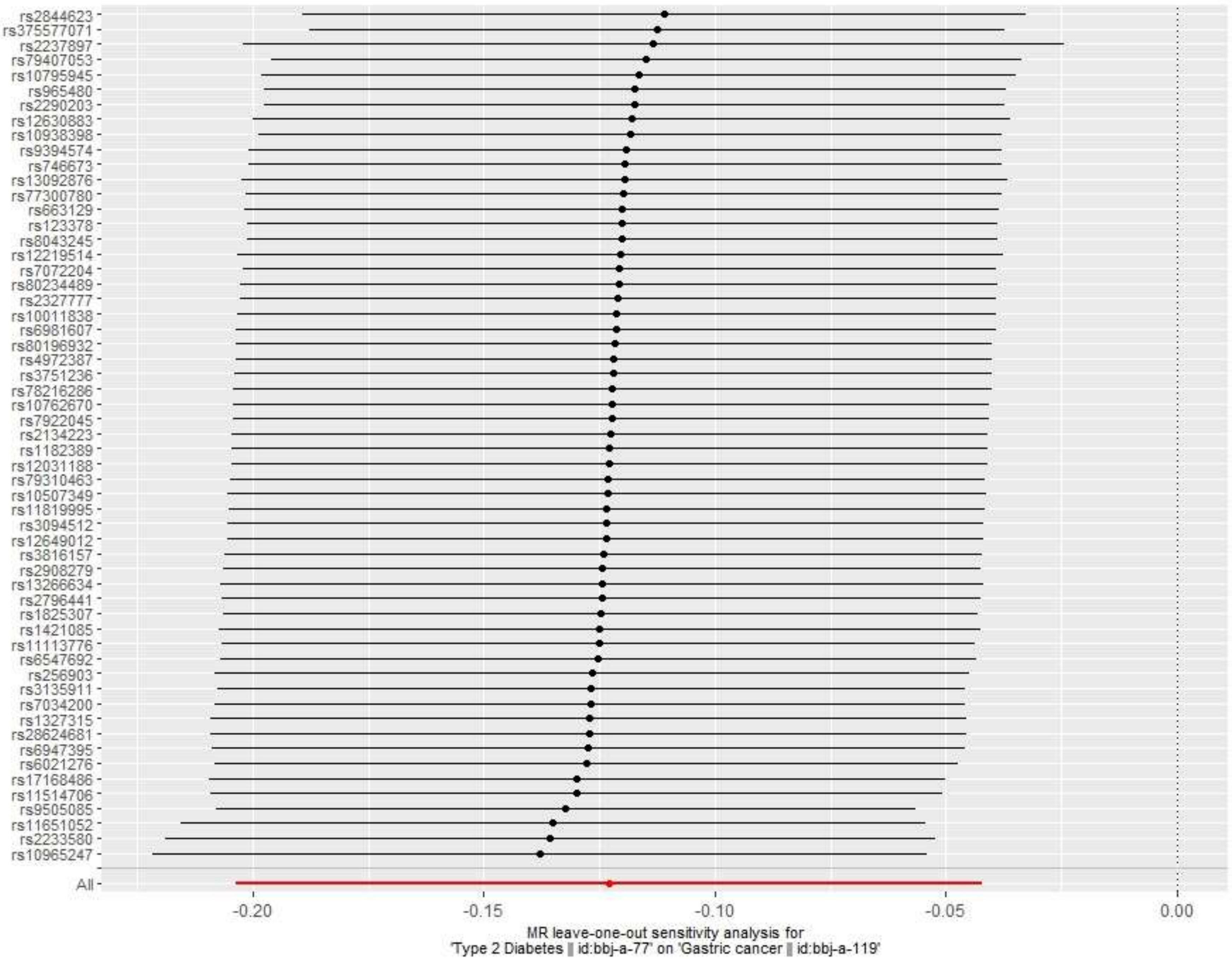
rs2844623
rs375577071
rs2237897
rs79407053
rs10795945
rs965480
rs2290203
rs12630883
rs10938398
rs9394574
rs746673
rs13092876
rs77300780
rs663129
rs123378
rs8043245
rs12219514
rs7072204
rs80234489
rs2327777
rs10011838
rs6981607
rs80196932
rs4972387
rs3751236
rs78216286
rs10762670
rs7922045
rs2134223
rs1182389
rs12031188
rs79310463
rs10507349
rs11819995
rs3094512
rs12649012
rs3816157
rs2908279
rs13266634
rs2796441
rs1825307
rs1421085
rs11113776
rs6547692
rs256903
rs3135911
rs7034200
rs1327315
rs28624681
rs6947395
rs6021276
rs17168486
rs11514706
rs9505085
rs11651052
rs2233580
rs10965247
All
-0.20
-0.15
-0.10
-0.05
0.00
MR leave-one-out sensitivity analysis for
'Type 2 Diabetes || id:bbj-a-77' on 'Gastric cancer || id:bbj-a-119'

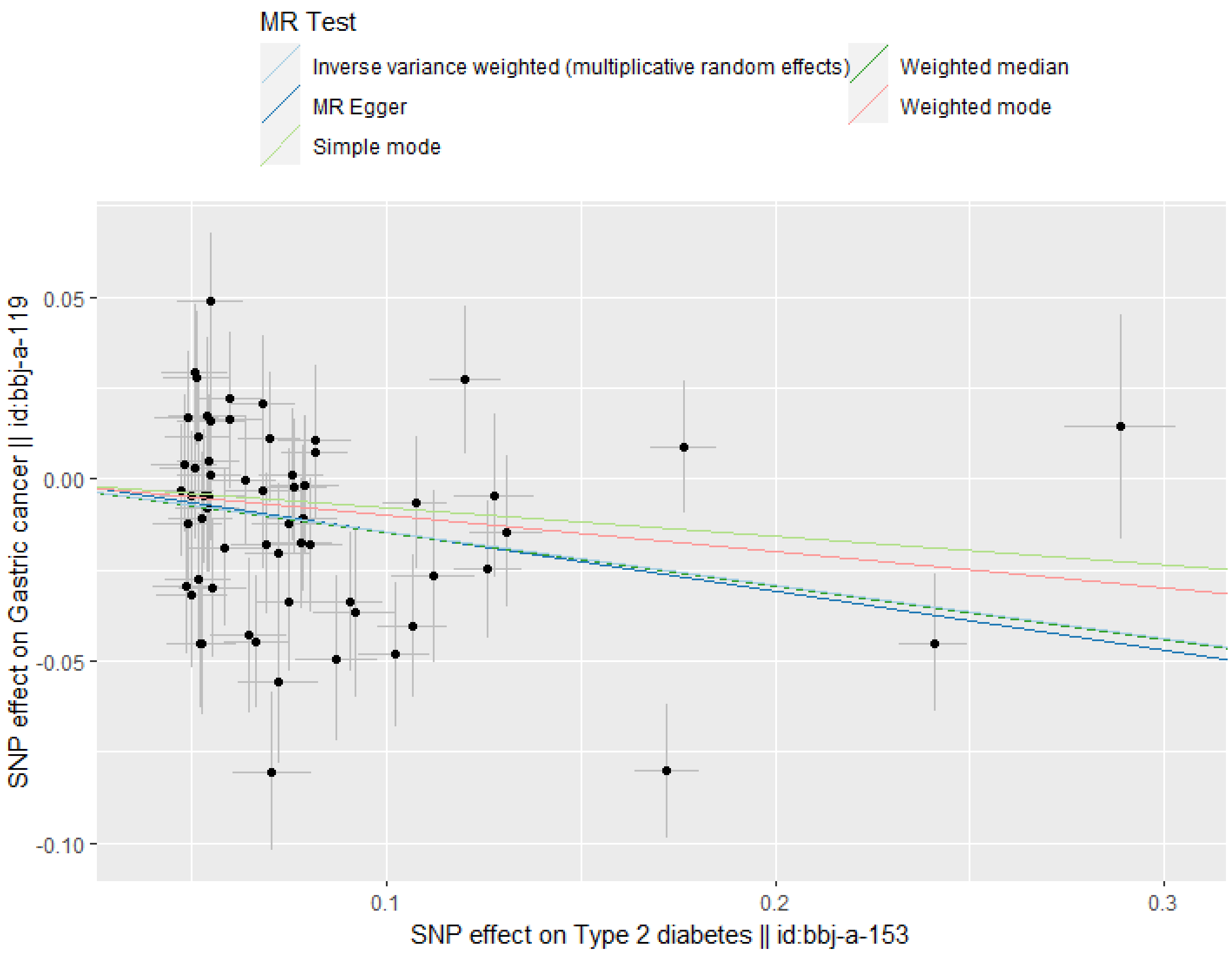

MR Test
Inverse variance weighted (multiplicative random effects)
MR Egger
Simple mode
Weighted median
Weighted mode
0.05
0.00
-0.05
-0.10
0.1
0.2
0.3
SNP effect on Gastric cancer || id:bbj-a-119
SNP effect on Type 2 diabetes || id:bbj-a-153

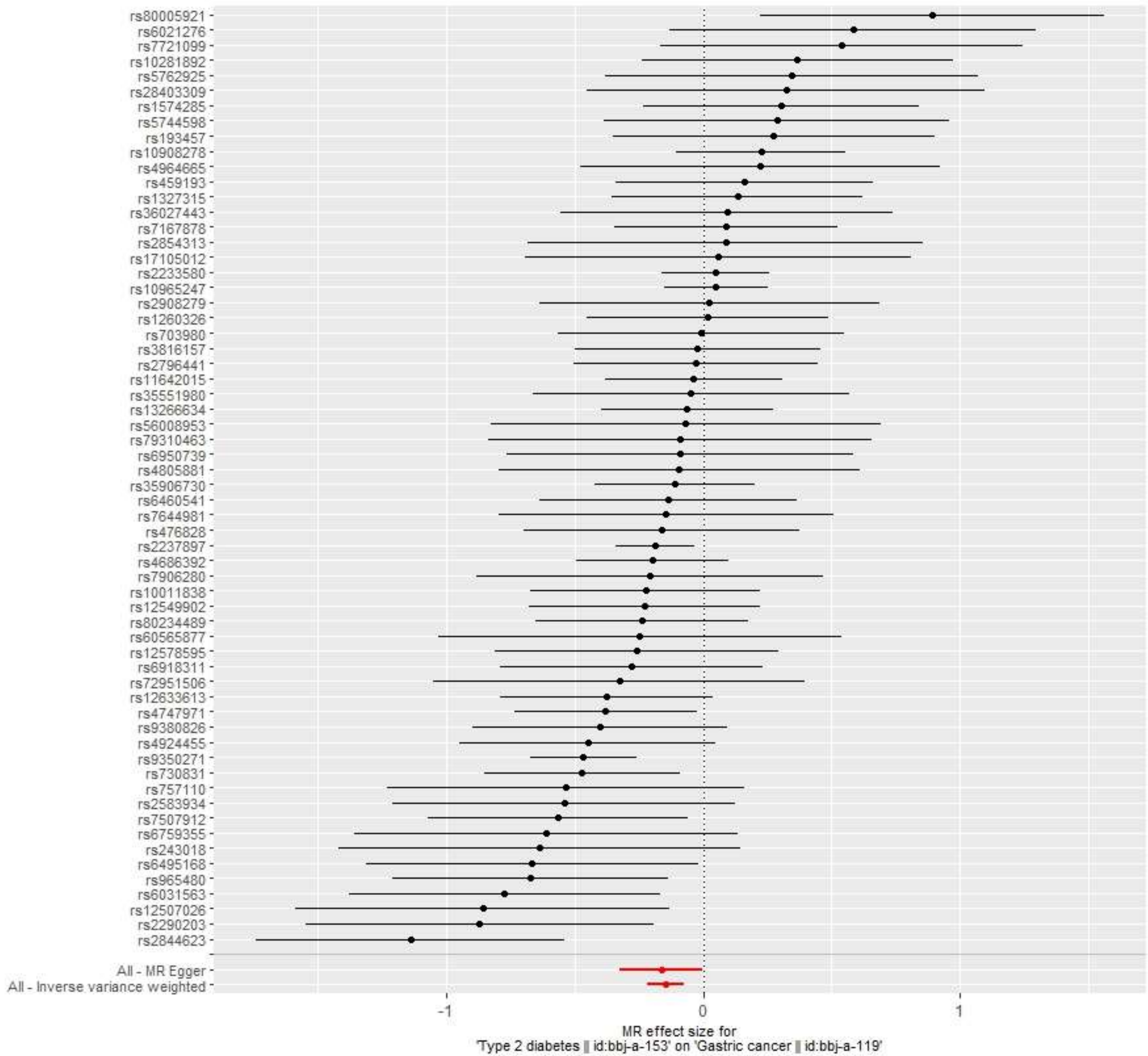
rs80005921
rs6021276
rs7721099
rs10281892
rs5762925
rs28403309
rs1574285
rs5744598
rs193457
rs10908278
rs4964665
rs459193
rs1327315
rs36027443
rs7167878
rs2854313
rs17105012
rs2233580
rs10965247
rs2908279
rs1260326
rs703980
rs3816157
rs2796441
rs11642015
rs35551980
rs13266634
rs56008953
rs79310463
rs6950739
rs4805881
rs35906730
rs6460541
rs7644981
rs476828
rs2237897
rs4686392
rs7906280
rs10011838
rs12549902
rs80234489
rs60565877
rs12578595
rs6918311
rs72951506
rs12633613
rs4747971
rs9380826
rs4924455
rs9350271
rs730831
rs757110
rs2583934
rs7507912
rs6759355
rs243018
rs6495168
rs965480
rs6031563
rs12507026
rs2290203
rs2844623
All - MR Egger
All - Inverse variance weighted
-1
0
1
MR effect size for
'Type 2 diabetes || id:bbj-a-153' on 'Gastric cancer || id:bbj-a-119'

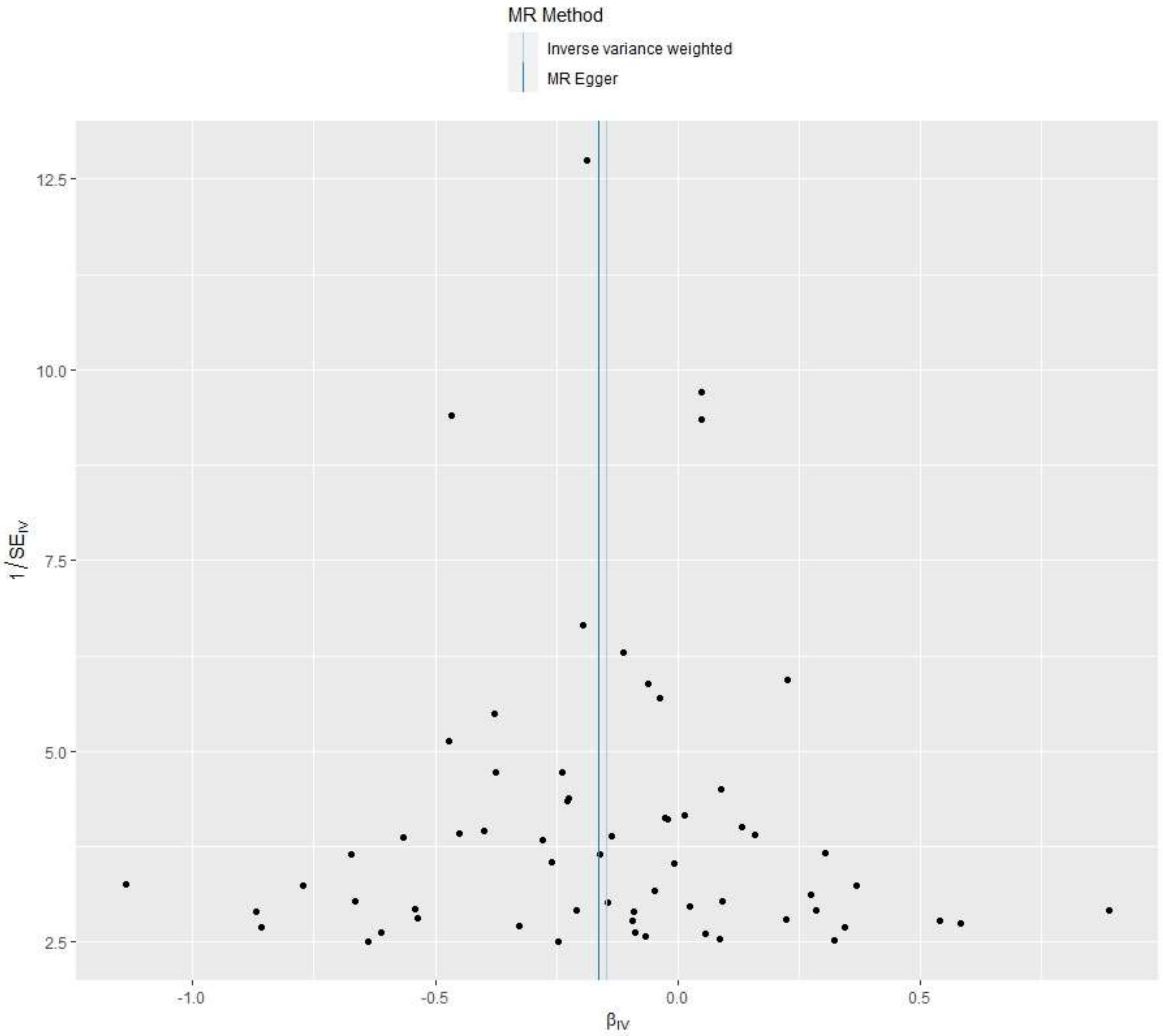
MR Method
Inverse variance weighted
MR Egger
12.5
10.0
7.5
5.0
2.5
1/SE_IV
-1.0
-0.5
0.0
0.5
β_IV

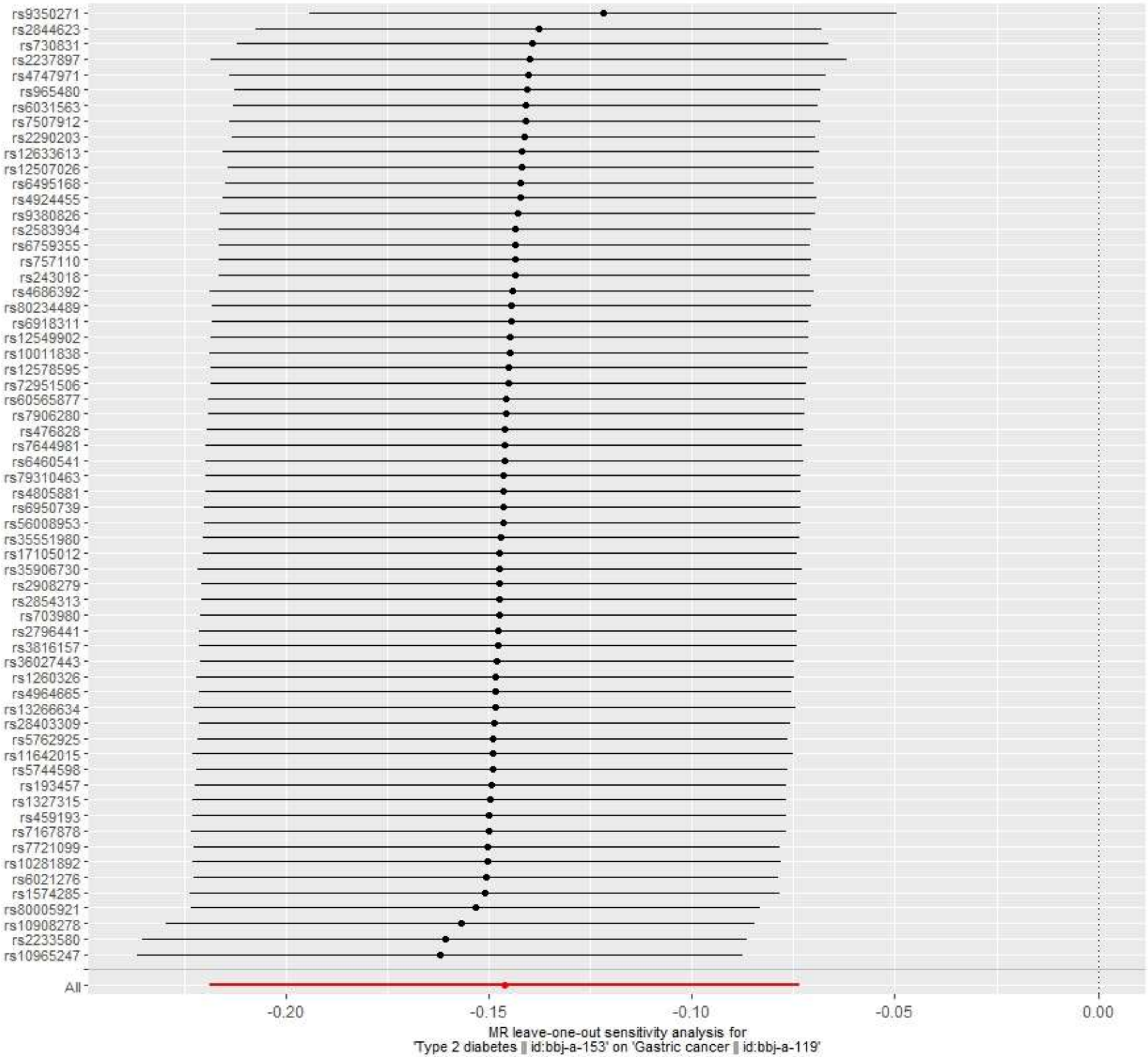
rs9350271
rs2844623
rs730831
rs2237897
rs4747971
rs965480
rs6031563
rs7507912
rs2290203
rs12633613
rs12507026
rs6495168
rs4924455
rs9380826
rs2583934
rs6759355
rs757110
rs243018
rs4686392
rs80234489
rs6918311
rs12549902
rs10011838
rs12578595
rs72951506
rs60565877
rs7906280
rs476828
rs7644981
rs6460541
rs79310463
rs4805881
rs6950739
rs56008953
rs35551980
rs17105012
rs35906730
rs2908279
rs2854313
rs703980
rs2796441
rs3816157
rs36027443
rs1260326
rs4964665
rs13266634
rs28403309
rs5762925
rs11642015
rs5744598
rs193457
rs1327315
rs459193
rs7167878
rs7721099
rs10281892
rs6021276
rs1574285
rs80005921
rs10908278
rs2233580
rs10965247
All
-0.20
-0.15
-0.10
-0.05
0.00
MR leave-one-out sensitivity analysis for
'Type 2 diabetes || id:bbj-a-153' on 'Gastric cancer || id:bbj-a-119'
