# Supplementary material for: T2DM may exert a protective effect against digestive system tumors in East Asian populations: a Mendelian randomization analysis
Source: Front Oncol. 2024 Jun 14;14:1327154. doi: 10.3389/fonc.2024.1327154 (PMC11211363; doi:10.3389/fonc.2024.1327154)
Supplement: Supplemental File 4 — The plot of MR result of T2DM on hepatocellular carcinoma in East Asian. [file DataSheet_4.pdf]

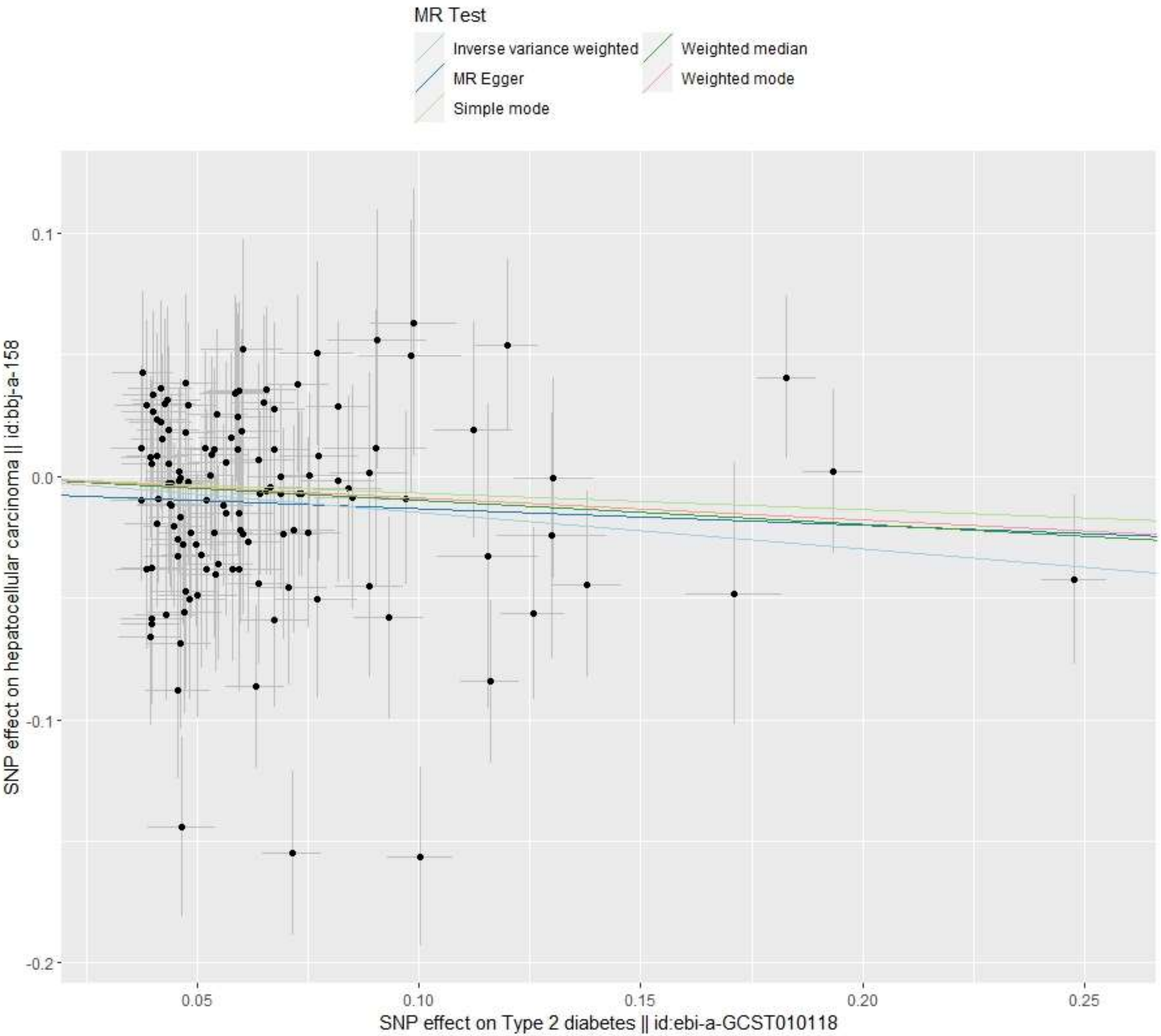
MR Test
Inverse variance weighted
MR Egger
Simple mode
Weighted median
Weighted mode
SNP effect on hepatocellular carcinoma || id:bbj-a-158
0.1
0.0
-0.1
-0.2
0.05
0.10
0.15
0.20
0.25
SNP effect on Type 2 diabetes || id:ebi-a-GCST010118

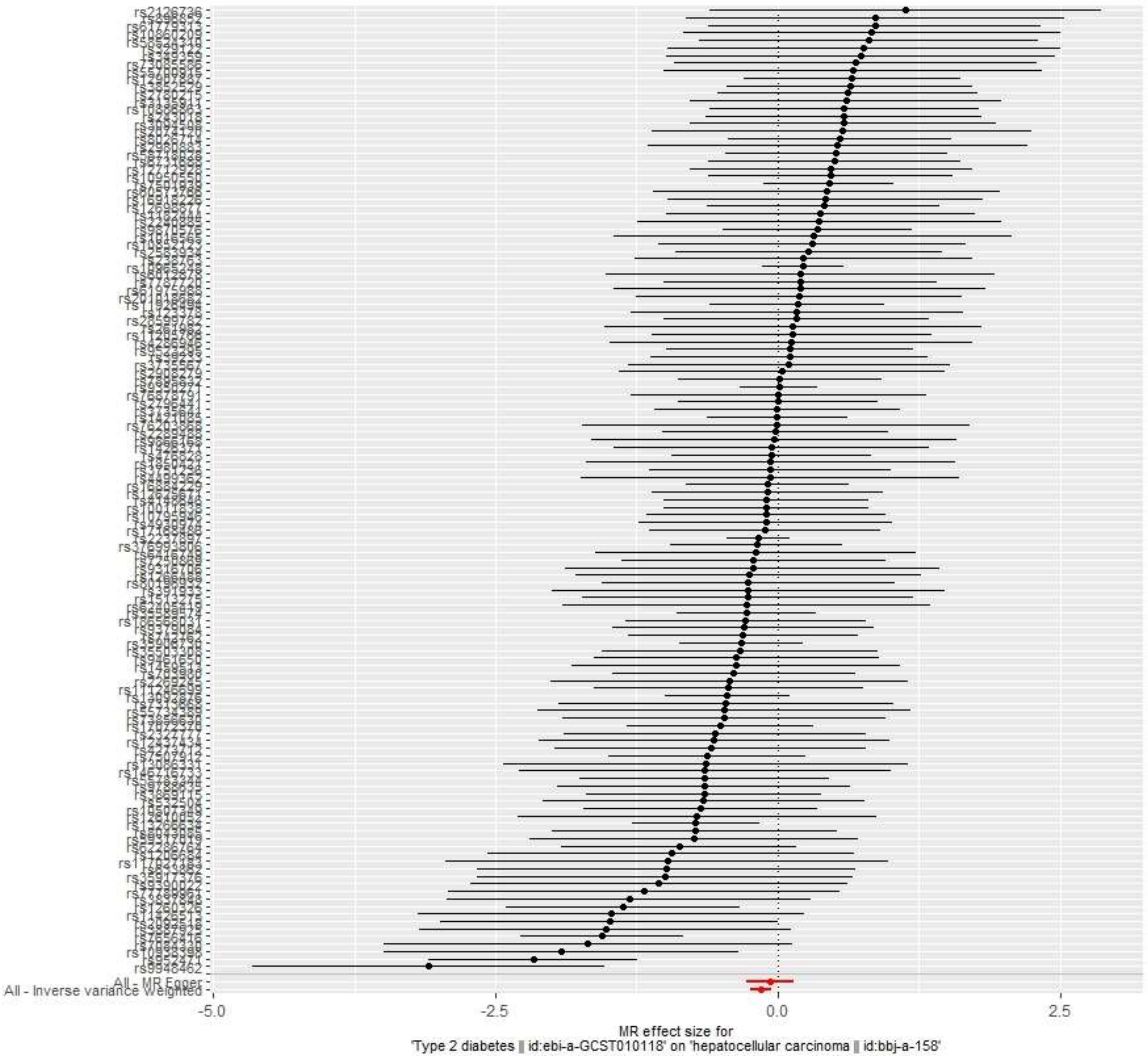

rs2126736
rs9946462
All - MR Egger
All - Inverse variance weighted
-5.0
-2.5
0.0
2.5
MR effect size for
'Type 2 diabetes || id:ebi-a-GCST010118' on 'hepatocellular carcinoma || id:bbj-a-158'

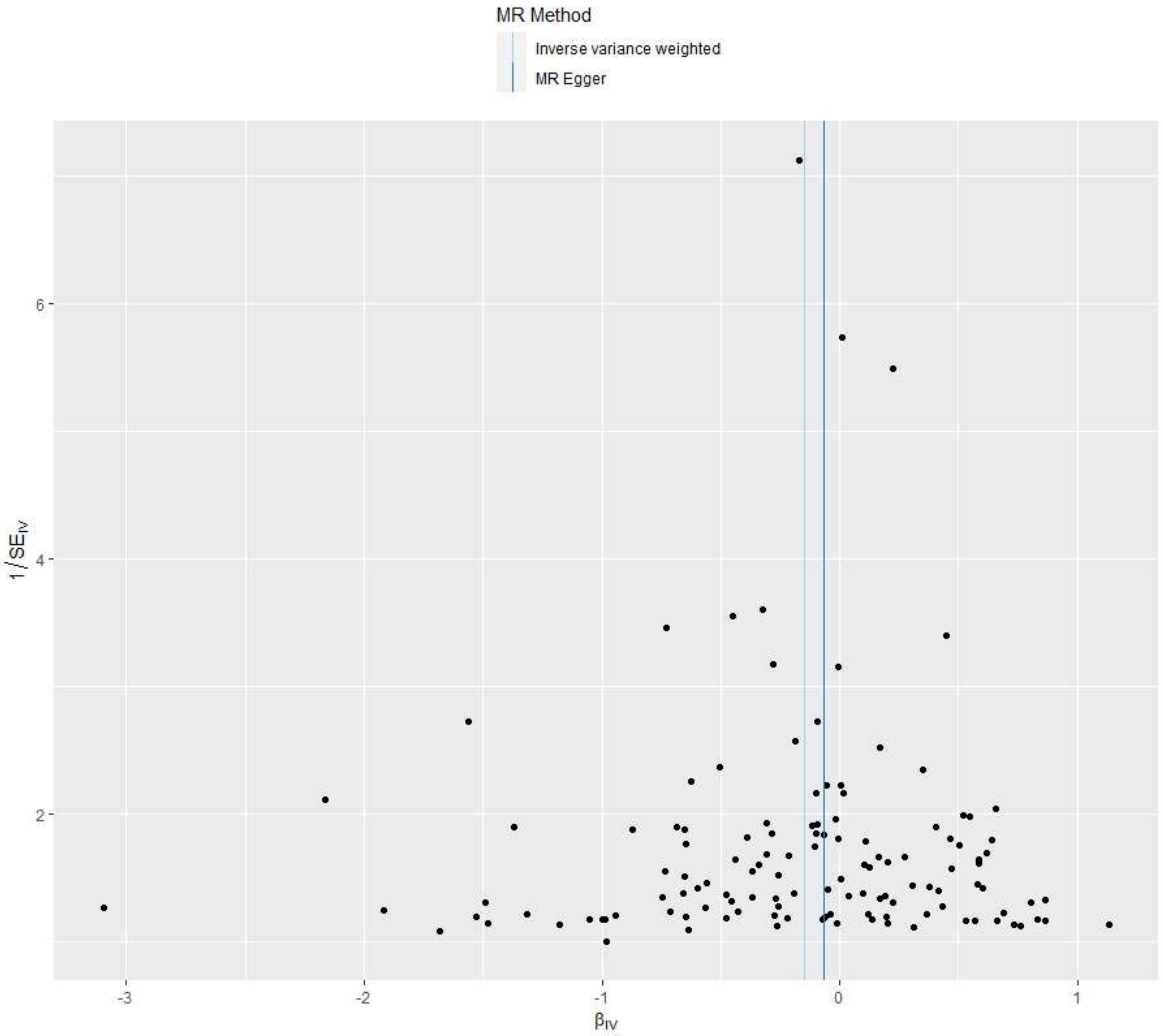
MR Method
Inverse variance weighted
MR Egger
6
4
2
-3
-2
-1
0
1
1/SE_IV
β_IV

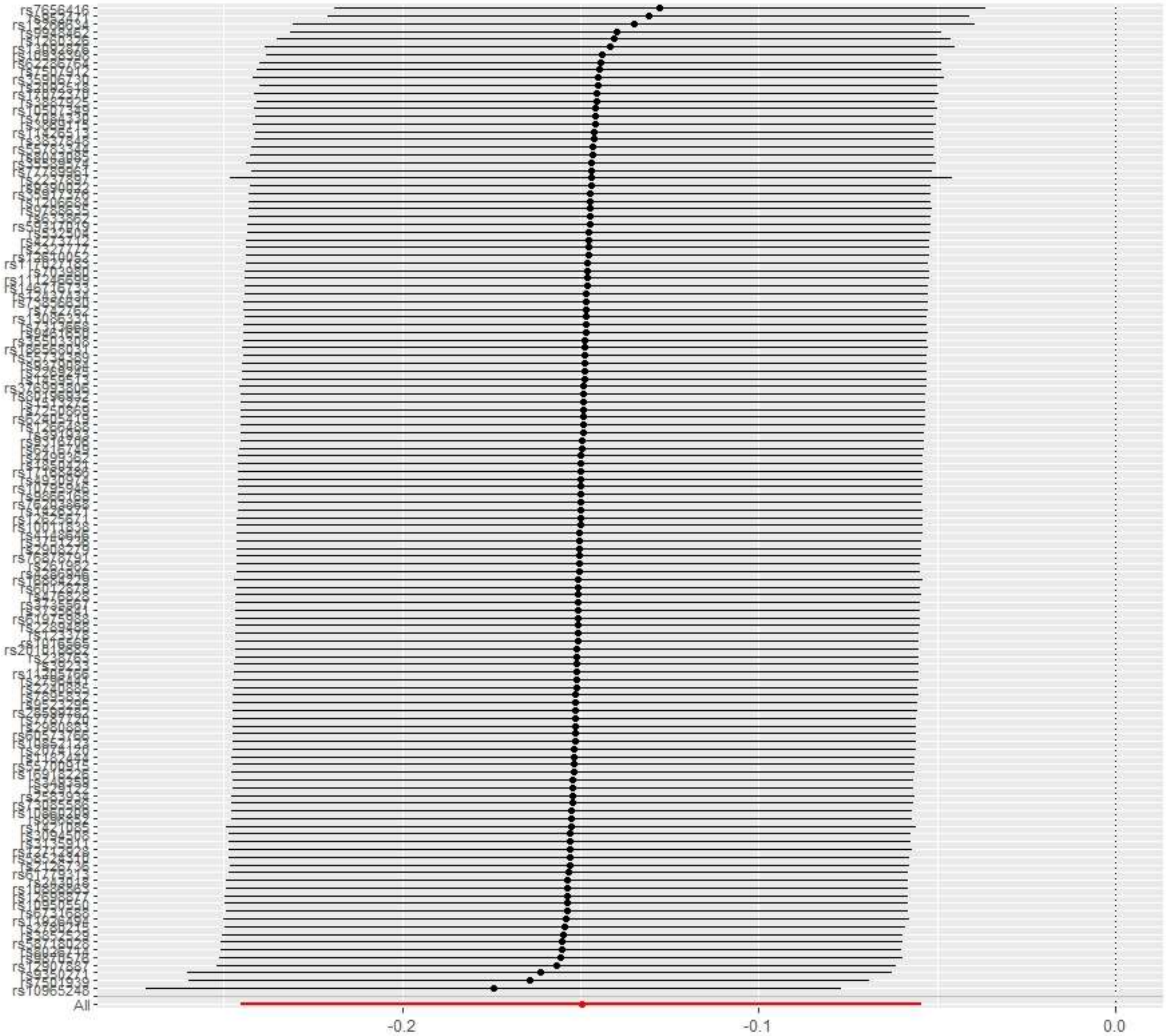

MR leave-one-out sensitivity analysis for
'Type 2 diabetes || id:ebi-a-GCST010118' on 'hepatocellular carcinoma || id:bbj-a-158'

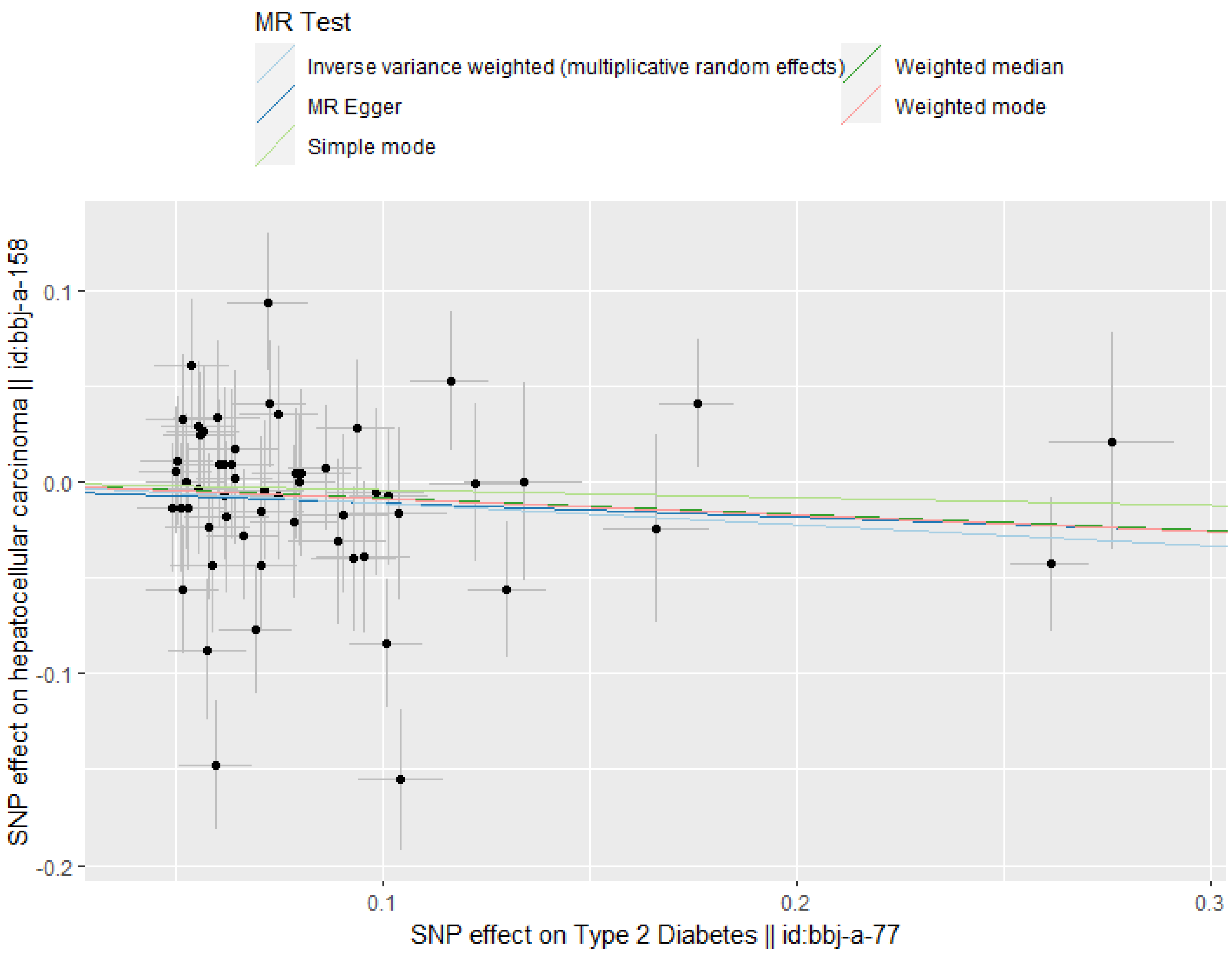
MR Test
Inverse variance weighted (multiplicative random effects)
MR Egger
Simple mode
Weighted median
Weighted mode
SNP effect on hepatocellular carcinoma || id:bbj-a-158
0.1
0.0
-0.1
-0.2
0.1
0.2
0.3
SNP effect on Type 2 Diabetes || id:bbj-a-77

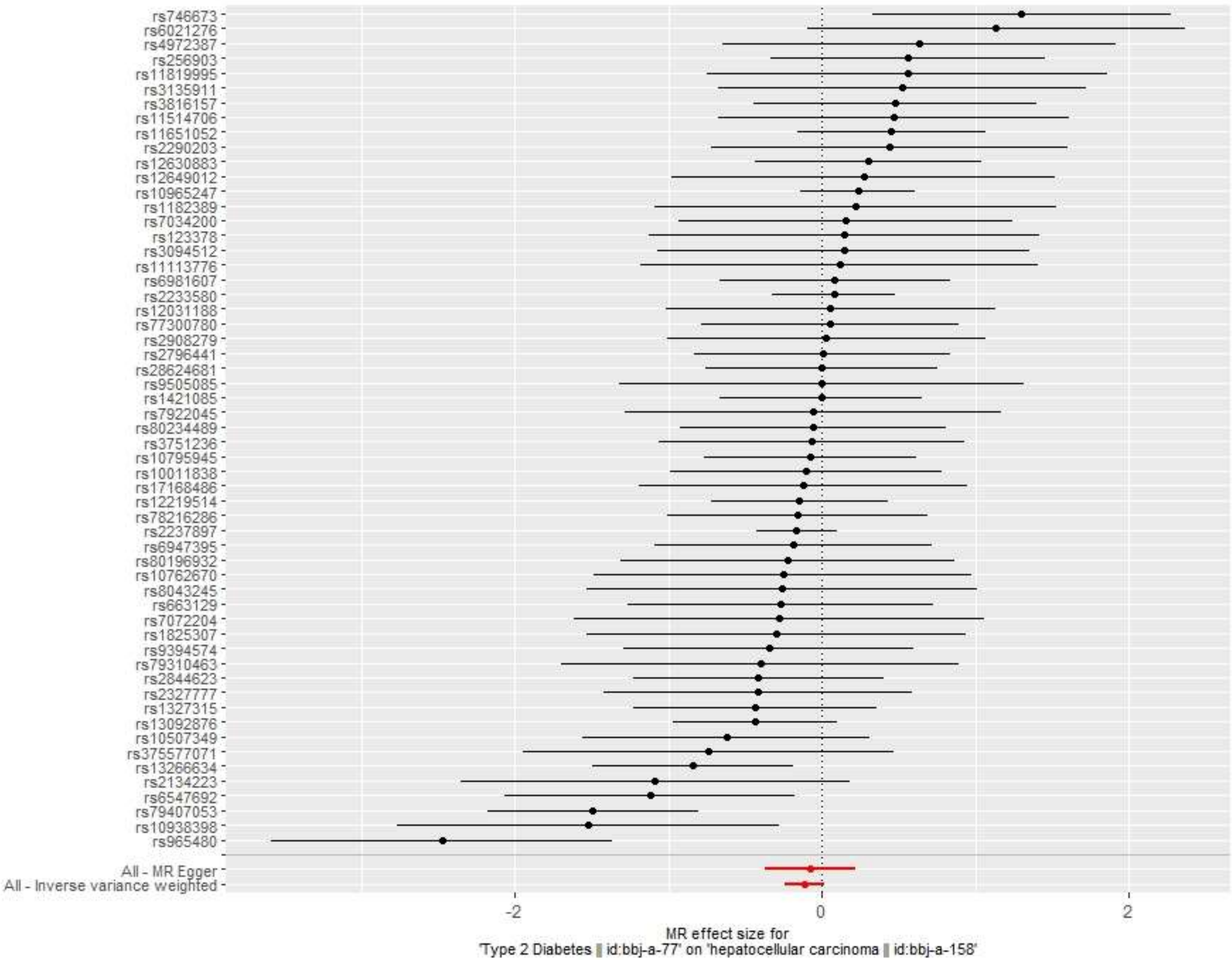
rs746673
rs6021276
rs4972387
rs256903
rs11819995
rs3135911
rs3816157
rs11514706
rs11651052
rs2290203
rs12630883
rs12649012
rs10965247
rs1182389
rs7034200
rs123378
rs3094512
rs11113776
rs6981607
rs2233580
rs12031188
rs77300780
rs2908279
rs2796441
rs28624681
rs9505085
rs1421085
rs7922045
rs80234489
rs3751236
rs10795945
rs10011838
rs17168486
rs12219514
rs78216286
rs2237897
rs6947395
rs80196932
rs10762670
rs8043245
rs663129
rs7072204
rs1825307
rs9394574
rs79310463
rs2844623
rs2327777
rs1327315
rs13092876
rs10507349
rs375577071
rs13266634
rs2134223
rs6547692
rs79407053
rs10938398
rs965480
All - MR Egger
All - Inverse variance weighted
-2
0
2
MR effect size for
'Type 2 Diabetes || id:bbj-a-77' on 'hepatocellular carcinoma || id:bbj-a-158'

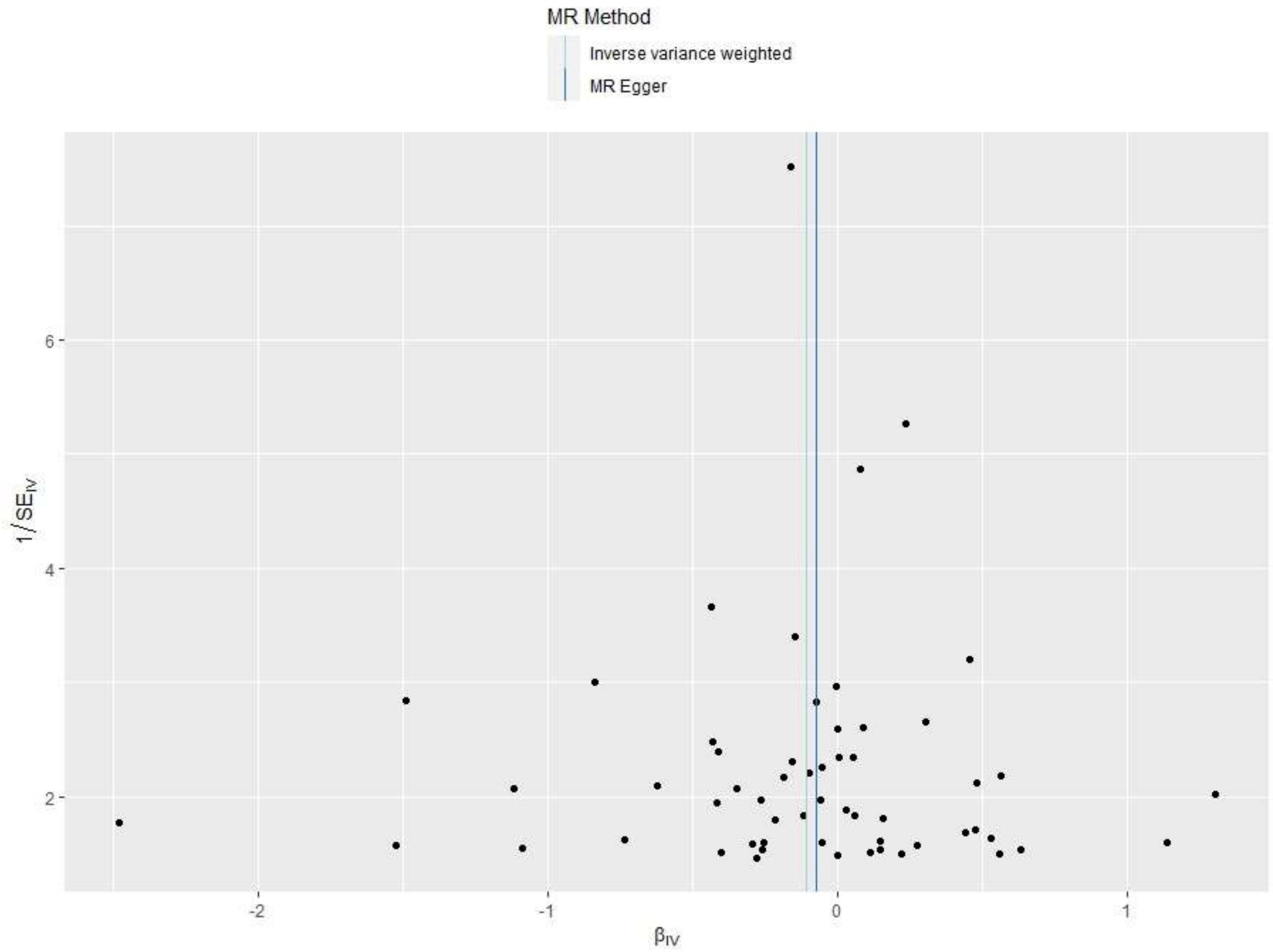
MR Method
Inverse variance weighted
MR Egger
6
4
2
1/SE_IV
-2
-1
0
1
β_IV

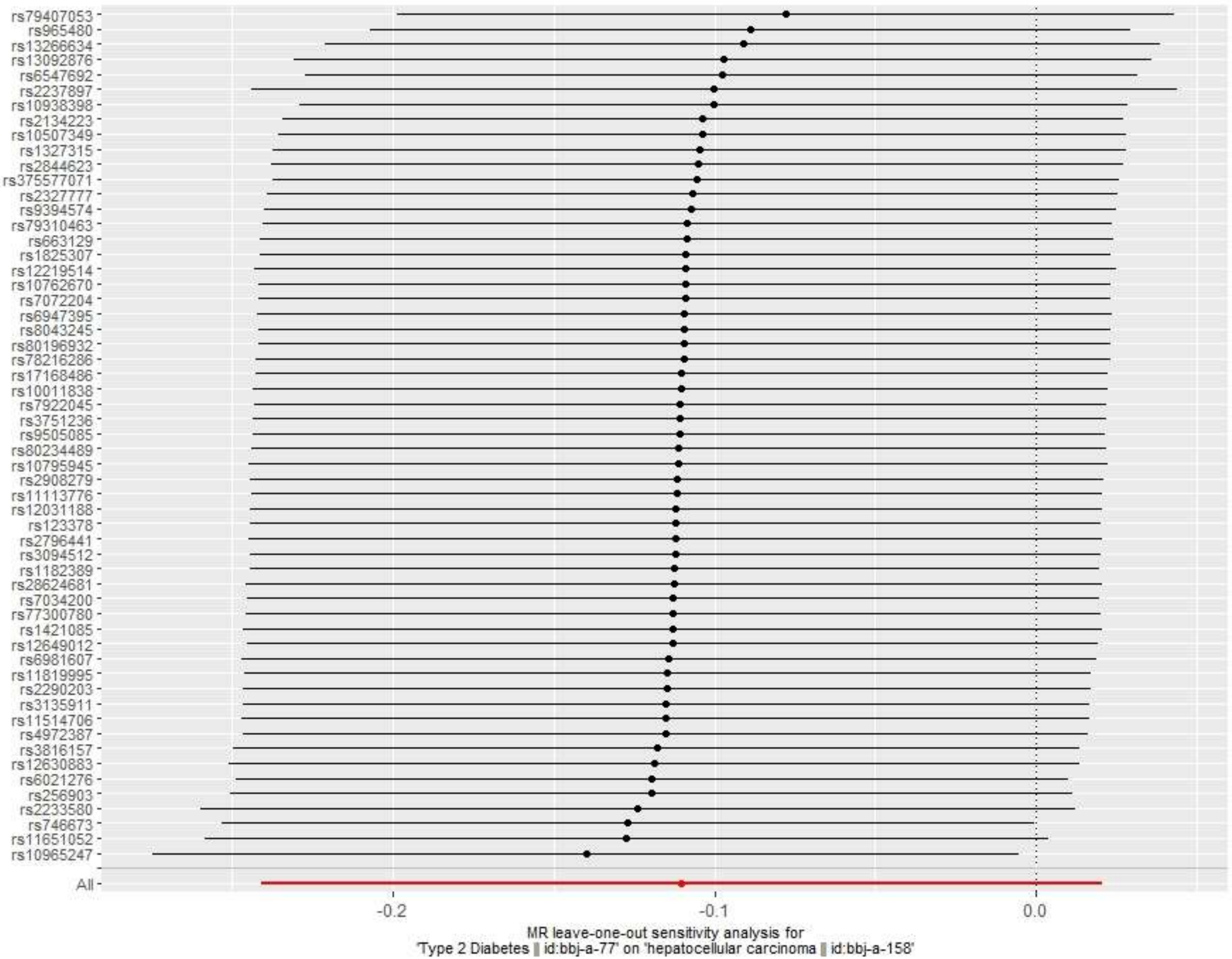
rs79407053
rs965480
rs13266634
rs13092876
rs6547692
rs2237897
rs10938398
rs2134223
rs10507349
rs1327315
rs2844623
rs375577071
rs2327777
rs9394574
rs79310463
rs663129
rs1825307
rs12219514
rs10762670
rs7072204
rs6947395
rs8043245
rs80196932
rs78216286
rs17168486
rs10011838
rs7922045
rs3751236
rs9505085
rs80234489
rs10795945
rs2908279
rs11113776
rs12031188
rs123378
rs2796441
rs3094512
rs1182389
rs28624681
rs7034200
rs77300780
rs1421085
rs12649012
rs6981607
rs11819995
rs2290203
rs3135911
rs11514706
rs4972387
rs3816157
rs12630883
rs6021276
rs256903
rs2233580
rs746673
rs11651052
rs10965247
All
-0.2
-0.1
0.0
MR leave-one-out sensitivity analysis for
'Type 2 Diabetes || id:bbj-a-77' on 'hepatocellular carcinoma || id:bbj-a-158'

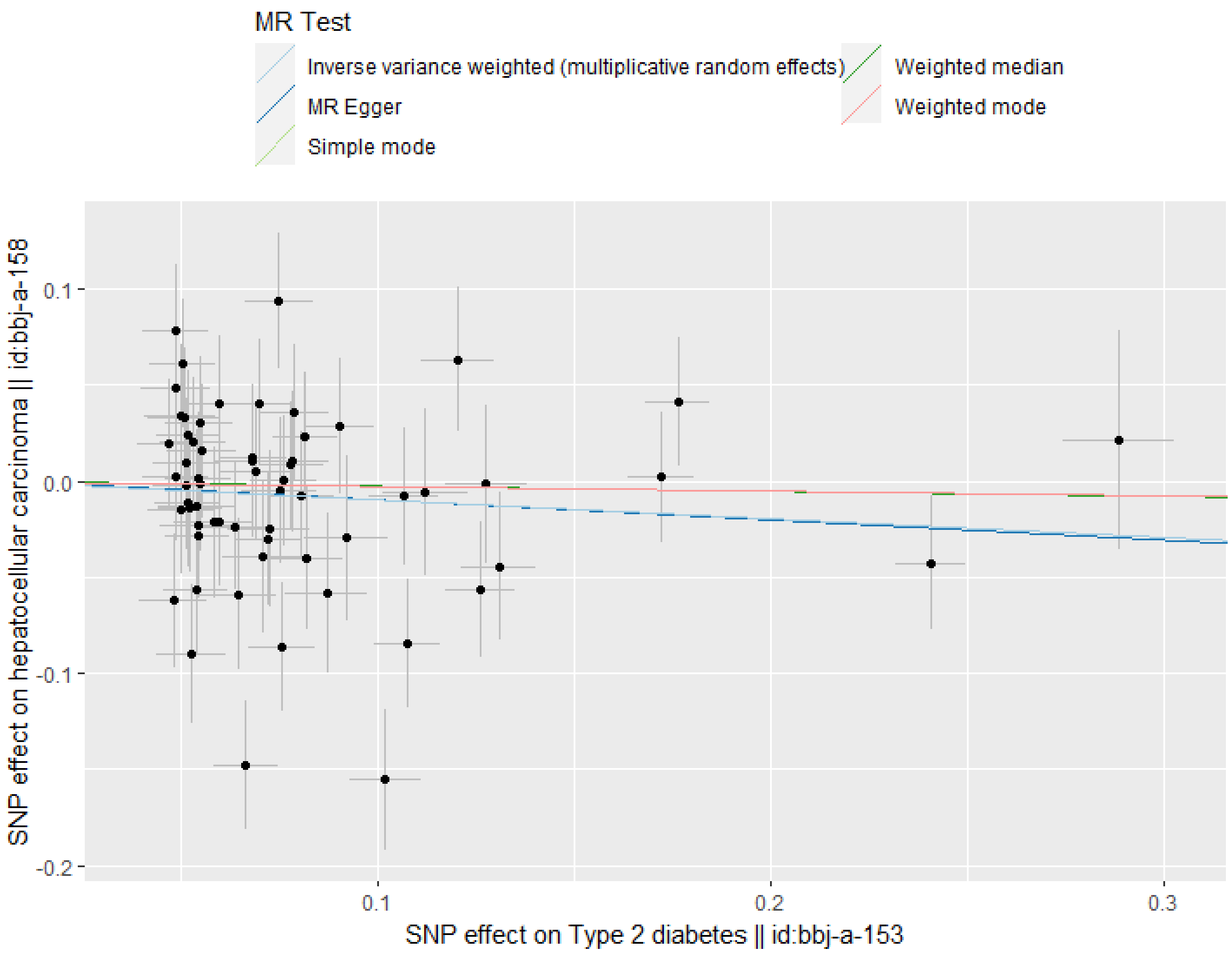

MR Test
Inverse variance weighted (multiplicative random effects)
MR Egger
Simple mode
Weighted median
Weighted mode
SNP effect on hepatocellular carcinoma || id:bbj-a-158
0.1
0.0
-0.1
-0.2
0.1
0.2
0.3
SNP effect on Type 2 diabetes || id:bbj-a-153

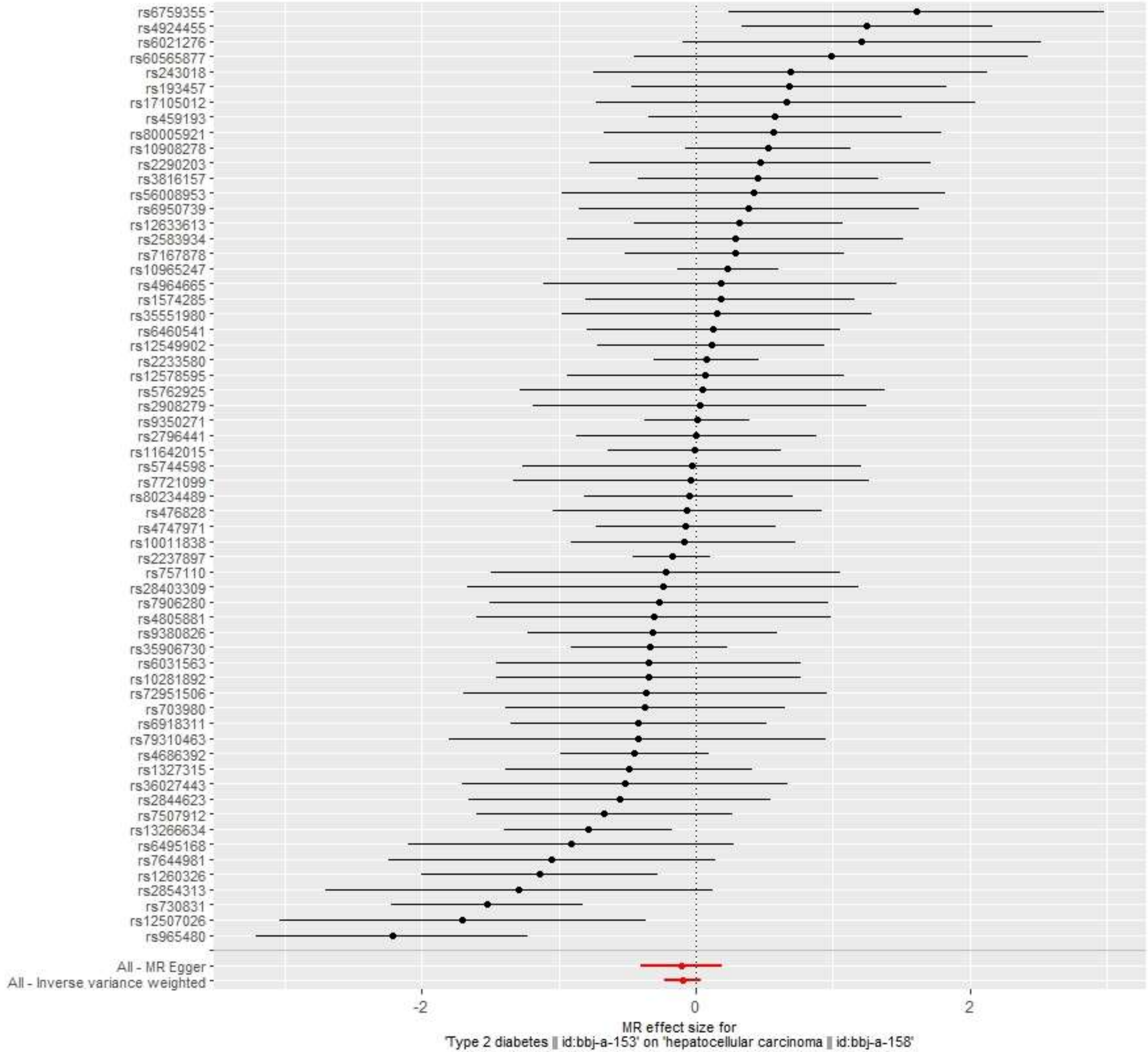
rs6759355
rs4924455
rs6021276
rs60565877
rs243018
rs193457
rs17105012
rs459193
rs80005921
rs10908278
rs2290203
rs3816157
rs56008953
rs6950739
rs12633613
rs2583934
rs7167878
rs10965247
rs4964665
rs1574285
rs35551980
rs6460541
rs12549902
rs2233580
rs12578595
rs5762925
rs2908279
rs9350271
rs2796441
rs11642015
rs5744598
rs7721099
rs80234489
rs476828
rs4747971
rs10011838
rs2237897
rs757110
rs28403309
rs7906280
rs4805881
rs9380826
rs35906730
rs6031563
rs10281892
rs72951506
rs703980
rs6918311
rs79310463
rs4686392
rs1327315
rs36027443
rs2844623
rs7507912
rs13266634
rs6495168
rs7644981
rs1260326
rs2854313
rs730831
rs12507026
rs965480
All - MR Egger
All - Inverse variance weighted
-2
0
2
MR effect size for
'Type 2 diabetes || id:bbj-a-153' on 'hepatocellular carcinoma || id:bbj-a-158'

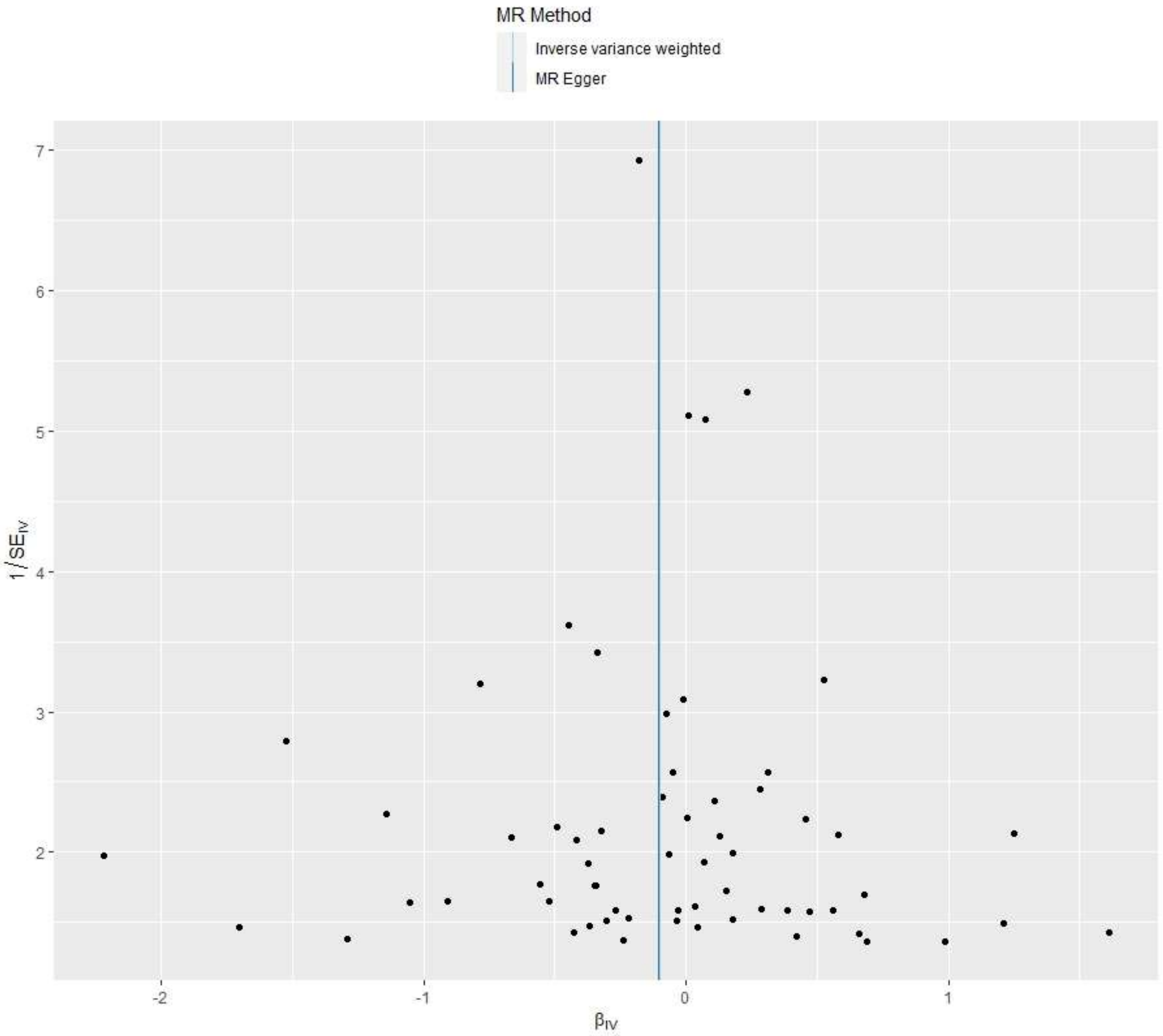
MR Method
Inverse variance weighted
MR Egger
7
6
5
4
3
2
-2
-1
0
1
1/SE$_{IV}$
β$_{IV}$

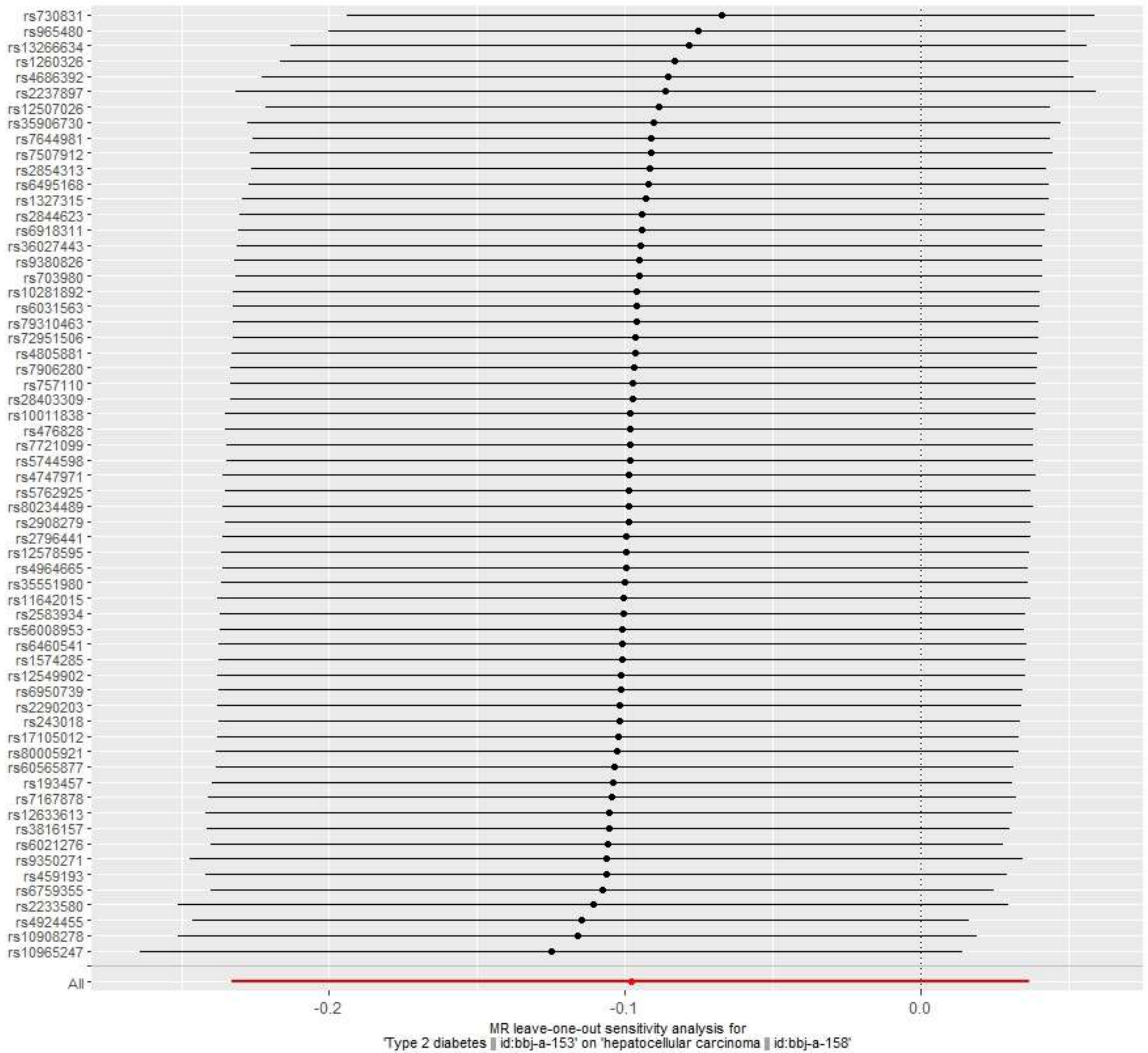
rs730831
rs965480
rs13266634
rs1260326
rs4686392
rs2237897
rs12507026
rs35906730
rs7644981
rs7507912
rs2854313
rs6495168
rs1327315
rs2844623
rs6918311
rs36027443
rs9380826
rs703980
rs10281892
rs6031563
rs79310463
rs72951506
rs4805881
rs7906280
rs757110
rs28403309
rs10011838
rs476828
rs7721099
rs5744598
rs4747971
rs5762925
rs80234489
rs2908279
rs2796441
rs12578595
rs4964665
rs35551980
rs11642015
rs2583934
rs56008953
rs6460541
rs1574285
rs12549902
rs6950739
rs2290203
rs243018
rs17105012
rs80005921
rs60565877
rs193457
rs7167878
rs12633613
rs3816157
rs6021276
rs9350271
rs459193
rs6759355
rs2233580
rs4924455
rs10908278
rs10965247
All
-0.2
-0.1
0.0
MR leave-one-out sensitivity analysis for
'Type 2 diabetes || id:bbj-a-153' on 'hepatocellular carcinoma || id:bbj-a-158'
