# Supplementary material for: T2DM may exert a protective effect against digestive system tumors in East Asian populations: a Mendelian randomization analysis
Source: Front Oncol. 2024 Jun 14;14:1327154. doi: 10.3389/fonc.2024.1327154 (PMC11211363; doi:10.3389/fonc.2024.1327154)
Supplement: Supplemental File 5 — The plot of MR result of T2DM on pancreatic cancer in East Asian. [file DataSheet_5.pdf]

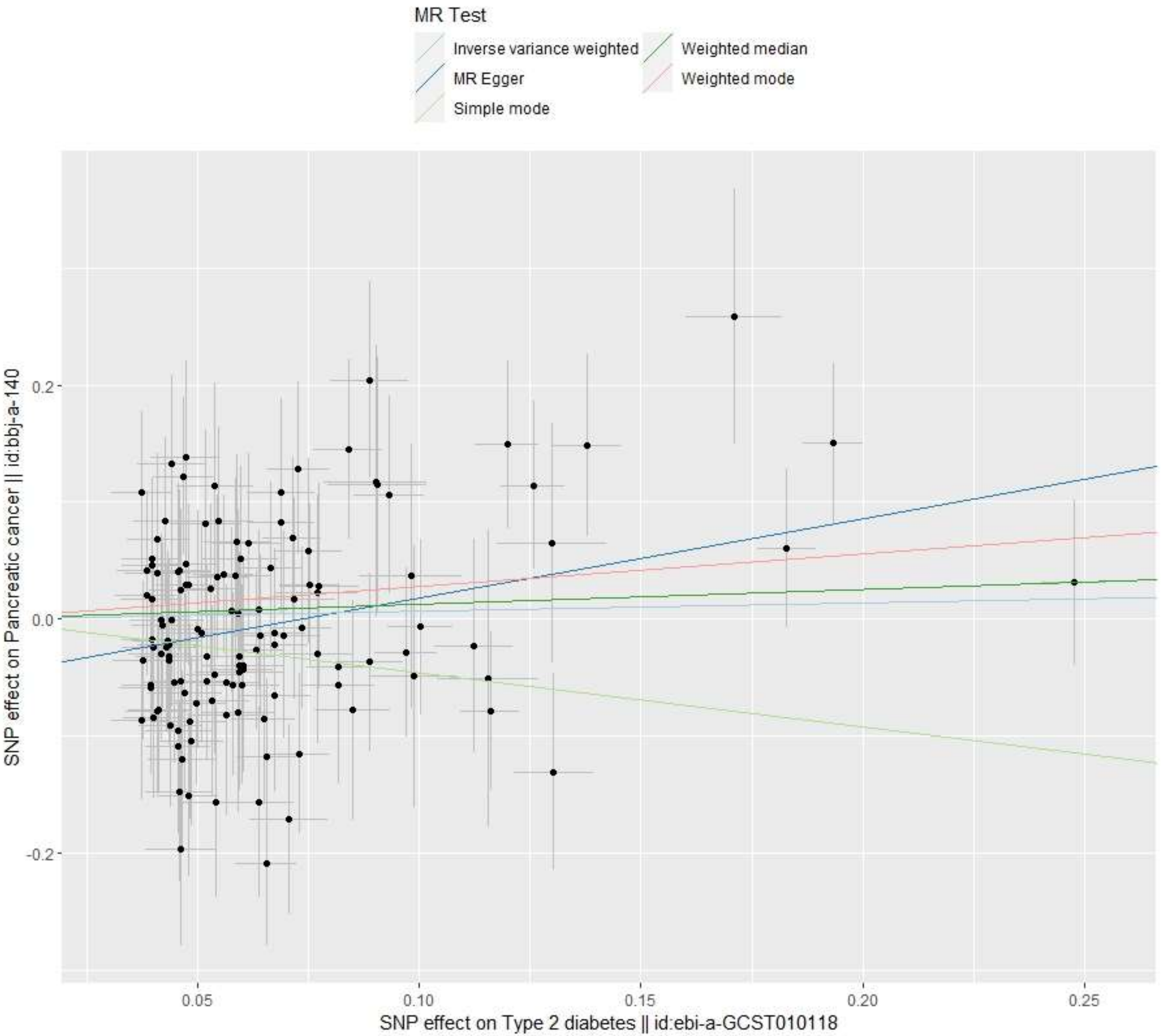
MR Test
Inverse variance weighted
MR Egger
Simple mode
Weighted median
Weighted mode
SNP effect on Pancreatic cancer || id:bbj-a-140
0.2
0.0
-0.2
0.05
0.10
0.15
0.20
0.25
SNP effect on Type 2 diabetes || id:ebi-a-GCST010118

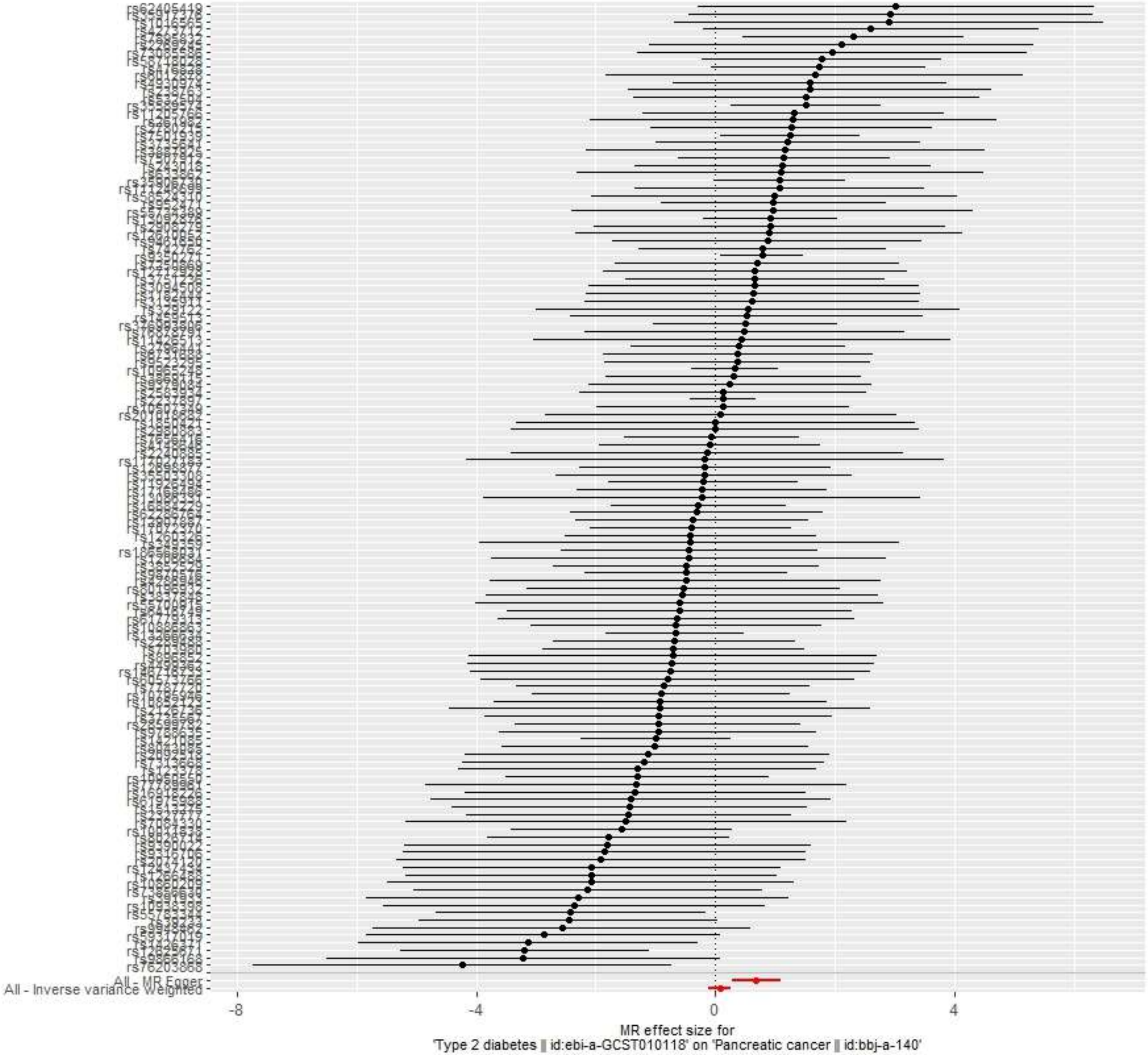
rs62405419
rs76203868
All - MR Egger
All - Inverse variance weighted
-8
-4
0
4
MR effect size for
'Type 2 diabetes || id:ebi-a-GCST010118' on 'Pancreatic cancer || id:bbj-a-140'

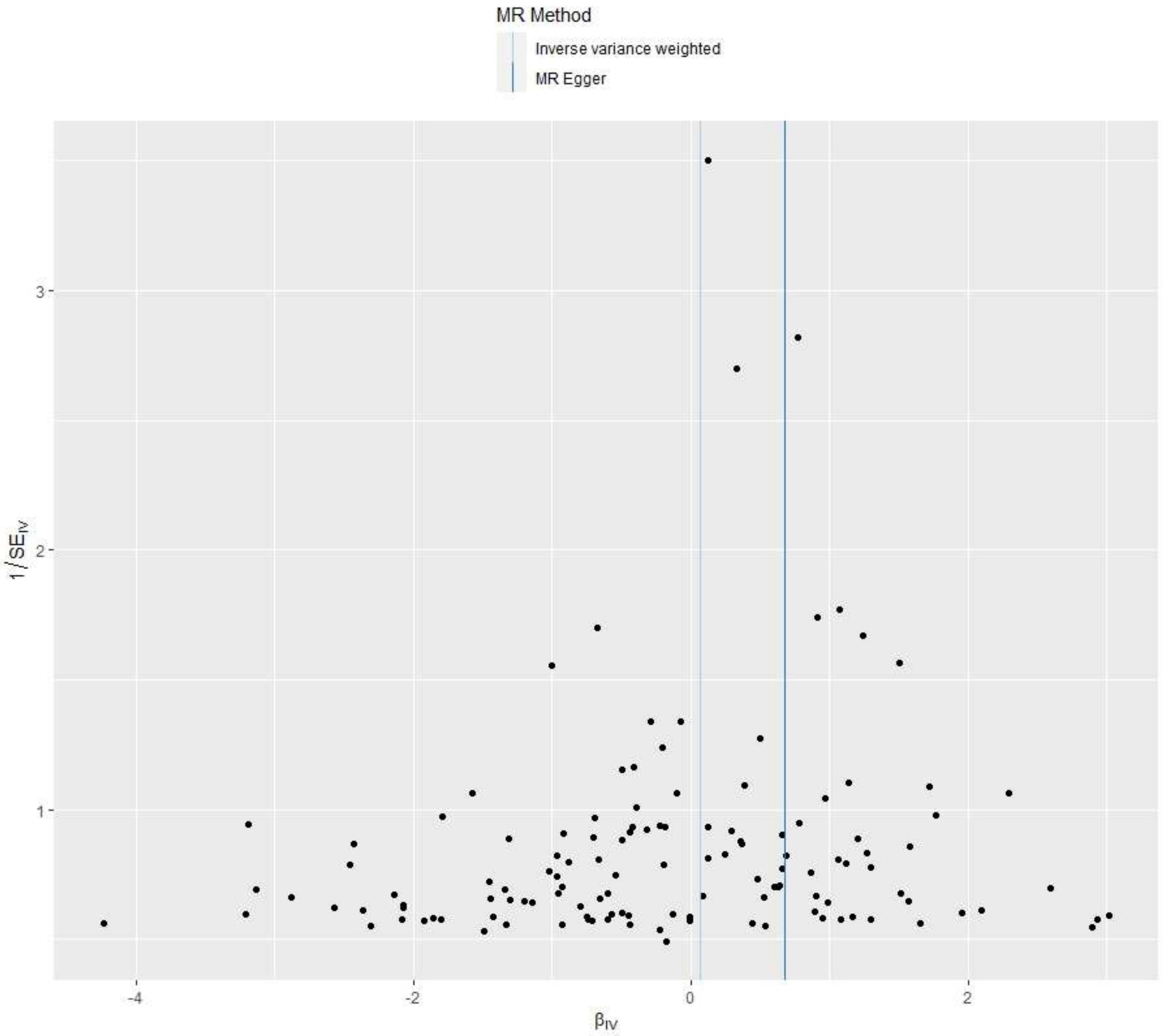
MR Method
Inverse variance weighted
MR Egger
3
2
1
$1/SE_{IV}$
-4
-2
0
2
β_{IV}

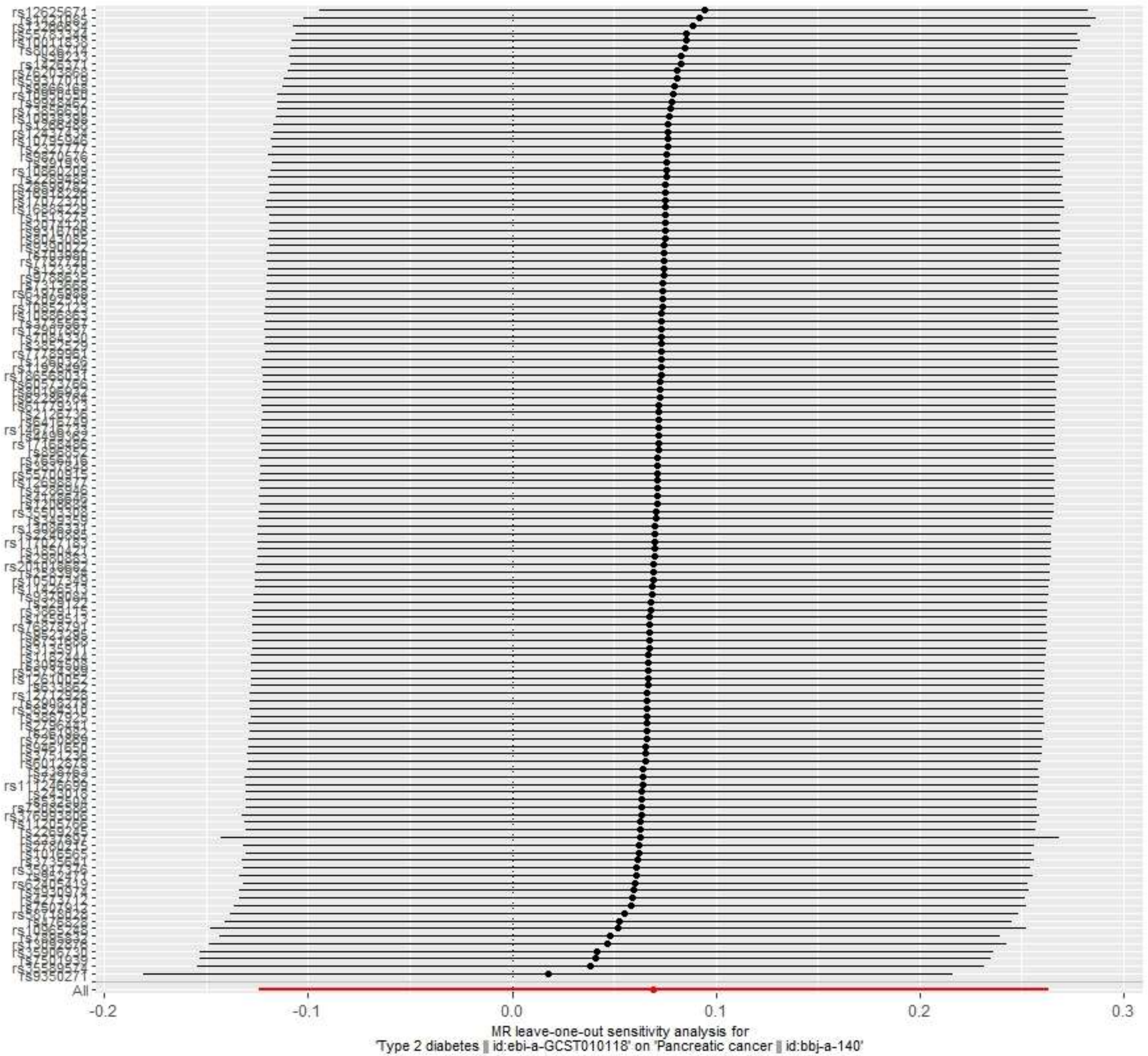
rs12625671
rs9350271
All
-0.2
-0.1
0.0
0.1
0.2
0.3
MR leave-one-out sensitivity analysis for
'Type 2 diabetes || id:ebi-a-GCST010118' on 'Pancreatic cancer || id:bbj-a-140'

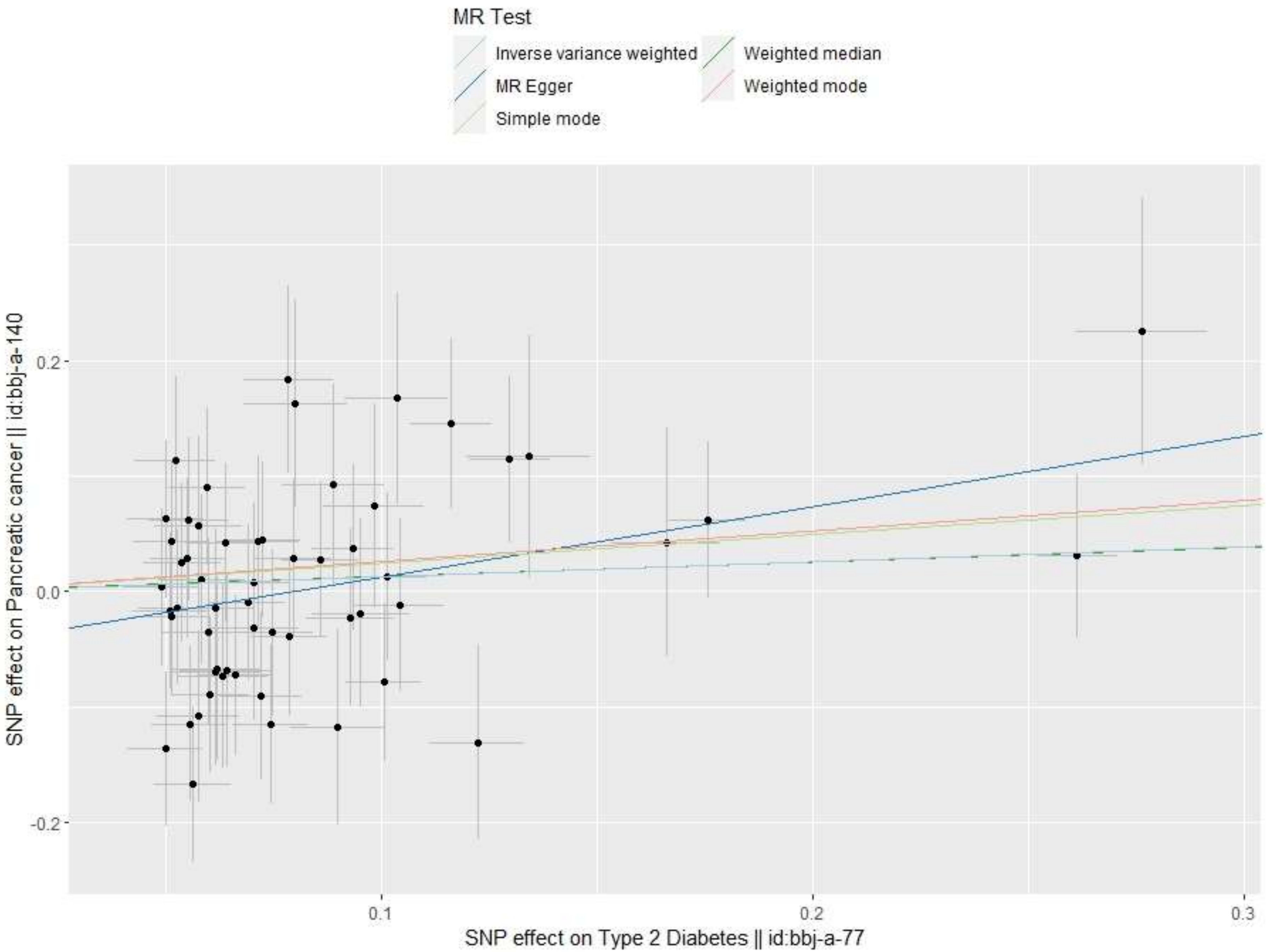
MR Test
Inverse variance weighted
MR Egger
Simple mode
Weighted median
Weighted mode
SNP effect on Pancreatic cancer || id:bbj-a-140
0.2
0.0
-0.2
0.1
0.2
0.3
SNP effect on Type 2 Diabetes || id:bbj-a-77

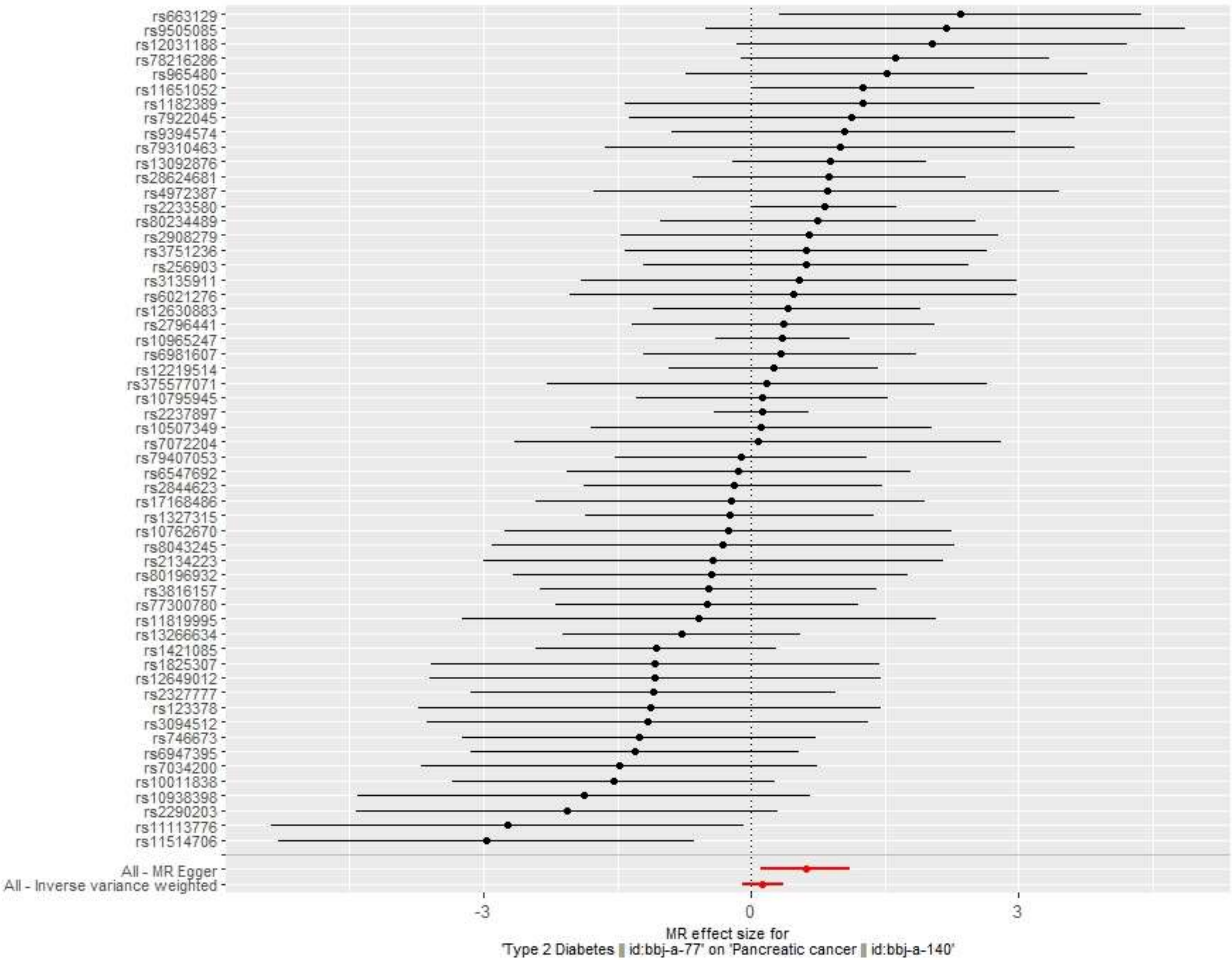
rs663129
rs9505085
rs12031188
rs78216286
rs965480
rs11651052
rs1182389
rs7922045
rs9394574
rs79310463
rs13092876
rs28624681
rs4972387
rs2233580
rs80234489
rs2908279
rs3751236
rs256903
rs3135911
rs6021276
rs12630883
rs2796441
rs10965247
rs6981607
rs12219514
rs375577071
rs10795945
rs2237897
rs10507349
rs7072204
rs79407053
rs6547692
rs2844623
rs17168486
rs1327315
rs10762670
rs8043245
rs2134223
rs80196932
rs3816157
rs77300780
rs11819995
rs13266634
rs1421085
rs1825307
rs12649012
rs2327777
rs123378
rs3094512
rs746673
rs6947395
rs7034200
rs10011838
rs10938398
rs2290203
rs11113776
rs11514706
All - MR Egger
All - Inverse variance weighted
-3
0
3
MR effect size for
'Type 2 Diabetes || id:bbj-a-77' on 'Pancreatic cancer || id:bbj-a-140'

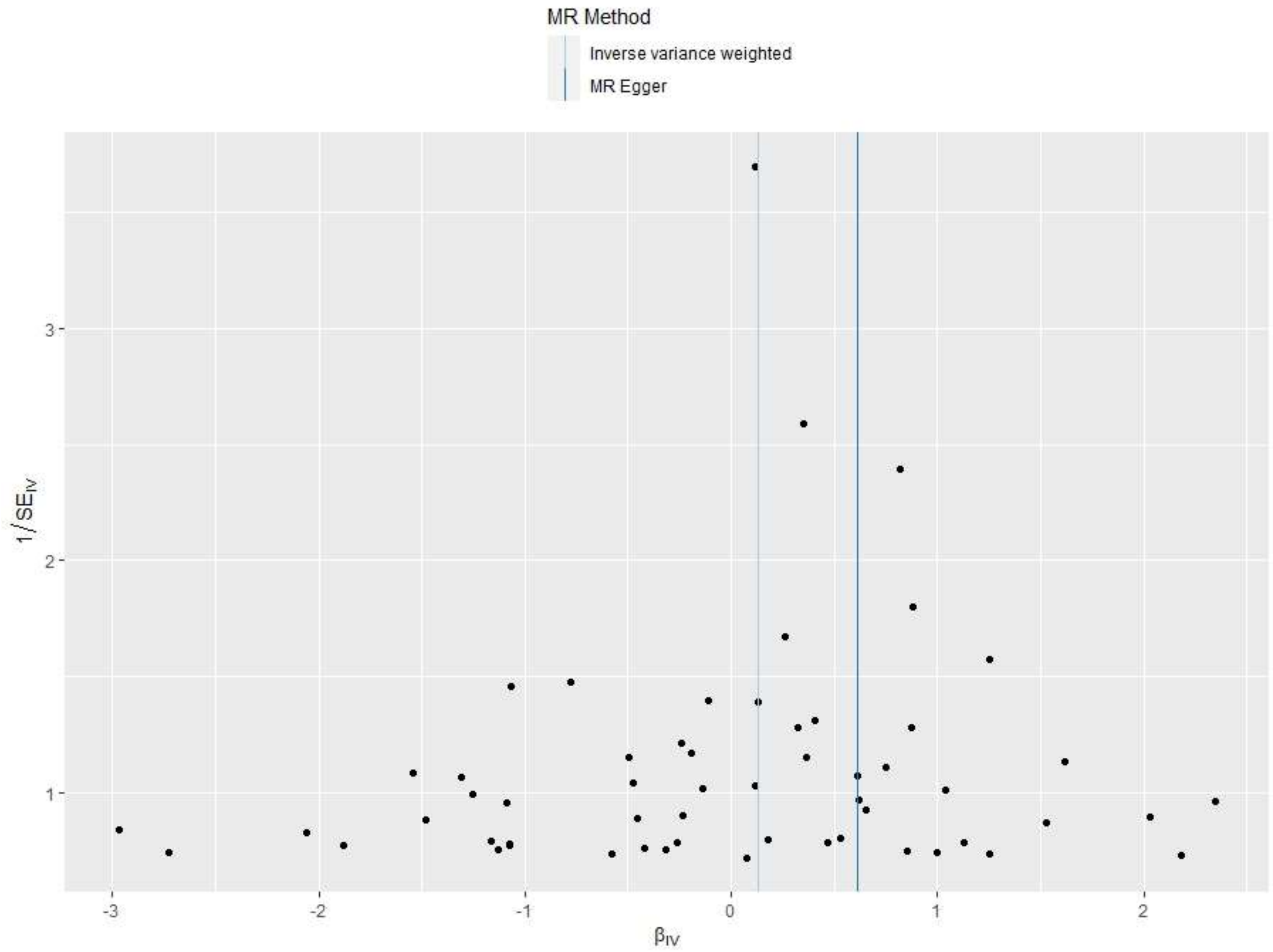
MR Method
Inverse variance weighted
MR Egger
1/SE_IV
β_IV
3
2
1
-3
-2
-1
0
1
2

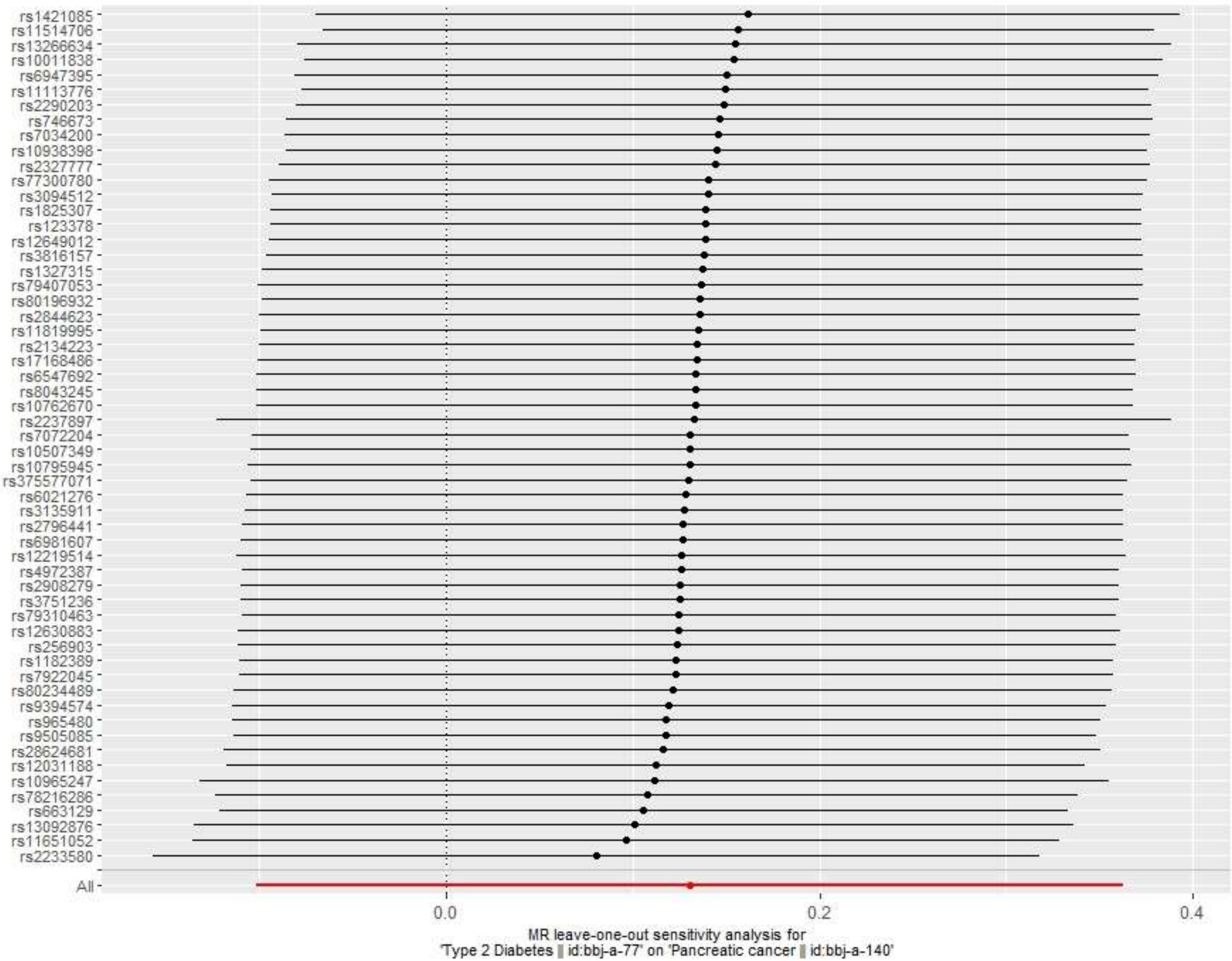
rs1421085
rs11514706
rs13266634
rs10011838
rs6947395
rs11113776
rs2290203
rs746673
rs7034200
rs10938398
rs2327777
rs77300780
rs3094512
rs1825307
rs123378
rs12649012
rs3816157
rs1327315
rs79407053
rs80196932
rs2844623
rs11819995
rs2134223
rs17168486
rs6547692
rs8043245
rs10762670
rs2237897
rs7072204
rs10507349
rs10795945
rs375577071
rs6021276
rs3135911
rs2796441
rs6981607
rs12219514
rs4972387
rs2908279
rs3751236
rs79310463
rs12630883
rs256903
rs1182389
rs7922045
rs80234489
rs9394574
rs965480
rs9505085
rs28624681
rs12031188
rs10965247
rs78216286
rs663129
rs13092876
rs11651052
rs2233580
All
0.0
0.2
0.4
MR leave-one-out sensitivity analysis for
'Type 2 Diabetes || id:bbj-a-77' on 'Pancreatic cancer || id:bbj-a-140'

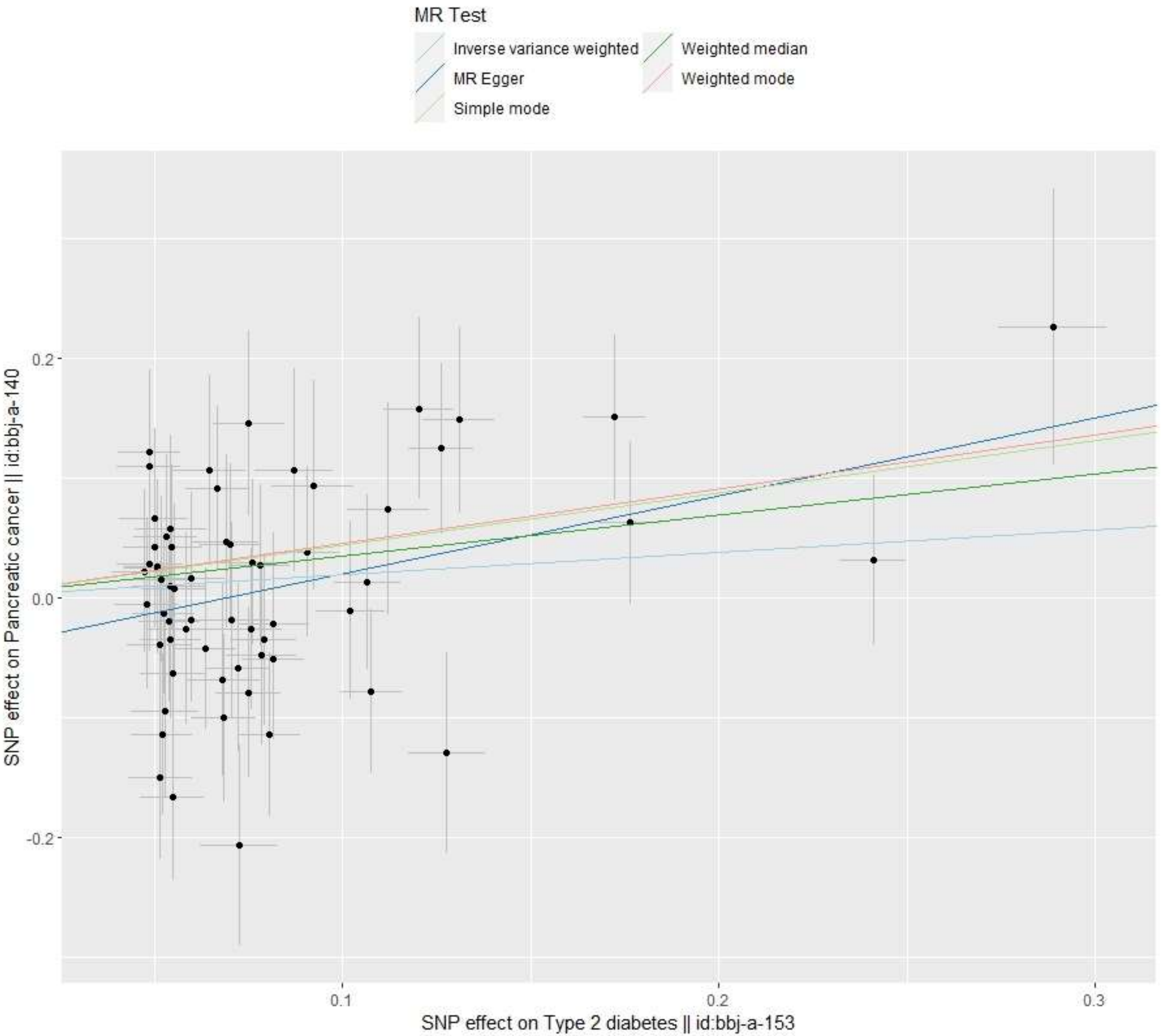
MR Test
Inverse variance weighted
MR Egger
Simple mode
Weighted median
Weighted mode
0.2
0.0
-0.2
0.1
0.2
0.3
SNP effect on Pancreatic cancer || id:bbj-a-140
SNP effect on Type 2 diabetes || id:bbj-a-153

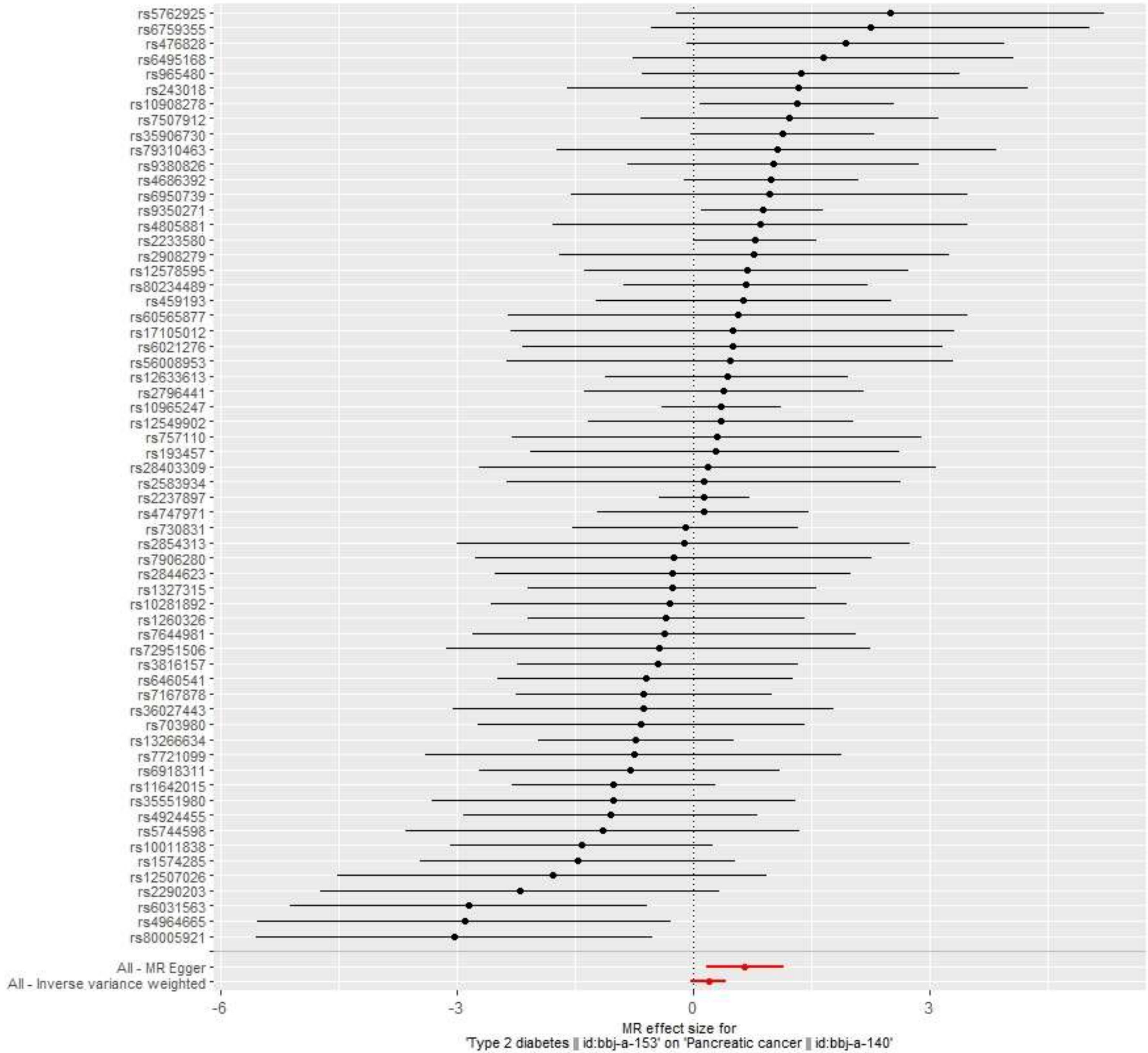
rs5762925
rs6759355
rs476828
rs6495168
rs965480
rs243018
rs10908278
rs7507912
rs35906730
rs79310463
rs9380826
rs4686392
rs6950739
rs9350271
rs4805881
rs2233580
rs2908279
rs12578595
rs80234489
rs459193
rs60565877
rs17105012
rs6021276
rs56008953
rs12633613
rs2796441
rs10965247
rs12549902
rs757110
rs193457
rs28403309
rs2583934
rs2237897
rs4747971
rs730831
rs2854313
rs7906280
rs2844623
rs1327315
rs10281892
rs1260326
rs7644981
rs72951506
rs3816157
rs6460541
rs7167878
rs36027443
rs703980
rs13266634
rs7721099
rs6918311
rs11642015
rs35551980
rs4924455
rs5744598
rs10011838
rs1574285
rs12507026
rs2290203
rs6031563
rs4964665
rs80005921
All - MR Egger
All - Inverse variance weighted
-6
-3
0
3
MR effect size for
'Type 2 diabetes || id:bbj-a-153' on 'Pancreatic cancer || id:bbj-a-140'

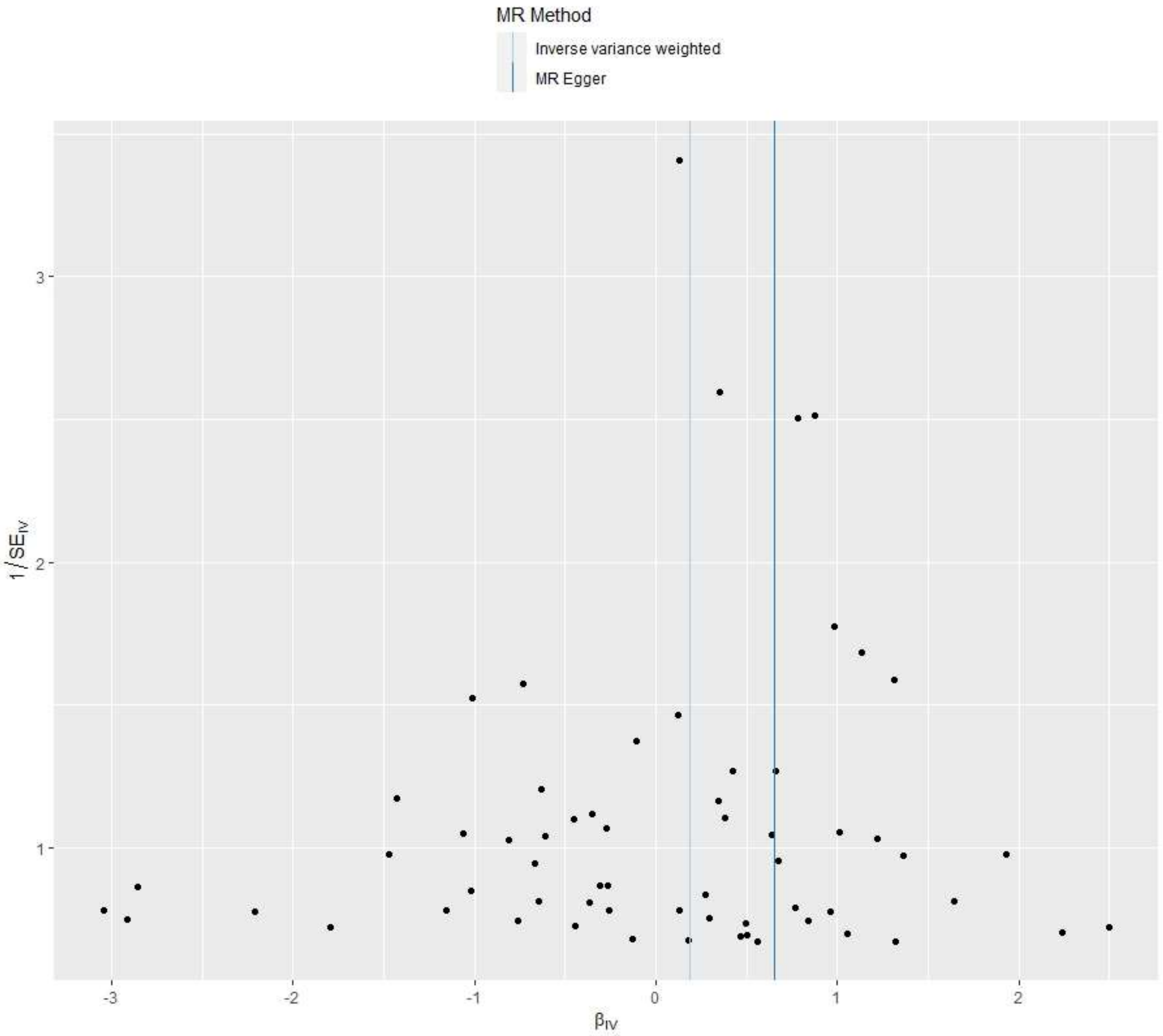
MR Method
Inverse variance weighted
MR Egger
3
2
1
$1/SE_{IV}$
-3
-2
-1
0
1
2
β_{IV}

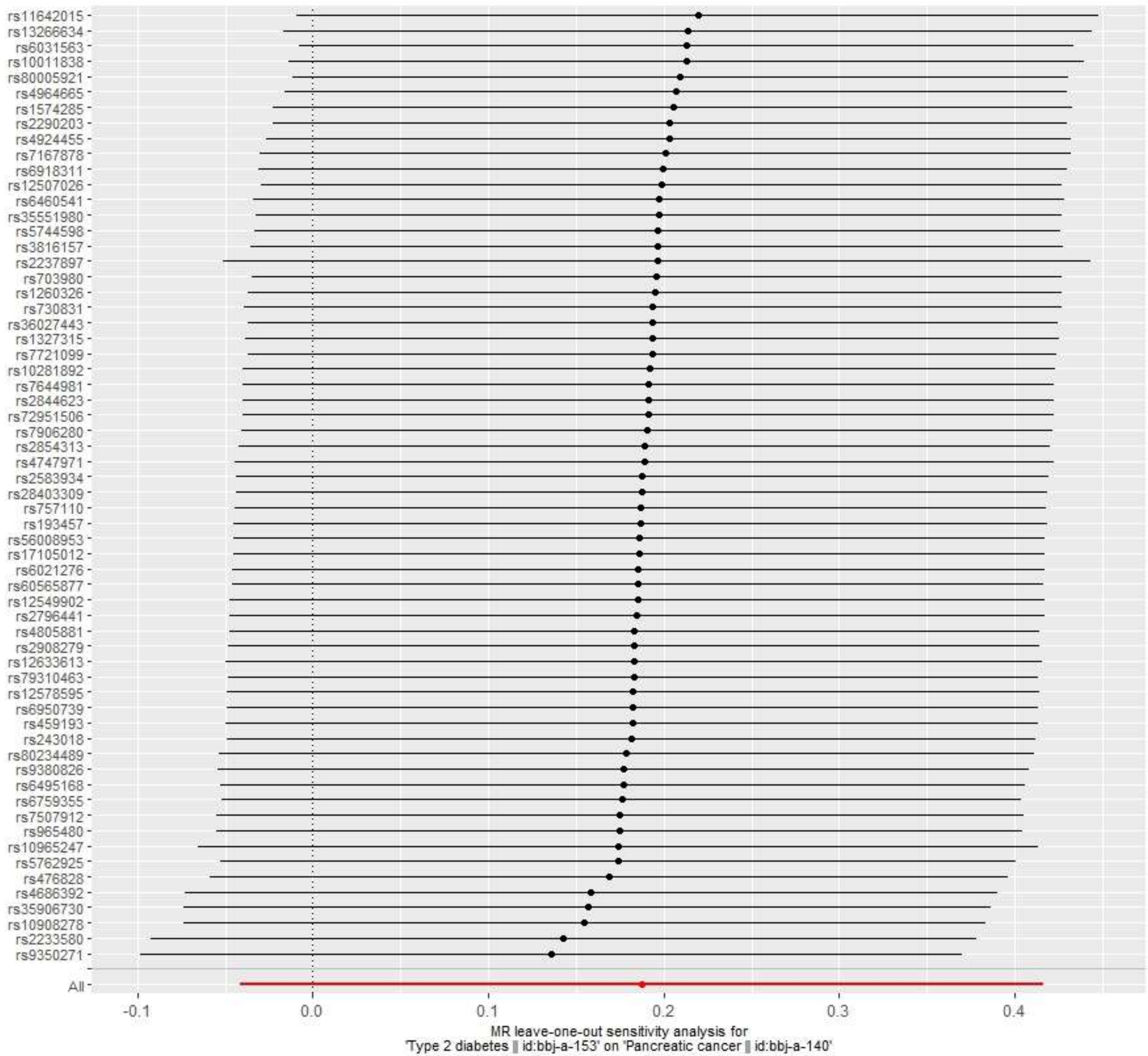
rs11642015
rs13266634
rs6031563
rs10011838
rs80005921
rs4964665
rs1574285
rs2290203
rs4924455
rs7167878
rs6918311
rs12507026
rs6460541
rs35551980
rs5744598
rs3816157
rs2237897
rs703980
rs1260326
rs730831
rs36027443
rs1327315
rs7721099
rs10281892
rs7644981
rs2844623
rs72951506
rs7906280
rs2854313
rs4747971
rs2583934
rs28403309
rs757110
rs193457
rs56008953
rs17105012
rs6021276
rs60565877
rs12549902
rs2796441
rs4805881
rs2908279
rs12633613
rs79310463
rs12578595
rs6950739
rs459193
rs243018
rs80234489
rs9380826
rs6495168
rs6759355
rs7507912
rs965480
rs10965247
rs5762925
rs476828
rs4686392
rs35906730
rs10908278
rs2233580
rs9350271
All
-0.1
0.0
0.1
0.2
0.3
0.4
MR leave-one-out sensitivity analysis for
'Type 2 diabetes || id:bbj-a-153' on 'Pancreatic cancer || id:bbj-a-140'
